# Cross-Scale Analyses of Animal and Human Gut Microbiome Assemblies from Metacommunity to Global Landscape

**DOI:** 10.1128/mSystems.00633-21

**Published:** 2021-07-06

**Authors:** Zhanshan (Sam) Ma

**Affiliations:** a Computational Biology and Medical Ecology Lab, State Key Laboratory of Genetic Resources and Evolution, Kunming Institute of Zoology, Chinese Academy of Sciences, Kunming, China; b Center for Excellence in Animal Evolution and Genetics, Chinese Academy of Sciences, Kunming, China; Southern Medical University

**Keywords:** animal gut microbiome, human gut microbiome, microbiome-host coadaptation, unified neutral theory of biodiversity, multisite neutral model, power analysis

## Abstract

Animal (human) gut microbiomes have been coevolving with their hosts for many millions of years. Understanding how the coevolution shapes the processes of microbiome assembly and diversity maintenance is important but rather challenging. An effort may start with the understanding of how and why animals and humans may differ in their microbiome neutrality (stochasticity) levels. Here, we attempted to perform layered comparative stochasticity analyses across animal species (including humans), class, and kingdom scales, corresponding to microbial metacommunity, landscape, and global-landscape scales. By analyzing 4,903 microbiome samples from 274 animal species covering 4 major invertebrate classes and all 6 vertebrate classes and including 1,787 human gut microbiome samples, we discovered the following: (i) at the microbial metacommunity (animal species) scale, although the general trend of stochasticity (measured in the relationships between fundamental biodiversity/dispersal numbers of Hubbell’s neutral theory and host species phylogenetic timeline) seems continuous, there seems to be a turning point from animals to humans in the passing rate of neutrality tests (12% to 45% versus 100%). We postulate that it should be the human experiences from agricultural/industrial activities (e.g., diet effects) and frequent social/familial contacts that are responsible for the dramatically rising stochastic neutrality in human gut microbiomes. (ii) At the microbial landscape (animal class) and global landscape (animal kingdom) scales, neutrality is not detectable, suggesting that the landscape is niche differentiated—animal species may possess “home niches” for their coadapted microbiomes. We further analyze the reliabilities of our findings by using variable *P* value thresholds (type I error) and performing power analysis (type II error) of neutrality tests.

**IMPORTANCE** Understanding how the coevolution (evolutionary time scale) and/or the interactions (ecological time scale) between animal (human) gut microbiomes and their hosts shape the processes of the microbiome assembly and diversity maintenance is important but rather challenging. An effort may start with the understanding of how and why animals and humans may differ in their microbiome neutrality (stochasticity) levels. Here, we attempted to perform layered comparative stochasticity analyses across animal species (including humans), class, and kingdom scales, corresponding to microbial metacommunity, landscape, and global-landscape scales by analyzing 4,903 microbiome samples from 274 animal species covering 4 major invertebrate classes and all 6 vertebrate classes, and including 1,787 human gut microbiome samples. The analyses were implemented by fitting the multisite neutral model and further augmented by checking false-positive and false-negative errors, respectively. It appears that there is a turning (tipping) point in the neutrality level from animal to human microbiomes.

## INTRODUCTION

Microbes represent the earliest life forms on earth, and early microbes evolved to be sufficiently complex and form spatially organized communities, as evidenced by distinguishable fossils up to 3.43 billion years ago. In contrast, fossil evidence has revealed that animals appeared much more recently during the Ediacaran Period at about 635 to 541 million years ago (1). It is therefore conceived that early evolution of kingdom *Animalia* would have happened in a “microbial soup,” leading to both pathogenic and symbiotic relationships between microbes and animals. A fundamental question about the coevolutionary soup is how the microbial community (i.e., the animal microbiome) is assembled and maintained. An alternative scenario, which proved to be rare in nature, would be that only one or even zero microbial species interacts with an animal, given that more than two species are required to constitute a community (2).

Addressing the question of community assembly and diversity maintenance, arguably the most essential ingredients of community structure and dynamics, has attracted extensive attention and also led to vigorous debates (3–6). Two leading and competing theories in this field have been the traditional niche theory with a history back to 1910s (7–10). Both theories were invented to explain a familiar phenomenon on earth, which was vividly described as follows by Charles Darwin (1859) in the last paragraph of his *On the Origin of Species*: “It is interesting to contemplate a tangled bank, clothed with many plants of many kinds, with birds singing on the bushes, with various insects flitting about, and with worms crawling through the damp earth, and to reflect that these elaborately constructed forms, so different from each other, and dependent upon each other in so complex a manner, have all been produced by laws acting around us.” With modern ecological terminology, entangled bank is essentially the concept of ecological community. Darwin was wondering how diverse lives (species) could coexist and form a beautiful entangled bank, while his theory stipulated the universal struggle for life as a consequence to natural selection. He was wondering since the seemingly universal struggle for life would most likely lead to monopoly by a few strongest competitors but would contradict what is usually observed in the entangled bank.

The classic niche theory assumed that each species has its own niche in which its individuals are adapted to live and prosper, and the entangled bank consists of many different niches suitable for many different species. In terms of niche theory, the deterministic traits a species possesses (or selective niche forces) play critical roles in driving the assembly of an ecological community as well as in maintaining the diversity after the community is established. In the late 1990s, Stephen Hubbell challenged the traditional theory view by proposing the unified neutral theory of biodiversity (UNTB) (10).

We realized that since the UNTB optimizes the fitting of the relative abundance to the predictions of the neutral model (11, 12), it might overestimate the true strength of neutral processes. Furthermore, some ecologists argue that even if empirical species abundance distributions (SADs) pass the neutrality test statistically, it does not guarantee that communities are truly neutral because nonneutral models can produce similar or even identical SAD patterns (13). To deal with these issues, we adopted the following three tactics to reinforce the robustness of our study and more importantly deepen our understanding of the focal question raised in the manuscript title.

First, we adopted what is considered a revolutionary computational advance in fitting the UNTB model, namely, the Harris et al. multisite neutral model (MSN) (14). This model greatly relieved a giant computational challenge in simultaneously estimating the migration rates among the local communities of a metacommunity since the computation problem is a so-termed nondeterministic polynomial hard problem (NP-hard problem). The NP-hard problem is inherently hard-to-compute optimum solutions since the required computational time grows exponentially with the problem size (i.e., the number of local communities in this case). The exponential magnitude means that when the problem is sufficiently large, it is infeasible (intractable) to obtain the optimum solutions even with the fastest supercomputers human can make because the computational time can be an astronomical number. This reality determines that the only feasible solution is to find suboptimal approximations to the solutions, even though they may not be easy to implement. The Harris et al. MSN model is such a solution that was implemented as an efficient Bayesian fitting framework by approximating the neutral models with the hierarchical Dirichlet process (HDP) (14). Harris et al. approximation captures the essential elements of the UNTB, i.e., neutrality, finite populations, and multisite metacommunity setting (14). For this reason, we term the Harris et al. HDP-neutral approximation framework as multisite neutral (MSN) model (14). We also realized that the adoption of *P* value in testing the neutral theory including the MSN model might introduce barely avoidable uncertainty in inferring conclusions. In this study, we report a series of *P* value thresholds for accepting the neutrality assumptions to obtain reliable common denominators. The variable thresholds of the *P* allow us to examine the influence of the statistical type I error (i.e., rejection of a true null hypothesis or obtaining a false-positive finding) in testing the null hypothesis of neutrality in animal and human gut microbiomes.

Second, we adopt a power analysis approach developed by Hammal et al. to cross-verify the results of the neutrality test with MSN (13). The power of the statistical test is measured with the probability that the null hypothesis is rejected when it is indeed false. The power analysis for the neutrality test is then aimed to deal with the previously mentioned argument—passing the neutrality test does not guarantee that the community is indeed neutral since nonneutral processes can still be the underlying mechanisms that generate the apparent neutral patterns (SADs). Hammal et al. showed that the presence of nonneutral processes is detectable as long as the sample size is sufficiently large and/or the effect size of nonneutral processes is strong enough in communities whose SAD patterns satisfy the neutral theory (13). Their power analysis (calculation) for neutral theory including the MSN model can be used to estimate an upper bound for the strength of nonneutral processes and therefore offer cross-verification for the neutrality test results based on the MSN model. In contrast with the previously mentioned variable *P* value threshold that is aimed to examine the influence of a type I error, the power analysis is designed to deal with a type II error (i.e., nonrejection of a false null hypothesis or obtaining a false-negative finding). To the best of our knowledge, the manuscript is likely the first study that simultaneously examines the influences of type I and II errors in testing the neutral theory with human and animal microbiome data sets.

Third, we put the neutrality analysis of the animal gut microbiome (AGM) and human gut microbiome in an evolutionary perspective by incorporating the evolutionary timeline (ET) or phylogenetic timeline (PT) of the host (animal) species into the analysis (15). The ET or PT is different from familiar phylogenetic distance (PD) in the literature. While PD refers to the divergence time for a pair of taxa, ET or PT refers to an evolutionary timeline for a taxon. The adoption of PT allows us to assess and interpret the ecological/evolutionary insights of the UNTB model parameters.

Understanding how animal (human) gut microbiomes are assembled is of critical significance both practically and theoretically. For example, if the stochastic (random) forces are indeed significant in maintaining microbiome structures, the design of human interventions to maintain or restore microbiome structures (such as, for example, fecal transplantation in medical practice and animal and human nutrition management) cannot ignore this important, innate aspect of the animal/human microbiomes. Theoretically, the hologenome theory of evolution treats the individual animal or plant as a holobiont or metaorganism consisting of the host and all of its symbiotic microbes (16, 17). The hologenome, a genome set carried by the holobiont, may be inherited from generation to generation with reasonable fidelity. The variations in the hologenome are subject to selection and drift effects evolutionarily (16–19). Nevertheless, in a recent review, Hammer et al. proposed that animals cover a continuum of dependence on the microbiome from symbiont-dependent species (such as aphids, humans, and cows) to species that lack beneficial symbionts (2). In the middle, there are species that are minimally or facultatively dependent on microbes. Therefore, the “strength” of intraholobiont (metaorganism) interactions between microbes and animal hosts may vary among animal species. These theoretical advances suggest that the coevolution between animal (human) microbiomes and hosts is beyond coadaptations on an ecological time scale. One side result of the present study was to construct mechanistic hypotheses for the coadaptations on an ecological time scale across community, metacommunity, and global landscape spatial scales.

## RESULTS

**FIG 1:**
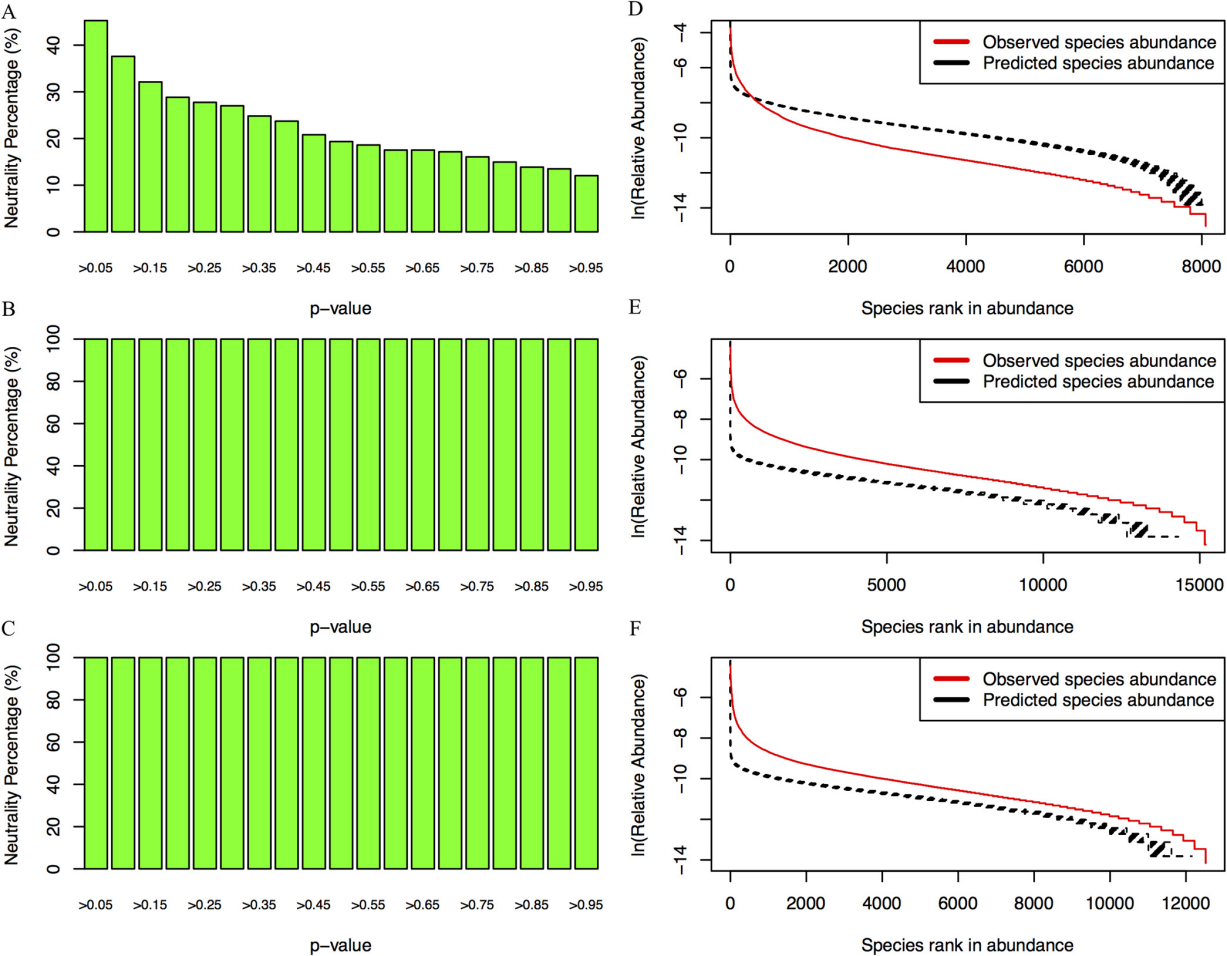
Passing percentages of MSN neutrality testing and model fitting example. (A to C) Passing percentages under different *P* value thresholds; animal gut microbiome, ranged 12% to 45% depending on the *P* value thresholds (A), American gut project, 100% for all thresholds (B); and Chinese gut project (100% for all thresholds) (C). (D to F) Examples of fitting the MSN model; host species level, successful fitting with the dwarf chimpanzee gut microbiome (D); host class level with *Mammalia* (E); and host *Animalia* kingdom level (F).

**FIG 2:**
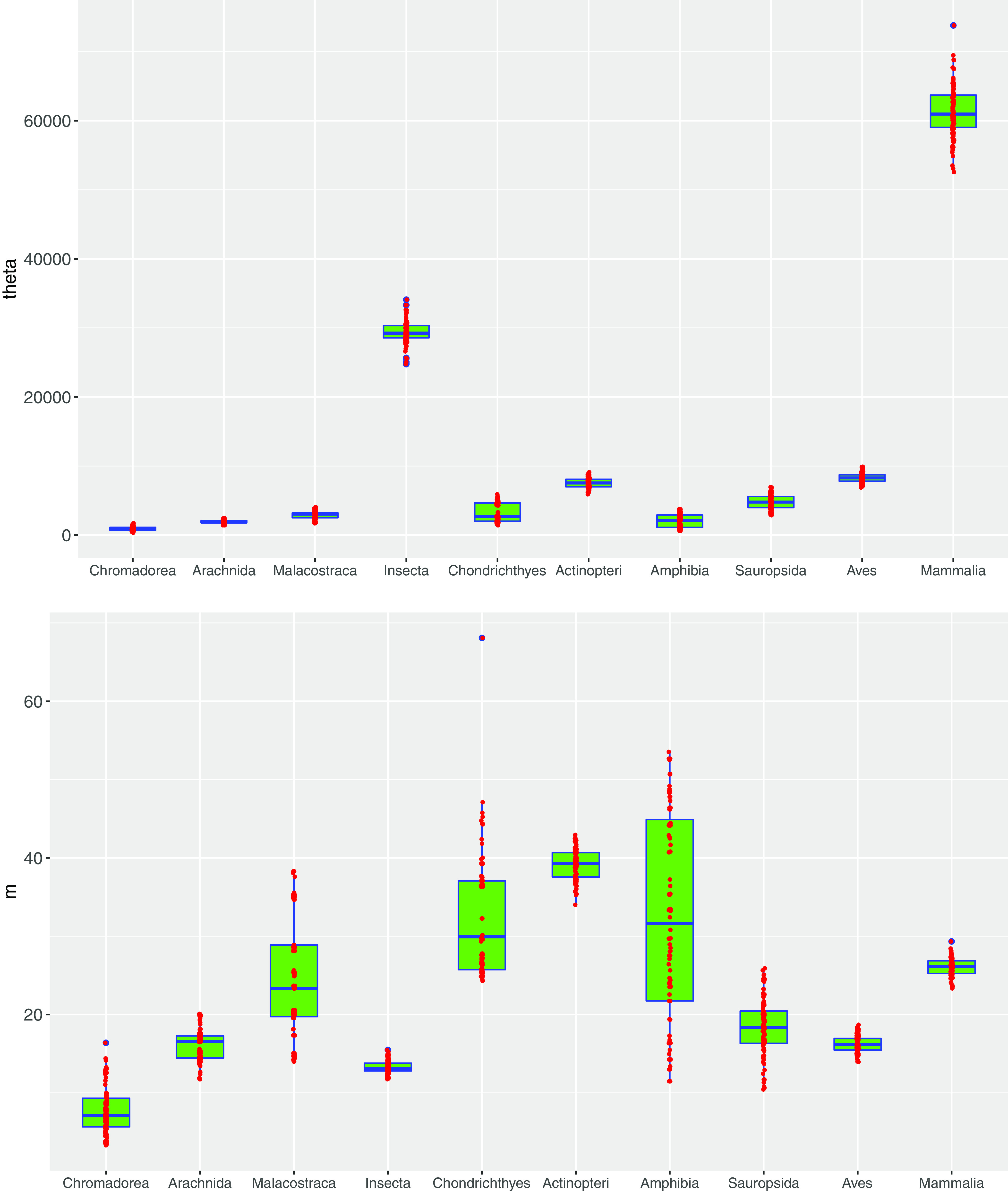
The box plots show the fundamental biodiversity number (*θ*) (top graph) and fundamental dispersal number (*M*) (bottom graph) computed from fitting the MSN model to the AGM at the animal class scale. Three standard summary numbers (statistics) of the parameters *θ* and *M*, including the first quartile (bottom edge of the rectangle), median (the inside segment), third quartile (top edge of the rectangle), were displayed. The “whiskers” above and below the box (rectangle) show the location of the minimum and maximum. The interquartile range (IQR) (showing the range of variation) is displayed by the height of the box, and the median shows the typical value. Outliers (<3 times IQR or >3 times IQR) are displayed outside the box. The smaller red points display the estimated values of *θ or M* from each of the 100 times of resampling (within each class) for fitting the MSN models. Notably, the IQR of *θ* seems narrower than that of *M*, indicating that the variation of *M* is much larger than the variation of *θ* or that within animal class the variability of dispersal limitation is much larger than that of the microbiome diversity. This result is expected since the former should strongly depend on the behavior and life styles of hosts, and the latter should mostly depend on the inner microenvironments of guts, which should be relatively stable. Insects, birds, and mammals exhibited the narrowest ranges of variations in *M*, and amphibians exhibited the highest variations in *M*. However, insects and mammals also exhibited the highest variations in *θ.* We do not yet understand the underlying mechanisms for the differences.

Table 1 shows a summary (the mean and standard error) of model-fitting parameters, and Table 2 shows the passing percentage under various *P* value thresholds for neutrality tests (i.e., new discovery indistinguishable from MSN predictions); both are summarized from Tables S2 to S4 (for species level), Table S5 (for class), and Table S6 (for kingdom) in the supplemental material. Fig. 1 illustrates the passing percentage and model-fitting examples at species, class, and kingdom levels. Fig. 2 displays the box charts for the distributions of fundamental biodiversity and dispersal numbers, which are two critical parameters of the neutral theory, and demonstrates the differences among 10 animal classes. Tables S7 and S8 in the supplemental materials list the results from analyzing the relationships between neutral theory parameters and species phylogenetic timeline (PT), as well as a logistic regression (LR) model for predicting neutral status with PT.

**TABLE 1:** Summary of the MSN model parameters of the AGM and human gut microbiomes (AGP and CGP data sets) at different scales^*a*^^,^^*b*^

Parameter	*θ*	*M* value	Metacommunity			Local community		
			*N_M_*	*N*	*P_M_*	*N_L_*	*N*	*P_L_*
Animal gut microbiomes at host species level (averaged from Table S2)								
Mean from (Table S2)	2,502.200	85.380	582.600	2,494.9	0.231	295.3	2,494.9	0.116
SE (*n *= 274)	620.126	23.841	53.288	4.237	0.021	33.012	4.237	0.013
Human gut microbiomes at host species level (averaged from Table S3 and S4)								
Mean AGP (Table S3)	1,264.592	162.425	2,499.970	2,487.8	1.000	2,500	2,487.8	1.000
SE (*n *= 1473)	3.829	0.747	2.559	2.551	0.000	2.551	2.551	0.000
Mean CGP (Table S4)	290.004	223.066	2,500	2,500	1.000	2500	2,500	1.000
SE (*n *= 314)	0.708	1.407	0.000	0.000	0.000	0.000	0.000	0.000
Animal gut microbiomes at host class level (averaged from Table S5)								
Mean (*Chromadorea*)	910.441	7.785	35.5	2,500	0.014	54.4	2,500	0.022
SE (*n *= 100)	30.469	0.296	4.2	0.0	0.002	5.5	0.0	0.002
Mean (*Arachnida*)	1,945.846	15.912	1.4	2,500	0.000	4.3	2,500	0.002
SE (*n *= 100)	22.464	0.204	0.3	0.0	0.000	0.9	0.0	0.000
Mean (*Malacostraca*)	2,846.621	24.420	3.2	2,500	0.001	8.5	2,500	0.003
SE (*n *= 100)	59.529	0.736	0.4	0.0	0.000	0.8	0.0	0.000
Mean (*Insecta*)	29,362.823	13.313	0.0	2,500	0.000	0.0	2,500	0.000
SE (*n *= 100)	172.368	0.079	0.0	0.0	0.000	0.0	0.0	0.000
Mean (*Chondrichthyes*)	3,310.259	32.358	0.2	2,500	0.000	1.6	2,500	0.001
SE (*n *= 100)	138.257	0.751	0.1	0.0	0.000	0.3	0.0	0.000
Mean (*Actinopteri*)	7,539.848	39.140	0.0	2,500	0.000	0.0	2,500	0.000
SE (*n *= 100)	72.882	0.195	0.0	0.0	0.000	0.0	0.0	0.000
Mean (*Amphibia*)	2,043.310	32.364	5.0	2,500	0.002	10.5	2,500	0.004
SE (*n *= 100)	96.289	1.331	1.0	0.0	0.000	1.9	0.0	0.001
Mean (*Sauropsida*)	4,744.392	18.367	0.0	2,500	0.000	0.2	2,500	0.000
SE (*n *= 100)	101.648	0.355	0.0	0.0	0.000	0.1	0.0	0.000
Mean (*Aves*)	8,305.193	16.193	0.0	2,500	0.000	0.0	2,500	0.000
SE (*n *= 100)	66.301	0.105	0.0	0.0	0.000	0.0	0.0	0.000
Mean (*Mammalia*)	61,250.292	26.107	0.0	2,495.9	0.000	0.0	2,495.9	0.000
SE (*n *= 100)	374.891	0.118	0.0	4.1	0.000	0.0	4.1	0.000
Animal gut microbiomes at animal kingdom level (averaged from Table S6)								
Mean	44,440.000	22.939	5.5	2,495.5	0.000	5.5	2,495.5	0.000
SE (*n *= 100)	664.452	0.179	2.794	2.799	0.000	2.794	2.799	0.000

a A total of 2,500 Gibbs samples were selected from 50,000 simulated communities (the first 25,000 simulations were discarded as the burn-in). *θ*, is the median of biodiversity parameters computed from 25,000 times of simulations; *M* value, the average medians of the migration rates of local communities in each metacommunity, also computed from 25,000 times of simulations; *N_M_*, the number of simulated neutral metacommunity samples with their likelihoods not exceeding the actual likelihood (i.e., *L* ≤ *L_0_* where *L* and *L_0_* are the simulated and actual likelihood, respectively); *P_M_* = *N_M_*/*N*, the pseudo *P* value for testing the neutrality at metacommunity level. Traditionally, if *P_M_* is >0.05, the metacommunity is considered indistinguishable from the pattern predicted by MSN model. Similarly, at the local community level, *N_L_* is the number of simulated local community samples with their likelihoods not exceeding the actual likelihood (i.e., *L* ≤ *L_0_*); *P_L_* = *N_L_*/*N*, the pseudo *P* value for testing the neutrality at the local community level. If *P_L_* is >0.05, the local community satisfies the neutral model. Due to a typo error in Harris et al. (14), the *P_M_* values exhibited here are adjusted as *P_M_* = 1 − *P_MS_*, where *P_MS_* is the output from the Harris et al. (14) computational program. Similarly, the *P_L_* values are adjusted as *P_L_* = 1 − *P_LS_*, where *P_LS_* is the output from their computational program. See online at https://arxiv.org/abs/1410.4038 for the latest update of Harris et al.

b While the standard practice for setting the threshold *P* value (*P_M_* and *P_L_*) for testing neutral theory has been a *P* value of 0.05 in most cases (e.g., reference 14), in the present study, we set *P* values ranged from 0.05 to 0.95 (see Table 2 and Fig. 1 for the effects of different *P* value thresholds on the neutrality passing percentages). Essentially, it is hoped that the readers can make their own judgments based on the presented results (see Table 2 and Fig. 1 for further illustrations).

**TABLE 2:** Passing percentages of the MSN neutrality tests with different *P* value thresholds at different levels, as well as the *P* values from Fisher’s exact tests

*P* value threshold	Passing percentages with different *P* values for:^*a*^											
	Animal gut microbiomes						Human gut microbiomes					
	Species		Class		Kingdom		AGP		CGP		Fisher’s exact test for *P_M_*^*b*^	
	*P_M_*	*P_L_*	*P_M_*	*P_L_*	*P_M_*	*P_L_*	*P_M_*	*P_L_*	*P_M_*	*P_L_*	Species vs AGP	Species vs CGP
0.05	45.3	37.6	0	0	0	0	100	100	100	100	<0.001	<0.001
0.10	37.6	27.4	0	0	0	0	100	100	100	100	<0.001	<0.001
0.15	32.1	23.4	0	0	0	0	100	100	100	100	<0.001	<0.001
0.20	28.8	18.2	0	0	0	0	100	100	100	100	<0.001	<0.001
0.25	27.7	15.3	0	0	0	0	100	100	100	100	<0.001	<0.001
0.30	27.0	12.0	0	0	0	0	100	100	100	100	<0.001	<0.001
0.35	24.8	9.5	0	0	0	0	100	100	100	100	<0.001	<0.001
0.40	23.7	8.8	0	0	0	0	100	100	100	100	<0.001	<0.001
0.45	20.8	8.0	0	0	0	0	100	100	100	100	<0.001	<0.001
0.50	19.3	6.2	0	0	0	0	100	100	100	100	<0.001	<0.001
0.55	18.6	5.8	0	0	0	0	100	100	100	100	<0.001	<0.001
0.60	17.5	5.5	0	0	0	0	100	100	100	100	<0.001	<0.001
0.65	17.5	4.7	0	0	0	0	100	100	100	100	<0.001	<0.001
0.70	17.2	4.4	0	0	0	0	100	100	100	100	<0.001	<0.001
0.75	16.1	4.0	0	0	0	0	100	100	100	100	<0.001	<0.001
0.80	15.0	4.0	0	0	0	0	100	100	100	100	<0.001	<0.001
0.85	13.9	3.3	0	0	0	0	100	100	100	100	<0.001	<0.001
0.90	13.5	2.9	0	0	0	0	100	100	100	100	<0.001	<0.001
0.95	12.0	2.6	0	0	0	0	100	100	100	100	<0.001	<0.001

a *P_M_* and *P_L_* columns list the passing percentages corresponding to different *P* value thresholds (the first column) for determining the neutrality at the metacommunity (*P_M_*) and local community (*P_L_*), respectively.

b The last columns list the *P* values from Fisher’s exact tests for detecting the differences between animals and humans in their gut microbiome neutrality.

10.1128/mSystems.00633-21.2 TABLE S2 The results of fitting the MSN model at the host species level or microbial metacommunity scale (i.e., all AGM samples from the same animal species constitute a metacommunity for the host species). Download Table S2, PDF file, 0.4 MB. Copyright © 2021 Ma. 2021 Ma https://creativecommons.org/licenses/by/4.0/ This content is distributed under the terms of the Creative Commons Attribution 4.0 International license.

10.1128/mSystems.00633-21.5 TABLE S5 Fitting the MSN model at host class level (microbiome landscape level): For each class, one AGM sample was randomly taken from all species of the same class and all obtained samples were treated as a metacommunity for fitting the MSN for the specific class; the fitting process was repeated 100 times for each class, and only the samples without exceeding 30,000 reads were included in the sampling due to the computational limitation of the MSN model. Download Table S5, PDF file, 0.8 MB. Copyright © 2021 Ma. 2021 Ma https://creativecommons.org/licenses/by/4.0/ This content is distributed under the terms of the Creative Commons Attribution 4.0 International license.

10.1128/mSystems.00633-21.6 TABLE S6 Fitting the MSN model at the microbiome global landscape level (or animal host kingdom level). A total of 100 AGM samples were randomly taken (1 from each of the 153 animal host species with PD information available) and used to fit the MSN model, and the fitting was repeated 100 times (i.e., a total of 100 MSN models were obtained). Download Table S6, PDF file, 0.1 MB. Copyright © 2021 Ma. 2021 Ma https://creativecommons.org/licenses/by/4.0/ This content is distributed under the terms of the Creative Commons Attribution 4.0 International license.

10.1128/mSystems.00633-21.7 TABLE S7 The Spearman’s correlation coefficients (*R*) between the MSN model parameters (*θ* and *M*) and the phylogenetic timeline (PT) of 179 host animal species or 10 animal classes. Download Table S7, PDF file, 0.1 MB. Copyright © 2021 Ma. 2021 Ma https://creativecommons.org/licenses/by/4.0/ This content is distributed under the terms of the Creative Commons Attribution 4.0 International license.

10.1128/mSystems.00633-21.8 TABLE S8 The classification table from the logistic regression (LR) analysis of the MSN parameters at the host species level (*X* = PT, *Y* = probability of nonneutral [*0*] versus neutral [1]). Download Table S8, PDF file, 0.1 MB. Copyright © 2021 Ma. 2021 Ma https://creativecommons.org/licenses/by/4.0/ This content is distributed under the terms of the Creative Commons Attribution 4.0 International license.

10.1128/mSystems.00633-21.3 TABLE S3 Fitting the MSN model with the American gut microbiome project (AGP) datasets. A total of 100 samples were taken randomly and constitute the metacommunity and then used to fit the MSN model, and the fitting process was repeated 100 times (i.e., a total of 100 MSN models were obtained). Download Table S3, PDF file, 0.1 MB. Copyright © 2021 Ma. 2021 Ma https://creativecommons.org/licenses/by/4.0/ This content is distributed under the terms of the Creative Commons Attribution 4.0 International license.

10.1128/mSystems.00633-21.4 TABLE S4 Fitting the MSN model with the Chinese gut microbiome project (CGP) datasets. A total of 100 samples were taken randomly and constitute the metacommunity and then used to fit the MSN model, and the fitting process was repeated 100 times (i.e., a total of 100 MSN models were obtained). Download Table S4, PDF file, 0.1 MB. Copyright © 2021 Ma. 2021 Ma https://creativecommons.org/licenses/by/4.0/ This content is distributed under the terms of the Creative Commons Attribution 4.0 International license.

### Neutrality analysis at the microbial metacommunity (animal species) scale.

At the animal (host) species or microbial metacommunity scale, all animal gut microbiome (AGM) samples from the individuals (hosts) of the same animal species are treated as a microbial metacommunity. A total of 274 metacommunities for 274 animal species are separately fitted to the MSN models for testing the neutrality at the animal species level (Table S2). The American gut microbiome project (AGP) and Chinese gut microbiome project (CGP) data sets of 1,473 American and 314 Chinese adults are fitted to the MSN model to represent the species of Homo sapiens (Table S3 and S4). Table 1 shows the summarized parameters from Tables S2 to S4.

As displayed in Table 2 and Fig. 1, the passing percentage for the neutrality tests at the metacommunity level range from 12.0% to 45.2% depending on the *P* value thresholds (0.05 to 0.95) chosen for rejecting the null hypothesis or inferring new discovery. Strictly speaking, the term passing percentage that we use throughout the article is not accurate since it only indicates that certain percentages of metacommunities are indistinguishable from the theoretical prediction of the MSN model. Both ends of the previously mentioned range of 12.0% to 45.2% correspond to *P* values of 0.95 and 0.05, respectively, with the former being the strictest and the latter the least strict criteria. Fig. 1A shows the passing percentages corresponding to different thresholds of the *P* value for the MSN neutrality tests at the animal host species or microbial metacommunity level. Fig. 1D shows the successful fitting of the MSN model to the gut microbiome of dwarf chimpanzee (*Pan paniscus*) as an example of host species level modeling; when the *P* value is >0.95, the gut microbiome metacommunity of this primate is indistinguishable from that predicted by the MSN model.

While the neutrality rates of AGM above indicate “partial” neutrality (12.0% to 45.2%) or a limited role of neutral forces in shaping the animal gut microbiomes at the host species level, the neutrality levels demonstrated by the AGP and CGP data sets are 100%, across all *P* value thresholds adopted (Table 2, Table S4 to S5, Fig. 1). That is, compared with animal gut microbiomes, human gut microbiomes seem to possess a significantly higher neutrality level and that stochastic neutral forces play a significantly larger role in the assembly and diversity maintenance of human gut microbiomes. Results of a Fisher’s exact test confirmed the statistical significances of their differences in neutrality percentages (20). As shown in Table 2, the differences were significant across all *P* value thresholds, from the least strict (*P* = 0.05) and to the most strict (*P* = 0.95). Across all the thresholds, the *P* values from the Fisher’s exact test are less than 0.001.

### Neutrality analysis at the microbial landscape (animal class) and global landscape (animal kingdom) levels.

At the animal (host) class taxon or microbial landscape scale, all AGM samples from same animal class are treated as a supermetacommunity or microbial (microbiome) landscape. As shown in Tables 2 and 3 (see Table S5 for the detailed results), the passing percentages of neutrality tests for all animal classes were nil. Our interpretation for this lack of neutrality at the animal class or microbial landscape level is that the animal host species within a class may form different niches for their own gut microbiomes. In other words, each animal species may exert host-specific selection to its gut microbiome, resulting in a nonneutral pattern at the host class or microbial landscape scale.

**TABLE 3:** Power tests for selected data sets with PC^*a*^ and IF^*b*^ models

Scale^*c*^	Metacommunity^*d*^	*P_M_* ^*e*^	*P_L_* ^*e*^	PC_power_^*f*^		IF_power_^*f*^	
				Power_*M*_	Power_*L*_	Power_*M*_	Power_*L*_
Species	Caenorhabditis remanei	0.564	0.328	0.050	0.033	0.217	0.433
	Spider	0.666	0.691	0.267	0.317	0.733	0.583
	Callinectes sapidus	0.254	0.292	0.033	0.267	0.283	0.217
	Acanthocinus aedilis	0.001	0.000	0.517	0.733	0.350	0.683
	Galeocerdo cuvier	0.396	0.347	0.217	0.333	0.333	0.017
	Carassius auratus	0.004	0.006	0.383	0.217	0.650	0.583
	Dendropsophus minutus	0.375	0.255	0.783	0.350	0.833	0.717
	Chelonia mydas	0.000	0.000	0.317	0.417	0.367	0.383
	Duck	0.000	0.002	0.283	0.533	0.100	0.250
	Pan paniscus	1.000	0.000	0.067	0.167	0.017	0.217
Classes (landscape)	Chromadorea	0.003	0.009	0.517	0.750	0.100	0.517
	Arachnida	0.000	0.000	0.433	0.450	0.050	0.333
	Malacostraca	0.002	0.007	0.817	0.483	0.833	0.617
	Insecta	0.000	0.000	0.233	0.300	0.400	0.933
	Chondrichthyes	0.000	0.000	0.333	0.517	0.883	0.317
	Actinopteri	0.000	0.000	0.650	0.817	0.250	0.067
	Amphibia	0.001	0.005	0.400	0.350	0.233	0.450
	Sauropsida	0.000	0.000	0.217	0.167	0.833	0.783
	Aves	0.000	0.000	0.483	0.467	0.150	0.750
	Mammalia	0.000	0.000	0.333	0.183	0.967	0.750
Global landscape	Randomly sampled	0.001	0.001	0.583	0.767	0.033	0.633
AGP	Randomly sampled	1.000	1.000	0.083	0.150	0.783	0.267

a *c *= 1.

b *k *= 0.01.

c Displays the three scales and the fourth test (with human AGP data set).

d Further specifies the metacommunity samples from each of the three scales and the AGP.

e *P* values from regular MSN neutrality testing performed and explained in previous sections.

f The last two columns are each further divided into two subcolumns for metacommunity and local community, respectively and represent the power values computed for PC and IF nonneutral models, respectively. Notice the opposite trend between the P values and power values, which indicates that the findings from MSN neutral testing and corresponding power analysis are consistent because small power value indicates weak nonneutral or strong neutral process (large *P* value from neutrality test).

Similarly, at the animal (host) kingdom or microbial global landscape scales, all AGM samples from the animal kingdom are treated as a supersupermetacommunity or microbial (microbiome) global landscape. As shown in Table 1 and 2 (see Table S6 for the detailed results), the passing percentage of neutrality at the global landscape level was nil again. Our interpretation for the lack of neutrality at the global landscape level (animal kingdom) is similar to the previous postulation for the landscape level (animal class)—each animal species in the animal kingdom may have species-specific “home” niche.

### Power analysis of the MSN neutrality tests.

Table 3 lists the power calculation results for selected metacommunity samples at three focal scales of this study, namely, the animal species (microbial metacommunity), animal class (microbial landscape), and animal kingdom (microbial global landscape) scales. The first column displays the three scales and the fourth test (with human AGP data set). The second column further specifies the metacommunity samples from each of the three scales and the AGP. The third and fourth columns are the *P* values from regular MSN neutrality testing performed and explained in previous sections. The last two columns (each further divided into two subcolumns for metacommunity and local community) represent the power values computed for Pigolotti and Cencini's model (PC) and intrinsic fitness (IF) nonneutral model. Note the opposite trend between the *P* values from regular MSN neutrality tests and the power values from the power analysis, which indicates that the findings from both MSN-neutral testing and corresponding power analysis are consistent because a small power value means weak nonneutral process (or strong neutral process) and consequently a large *P* value of the neutrality test.

For example, at the animal host class scale (microbial landscape scale), the power values for 10 animal classes ranged from 0.217 to 0.817 at metacommunity scale and ranged from 0.167 to 0.817 at local community scale indicate strong nonneutral process captured by nonneutral PC model. That is, the alternative nonneutral (PC) hypothesis was accepted and the null neutral hypothesis was rejected. The regular or standard MSN-neutral testing for the same samples of the 10 animal classes also rejected the neutral null hypothesis (*P* value ranged from 0.000 to 0.003 for the metacommunity scale and 0.000 to 0.009 at the local community scale). The findings from MSN neutrality testing and power analysis on other scales are also consistent in majority of the cases tested. The behavior with the IF model is slightly more complicated at the animal species scale, as expected by reference 13. In conclusion, the power analysis with the Hammal et al. approach confirms that the MSN neutrality tests are reasonably reliable (13, 14).

### The evolution of the fundamental biodiversity and dispersal numbers.

Table S7 exhibits Spearman’s correlation coefficients (*R*s) between the animal host phylogenetic timeline (PT) and the neutral theory parameters, including the fundamental biodiversity number (*θ*) and fundamental dispersal number (*M*). The *M* in the MSN model is the immigration rate—measured as the average individuals immigrated from the metacommunity (that the local community belongs to) to the local communities (14). The *θ* is the rate at which new individuals are appearing in the metacommunity as a result of speciation. Fig. 2 shows the box charts of *M* and *θ*, computed at the animal class level.

At the animal host species level, the negative Spearman’s correlation (*R* = −0.326, *P* < 0.001) between PD and *M* suggests that the gut microbiome in more recent animal host species exhibits a higher immigration rate among individuals of the same animal species. Furthermore, the human is not an exception. However, at the host class level, the correlation is insignificant statistically (*P* = 0.515). The loss of statistical significance is likely because the class scale is too coarse to exhibit significant correlation or, alternatively, the neutral model failed at the class level as explained previously.

In addition, the negative correlation (*R* = −0.122, *P* = 0.10) between PT and *θ* also suggests that the more recent animal species host higher gut microbiome biodiversity. The positive correlation between *M* and *θ* confirms that the more recent animal species may host not only higher gut microbiome diversity but also higher microbe exchange among individuals of the same animal host species. The human is not an exception since the incorporation of AGP and CGP data sets actually raised the correlation level and lowered the *P* value.

We further applied the logistic regression (LR) to analyze the relationship between the neutrality status (neutral = 1, nonneutral = 0; at the host species level) and the phylogenetic timeline (PT) of host species (Table S8). The top section of Table S8 shows that the PT alone was able to predict the neutrality status with a 65% precision level, which is somewhat remarkable. Furthermore, the positive regression coefficient for PT indicates that PT is positively correlated with nonneutrality, suggesting that more ancient animal species (with larger PT) are more likely to host nonneutral microbial communities. Consequently, more recent species such as primates (e.g., Fig. 1D of dwarf chimpanzee) and humans are more likely to host neutral gut microbiomes. This finding is obviously consistent with the previous comparative results between the AGM and AGP/CGP (Table 2).

**FIG 3:**
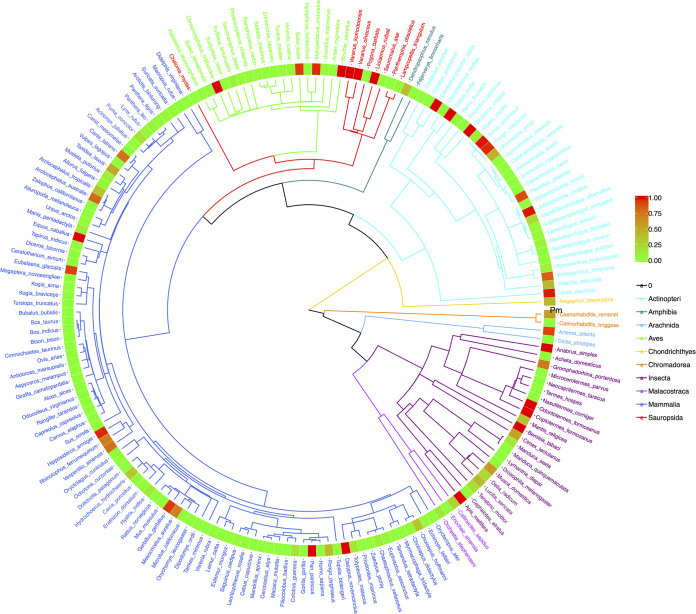
The phylogenetic tree of 179 animal species, annotated with the *P_m_* values from their MSN (multisite neutral model) testing at metacommunity level. (i) Branches and species labels constitute a standard phylogenetic tree and were colored differently for each of the 10 animal classes (each color of the branches represents an animal class, and species labels were colored in terms of their class identities). (ii) The band of mosaic color is a heatmap representing the size of the *P_m_* value. The *P_m_* value ranged between 0 (green) and 1 (red) and is used to determine the outcome of neutrality testing as explained in the manuscript. The closer the color is to red in the heatmap, the greater the *P_m_* value (the more likely being neutral); and the closer the color is to green, the smaller the value (the less likely being neutral).

**FIG 4:**
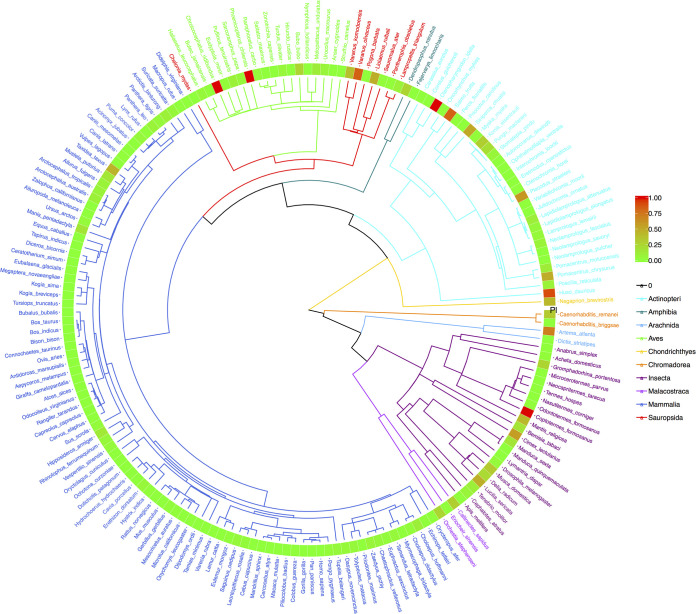
The phylogenetic tree of 179 animal species, annotated with their *P_l_* values from their MSN (multisite neutral model) testing at local community level. (i) Branches and species labels constitute a standard phylogenetic tree and were colored differently for each of the 10 animal classes (each color of the branches represents an animal class, and species labels were colored in terms of their class identities). (ii) The band of mosaic color is a heatmap representing the size of the *P_l_* value. The *P_l_* value ranged between 0 (green) and 1 (red) and is used to determine the outcome of neutrality testing as explained in the manuscript. The closer the color is to red in the heatmap, the greater the *P_l_* value (the more likely being neutral); and the closer the color is to green, the smaller the value (the less likely being neutral).

**FIG 5:**
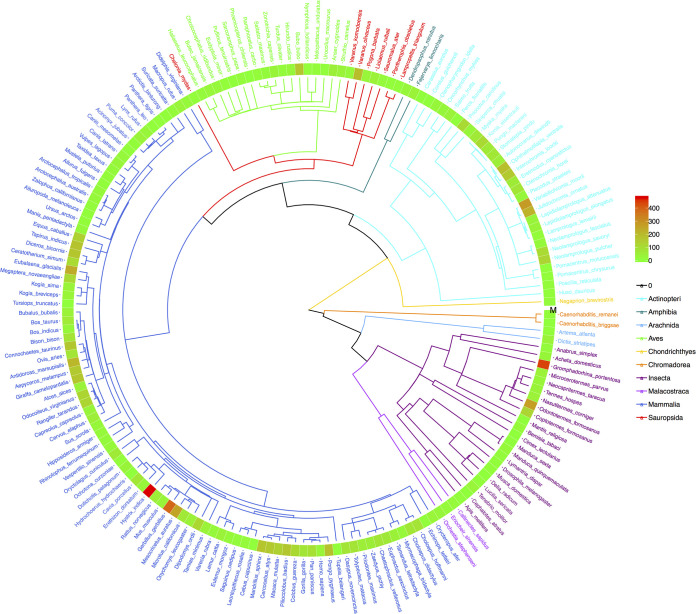
The phylogenetic tree of 179 animal species, annotated with the “average medians of the migration rates” (*M*) from their multisite neutral model (MSN) testing. (i) Branches and species labels constitute a standard phylogenetic tree and were colored differently for each of the 10 animal classes (species labels were colored in terms of their class identities). (ii) The mosaic color band (the tree terminal circle) is a heatmap representing the *M* values. The closer the color is to red in the heatmap, the greater the *M* value; and the closer the color is to green, the smaller the *M* value (see Table S7 for the relationship between *M* and phylogenetic timeline).

**FIG 6:**
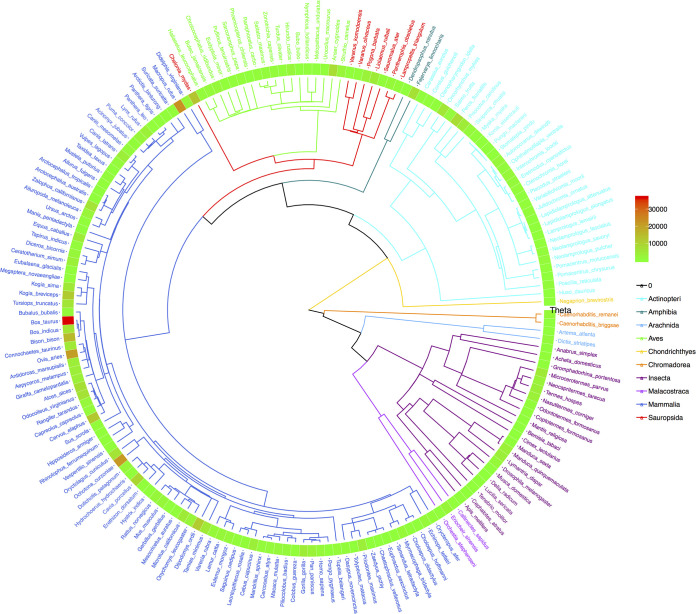
The phylogenetic tree (P-Tree) of 179 animal species, annotated with the fundamental biodiversity number (*θ*) from their multisite neutral model (MSN) testing. (i) Branches and species labels constitute a standard phylogenetic tree and were colored differently for each of the 10 animal classes (species labels were colored in terms of their class identities). (ii) The mosaic color band (the tree terminal circle) is a heatmap representing for the *θ* values. The closer the color is to red in the heatmap, the greater the *θ* value; and the closer the color is to green, the smaller the *θ* value (see Table S7 for the relationship between *θ* and phylogenetic timeline).

Finally, to illustrate the previously explained phylogenetic perspectives, we drew the four phylogenetic trees, annotated with P_*M*_ (Fig. 3), *P_L_* (Fig. 4), *M* (Fig. 5), and *θ* (Fig. 6). The phylogenetic trees annotated with *P_M_* (Fig. 3) and P_*L*_ (Fig. 4) illustrated the distributions of *P* values (from neutrality testing at metacommunity or local community levels) on the trees. Similarly, Fig. 5 illustrates the distribution of average medians of the migration rates (*M*), while Fig. 6 illustrated the distribution of fundamental biodiversity number (*θ*).

## DISCUSSION

Here, we summarize the conclusions from previous findings and further discuss their ecological/evolutionary mechanisms and implications.

First, at the animal host species or microbial metacommunity level, animals and humans appear to have evolved “continuously” in the sense that the MSN parameters (fundamental biodiversity and dispersal numbers) showed consistent patterns, i.e., more recent species are more likely to host the gut microbiomes with higher biodiversities and larger dispersal capabilities. Furthermore, more recent species such as humans are more likely to host neutral gut microbiomes.

Second, despite the “evolutionary continuity” stated above, the human gut microbiomes may have experienced a turning point—the neutral drifts in human gut microbiomes are so strong that the passing percentages of MSN neutrality tests reached 100%, whereas the percentages in animal gut microbiomes were less than one-half (12% to 45%). This finding is supported by a virtually full spectrum of confidence levels, as determined by the *P* value thresholds from 0.05 to 0.95. With the *P* value threshold of 0.95, the reliability or robustness of this finding is exceptionally strong.

Third, at the host class or kingdom levels, which correspond to microbial landscape or global landscape level, the neutrality in animal gut microbiomes was not detectable, as demonstrated by the zero passing percentage of MSN neutrality tests. We postulate that animal species should be able to evolve their species-specific niches for their own microbiomes. Globally, the different animal species together build a mosaic of different niches, leading to the loss of neutrality at the landscape or global landscape levels.

The above findings were sustained after further scrutiny of both type I and type II errors associated with the Harris et al. MSN neutrality tests (14). First, to deal with a type I error, usually the false-discovery rate (FDR) control is applied. However, the slightly unorthodox convention used in testing the neutral theory does not allow us to apply FDR adjustment, and we resolved the issue by using a series of *P* value thresholds. This resolution allows us to obtain the most conservative estimation of neutrality rates, i.e., minimizing type I error. Second, to deal with a type II error, we applied the Hammal et al. power analysis framework and concluded that the findings from the MSN neutrality tests are reliable (13). The power analysis is particularly useful because it confirmed that the neutral patterns revealed by the MSN modeling are indeed the product of neutral processes, rather than that of nonneutral selection forces such as competitions and/or inherent species fitness differences, as frequently criticized regarding applications of neutral theory (13). Power analysis also confirmed the existence of nonneutral competitions at the microbial landscape (animal class) and global landscape (animal kingdom) scales.

There have been extensive studies on the diversity of animal and human gut microbiomes (21–32), including the neutral theory testing and coevolution with the host animals. For example, the Sloan neutral model was applied to investigate neutrality in the animal microbiome and confirmed the applicability of the neutral model (11, 12), but the number of animal species tested was limited to approximately a dozen species (19, 33). For another example, Youngblut et al. collected over 400 gut microbiome samples from 180 different species and found that both the host phylogeny (evolutionary history) and diet have modulated different aspects of the microbiome diversity (32). They identified an intensified phylogenetic signal in *Mammalia* (versus non-*Mammalia*) evidenced by the existence of operational taxonomic units (OTUs) with local phylogenetic signals and revealed by so-termed cophylogeny analyses, which verified that the phylogenies of the host and symbiont (microbes) correspond in their branching patterns.

Finally, we discuss a limitation of this study. As shown in previous sections, despite extensive efforts, we could only reveal the relationships between neutral-theoretic parameters and the host phylogenetic timeline (PT), as well as the relationship between PT and passing rate of neutrality testing. Beyond these relationships, our study could not further shed light on the coevolution between animal gut microbiomes and their hosts. This limitation has to do with the difficulty of the problem. Yeoman et al. reviewed extensive literature then available on the evolution of animal-associated microbiomes (1). They proposed to classify evolutionary forces applied to animal microbiomes into three categories, including the primary intraspecific competition (e.g., mutation and horizontal gene transfer), secondary ecological interactions (e.g., competition, predation, and symbiosis), and tertiary host influences (e.g., host diet, phylogeny, and immune system). They suggested that modularity as an essential adaptive strategy to accommodate evolutionary complexity given that evolution is often considered a march toward increasing complexity. In particular, the cooperation prevalent in the microbiome may play an important role in shaping the modularity. Nevertheless, quantifying each category of evolutionary forces is hardly possible with state-of-the-art data in microbiome research. On a positive side, Zeng et al. and Zeng and Rodrigo developed an agent-based computational framework to simulate and analyze the dynamics of microbiome evolution (34, 35). Their framework includes neutral assumptions for both host genetics and microbiome demography, which allowed them to construct a minimalist null model to compare with the observed patterns of human microbiomes. An interesting result they obtained was that parental inheritance of hosts appears to lower microbial diversity and raise homogeneity within hosts, but ongoing environmental acquisitions do the opposite. Furthermore, the relationship between parental inheritance and interhost heterogeneity in the microbiome is nonlinear. Their framework allows for simulation analysis of the neutral dynamics of microbiomes within a host population under different transmission modes and shared environment (35). Scanlan also reviewed the relationship between microbial evolution and ecological opportunity (i.e., niche availability or niche differentiations) in the gut environment (36) and suggested that the development and application of experimental evolution approaches associated with genomic and metagenomic analyses can play a critical role in disentangling stochastic neutral drifts from selection. While we totally concur with Scanlan that carefully designed experimental evolution studies are critical (36), we also argue that neutral-theoretic analyses such as that in this study can offer an important baseline (null model) for designing and implementing the evolution experiments.

## MATERIALS AND METHODS

### Data sets of AGM and HGM.

The 16S rRNA sequencing read data sets of more than 6,900 samples of the animal gastrointestinal tract microbiome (AGM) were collected from 108 published studies, covering 5 phyla and 19 classes. To balance sample sizes and the distribution across taxa, the classes with less than 10 samples were excluded. The samples treated with antibiotics and some others that with spuriously small sample sizes were excluded from the analysis, and 4,903 samples from 10 classes were preserved. The 4,903 samples covered 3 primary phyla (*Nematoda*, *Arthropoda*, and *Chordata*), 10 classes (*Chromadorea*, *Arachnida*, *Malacostraca*, *Insecta*, *Chondrichthyes*, *Actinopteri*, *Amphibia*, *Sauropsida Aves*, and *Mammalia*), and 274 animal species. That is, the selected data sets cover all six classes of vertebrates and the two most important phyla (*Nematoda* and *Arthropoda*) of invertebrates and therefore are rather representative for the animal kingdom. Table S1 (MS-Excel file) in the supplemental material provided detailed sample information, including data accession numbers.

10.1128/mSystems.00633-21.1 TABLE S1 The list of animal gut microbiome (AGM) data accession numbers and the animal species in each study. Download Table S1, XLSX file, 0.04 MB. Copyright © 2021 Ma. 2021 Ma https://creativecommons.org/licenses/by/4.0/ This content is distributed under the terms of the Creative Commons Attribution 4.0 International license.

From the 16S rRNA raw sequencing reads, we utilized the QIIME 2 (version 2018.6.0) software package to compute the operational taxonomic unit (OTU) tables (37). Specifically, the 16S rRNA amplicon sequences were processed using tools (plugins) in QIIME 2 (version 2019-08). Specifically, we used the demux plugin to demultiplex reads. Then, the sequences were processed using the dada2 plugin with default settings, including denoising, dereplicating, and extracting features (amplicon sequence variants [ASVs]). Finally, we used feature-classifier (classify-sklearn plugin) for taxonomy classification, which compares the ASVs obtained in the previous step to the reference sequence database, and obtained the OTU tables.

Phylogenies were analyzed and visualized with the ape and ggtree R-packages (38, 39). The phylogenetic tree and phylogeny information were obtained online at http://timetree.org (15). Phylogenetic timeline (PT), which can be considered a proxy of the phylogenetic history of a taxon, or the “age” of a taxon with ancient taxa having larger PT values and recent (modern) taxa having smaller PT values, is harnessed to investigate the evolutionary implications of the neutral theory parameters.

The two human gut microbiome data sets were from the American gut project (AGP) (http://americangut.org/) and a Chinese gut microbiome project (CGP) reported by Zhang et al. (40, 41). The AGP sequenced the gut microbiome samples of 1,473 healthy American adults. A total of 14,355 OTUs (at 97% similarity level) were identified, and the average number of 16S rRNA reads per sample was 14,585. The CGP project sequenced the gut microbiome samples of 314 healthy Chinese adults covering 20 rural and urban cohorts from 7 ethnic groups throughout China. A total of 36,918 OTUs (at 97% similarity level) were identified, and the average number of 16S rRNA reads per sample was 14,035. The OTU tables from the AGP and CGP were obtained from their respective websites.

### General study design.

**FIG 7:**
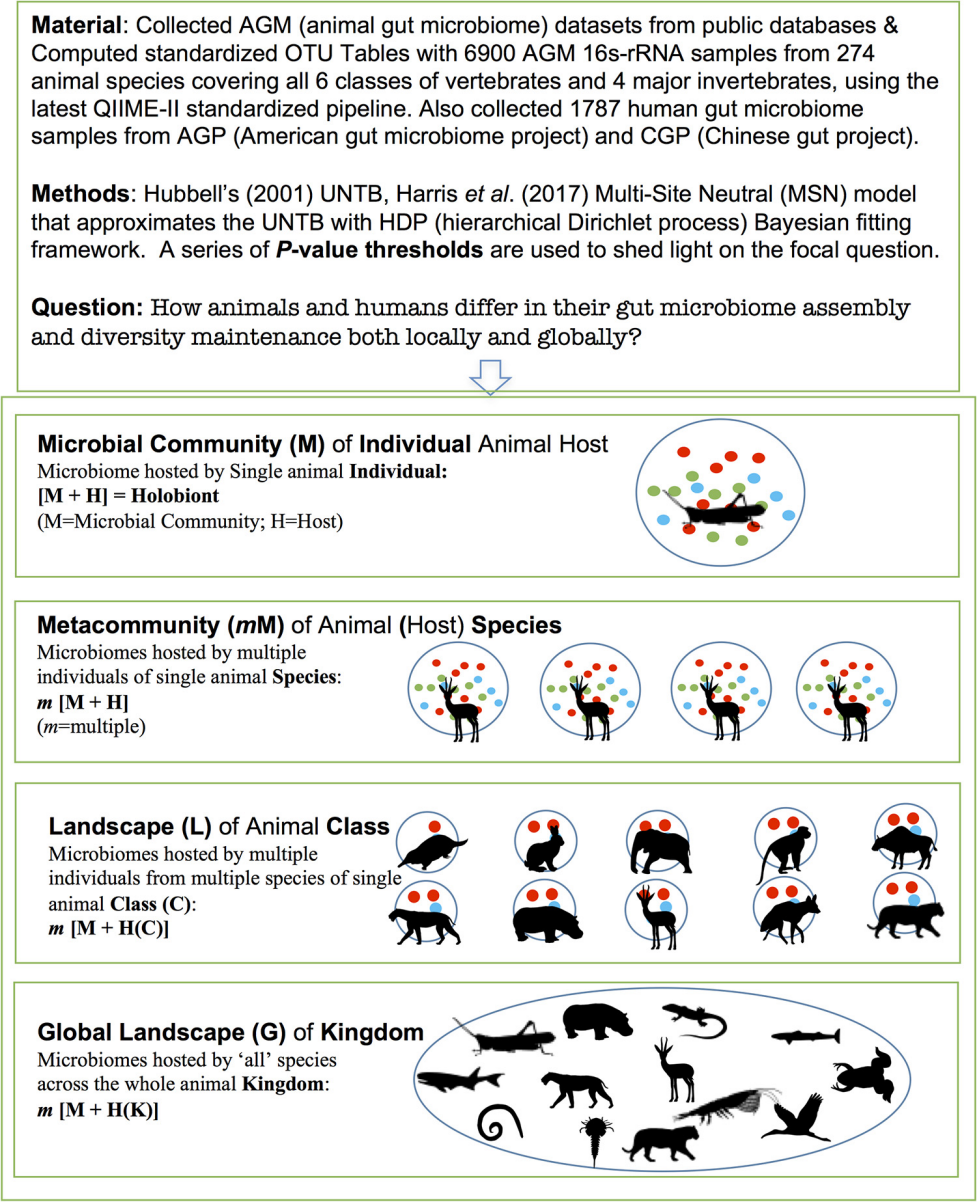
General study design for the data sets, questions answered, and approaches used in this study.

Table 4 and Fig. 7 summarize the general study design (strategy) we adopted for testing the UNTB-MSN model with the previously described AGM/AGP/CGP data sets, as well as the objectives we tried to achieve with the modeling analysis.

**TABLE 4:** The multiple scales for studying the animal/human gut microbiomes from the perspectives of the gut microbiome, animal host taxa, and microbiome-host complex^*a*^

Animal (host) scale	Microbiome scale	Components of (super)metacommunity	Holobiont scale
Individual	Microbial community	Single-animal individual host of a single species	M + H = holobiont
Species	Metacommunity	Multiple animal individuals hosts of single animal species	*m* [M + H]
Class	Landscape	Multiple animal individuals from multiple species of a single class	*m* [M + H(C)]
Kingdom	Global landscape	Multiple animal individuals from all species across the animal kingdom	*m* [M + H(K)]

a Also see Fig. 7. A total of 274 animal species belonging to 10 animal classes (covering all 6 vertebrates and 4 major invertebrates) were sampled for their gut microbiomes, and a total of 4,903 animal gut microbiome (AGM) samples were sequenced and the microbial OTU tables corresponding those samples were computed with QIIME II (see Table S1 for the detailed sample information, including data accession numbers). In addition, 1,473 human gut microbiome samples from the American gut microbiome project (AGP) and 314 samples from the Chinese gut microbiome project (CGP) were utilized to perform comparative analyses with the AGM data sets. All sequencing data were collected from public domain, and we computed only standardized OTU tables with QIIME II.

### MSN implementation of Hubbell’s UNTB.

Different from traditional niche theory, Hubbell’s UNTB was formulated as a probability distribution model (10), which can be fitted with the species abundance distribution (SAD) data, obtainable by sampling ecological communities, and rigorously tested statistically. The theory assumes that the individuals of all species in a community are demographically equivalent, but their birth/death rates are stochastic. Consequently, random drift and dispersal can play critical roles in driving community assembly and diversity maintenance. This conceptually distinguishes local community dynamics from metacommunity dynamics, of which both are postulated to be controlled by similar neutral processes—stochastic drifts in species demography, local speciation, and global dispersal (migration). The theory possesses two key parameters (elements), as follows: (i) the immigration rate (*M_i_*) that couples local community to the metacommunity and (ii) the speciation rate (*θ*, also named the fundamental biodiversity number), i.e., the rate at which new individuals are added to the metacommunity due to speciation. The UNTB assumes that the SAD of each community sample can be fitted with the multinomial (MN) distribution, which is parameterized by the immigration rate and speciation rate mentioned previously. Testing the UNTB model is then computationally equivalent to testing the goodness of fitting to the MN distribution.

Nevertheless, a fully general algorithm of fitting multiple sites with UNTB with different immigration rates is computationally intractable even for a small number of sites, which implies that when the problem size (the size of sites) becomes large, the running time of the algorithm becomes too time consuming to be useful. Consequently, to test the UNTB in true multisites (multiple local communities), approximate algorithms must be used. Harris et al. then developed an efficient Bayesian fitting framework for testing the UNTB by approximating the neutral models with the hierarchical Dirichlet process (HDP) (14). In the following text, we briefly outline the formulation of DHP that sets foundation for the Bayesian fitting framework for implementing Hubbell’s UNTB in a true multisite setting, i.e., the UNTB-HDP multisite neutral (MSN) model (or the MSN model for short hereafter).

For large local population sizes, assuming a fixed finite-dimensional metacommunity distribution with *S* species present, the local community distribution (π_i_) could be approximated by a Dirichlet distribution (11, 12). Based on this observation, Harris et al. derived their computationally efficient (14) general algorithm for approximating the UNTB. Assuming there is a potentially infinite number of species observed in the local community, the stationary distribution of observing local population *i* can be modeled with a Dirichlet process (DP), i.e.,

(1) $$\bar{\pi_{i}}|M_{i},\bar{\beta }\;∼\;DP(M_{i},\bar{\beta })$$

where $\bar{\beta }=(\beta_{1},\ldots ,\beta_{S})$ is the relative frequency of each species in the metacommunity.

Similarly, a Dirichlet process is also applicable at the metacommunity level, and the metacommunity distribution can be modeled with a stick breaking process, i.e.,

(2) $$\bar{\beta }\;∼\;\mathrm{Stick}(\theta )$$

Since both local community and metacommunity follow Dirichlet processes, the problem can be formulated as a hierarchical Dirichlet process (HDP) in the field of machine learning (14, 42).

Furthermore, the Dirichlet process (DP) is equivalent to the so-termed Chinese restaurant process, from which the Antoniak equation can be inferred (43). The Antoniak equation for the number of types (or species) (*S*) observed following *N* draws from a Dirichlet process with concentration parameter *θ* has the form

(3) $$P(S|\theta ,N)=s(N,S)\theta^{S}\frac{\Gamma (\theta )}{\Gamma (\theta \;+\;N)}$$

where *s*(*N*, *S*) is the unsigned Stirling number of the first kind and Γ(.) is the gamma function.

The full HDP-MSN model (hierarchical Dirichlet process—multisite neutral) is obtained by integrating equations 1, 2, and 3 and the previously mentioned multinominal (MN) distribution of the community samples. To actually fit the MSN model to the data samples from multiple local communities, Harris et al. further developed an efficient Gibbs sampler for the UNTB-HDP approximation (14), a type of Bayesian Markov chain Monte Carlo (MCMC) algorithm. For further information on fitting the MSN model, refer to Harris et al. (14), in which *C* code was provided and is used in this study for testing the goodness of fitting of the HDP-MSN model with the AGM/HGM data sets.

### Neutrality tests with the UNTB-HDP multisite neutral model.

To determine whether an observed species abundance distribution (SAD) data set fits to the UNTB-HDP multisite neutral (MSN) model (i.e., the MSN model for short), Harris et al. proposed a Monte Carlo significance test similar to Etienne’s parametric bootstrap procedure that is supported by a maximum likelihood approach (14, 44). Furthermore, Harris et al. also developed a procedure for testing the neutrality of local community assembly under a fitted possibly nonneutral metacommunity because of the hierarchical nature of the MSN model (14). That is, the MSN model can be used to test the neutrality at both local and metacommunity levels simultaneously, which is an advantage over many other neutral theory models.

For the dual-level tests, samples were generated from an *N* of 2,500 sets of fitted MSN parameters, which were selected from every 10th iteration of the last 25,000 Gibbs samples (50,000 samples in total were simulated and the first 25,000 samples were discarded as burn-in). An *N* of 2,500 is set to compute pseudo *P* values for performing the neutrality test (14). For each observed community sample, there is a corresponding actual log-likelihood *L_0_*. The two additional parameters *θ* and *M* are particular important; *θ* is the median of the fundamental biodiversity numbers computed from 25,000 times of simulations and *M* is the average of the medians of the migration rates (a function of fundamental migration numbers) of local communities in each metacommunity, which is also computed from 25,000 simulations.

To perform a neutrality test at the metacommunity level, *P_M_* is used, which is “the proportion of the simulated neutral samples with their likelihoods not exceeding the observed data likelihood” (14). The computation of *P_M_* is defined as follows: assume *L_M_* is the median of the log-likelihoods of the simulated neutral metacommunity samples, *N_M_* is the number of simulated neutral metacommunity samples having their log-likelihoods meeting *L* ≤ *L_0_* (where *L* is the simulated likelihood and *L_0_* is the actual likelihood as mentioned previously), and then the *P_M_* = *N_M_*/*N* is a pseudo *P* value for testing the neutrality at the metacommunity level. If *P_M_* is ≤0.05, then the chance (probability) that the actual (observed) likelihood exceeding the simulated likelihoods is rather small (≤0.05) and the neutrality null hypothesis should be rejected. Oppositely, if *P_M_* is >0.05, the metacommunity is considered following the MSN model, or the neutral metacommunity null hypothesis could not be rejected (14).

To conduct the neutrality test at the local community level, *P_L_* is used, which is the proportion of the simulated locally neutral samples exceeding the observed data likelihood (14). It is computed similarly to previous step for computing *P_M_*. Assume *L_L_* is the median of the log-likelihoods of the simulated local community samples, *N_L_* is the number of simulated local community samples with their likelihoods not exceeding the *L_0_*, and then *P_L_* = *N_L_*/*N* is a pseudo *P* value for testing the neutrality at the local community level. If *P_L_* is >0.05, the local community is considered following the MSN model, or the neutrality null hypothesis could not be rejected (14).

### Checking type I and type II errors in MSN neutrality tests.

**Type I error, FDR control, and *P* value thresholds.** The previous neutrality test procedures of Harris et al. used a significance level α of 0.05 that may lead to a type I error, namely, incorrectly reject the true neutrality null hypothesis with a 5% probability (14). When many tests are performed simultaneously (so-termed multiple testing problem), the chance for committing a type I error may be inadvertently raised. The false discovery rate (FDR) control is frequently used to adjust the potential bias. However, the slightly “unorthodox” convention used for testing the neutral theory with the previously mentioned MSN tests made FDR adjustment inapplicable. This is because FDR control can only raise the *P* value for each test and, hence, will lead to higher “passing rates” (strictly speaking, “failure rates” to reject neutrality) of neutrality in terms of the convention (e.g., reference 14). In other words, the application of FDR will actually relax the criterion for passing neutrality tests or make the inference less strict (conservative), an undesirable consequence in testing neutral theory. We believe this somewhat unorthodox convention used to test neutral theory in the existing literature, which makes FDR control impossible, is an issue that should be fixed but is rarely raised in our observation. Another consequence (issue) from this “loose” convention is that it often equates the “failure to reject the neutrality null hypothesis” with “passing the neutrality test” or its synonyms that are frequently loosely used in the literature of neutral theory tests.

In the present study, we stick to the traditional convention (e.g., reference 14) to be consistent with existing literature (14), but we introduce an alternative strategy to deal with the issue associated with the type I error by comparing the “passing rates” under different *P* value thresholds. Formally, the term passing rate should actually be considered the failure rate to reject the neutrality null hypothesis. By setting the *P* value for neutrality tests to different thresholds, we can estimate the probability to incorrectly reject the true neutrality null hypothesis under different levels (thresholds) of uncertainty. The higher the *P* value threshold is set, the probability to incorrectly reject the true neutrality null hypothesis becomes higher and the detection of neutral communities becomes more strict (conservative), which is obviously desirable in testing the neutral theory. In the present study, we examine the dynamic changes of the passing rates of MSN neutrality tests when the *P* value threshold was set to 0.05 to 0.95. This allows us to obtain a range of neutrality rates from the least conservative to most conservative in terms of the reliability (conservative level) for detecting the neutrality. With our alterative strategy, although the FDR control is still not applicable, the potential confusion from the loose usage of passing rates become an moot issue since the passing rates are now associated with different levels of reliability (conservative levels). For example, by setting the *P* value threshold to 0.95, we can claim that the passing rate of neutrality tests (or failure rate to reject neutrality null hypothesis) is estimated with extreme caution (being extremely reliable).

**Type II error and Hammal et al. framework for detecting alternative nonneutral processes.** Like any statistical hypothesis tests, testing the neutral theory model, such as the MSN model, also involves a type II error—incorrectly not rejecting a false null hypothesis (i.e., obtaining a false-negative finding). This corresponds to a well-known criticism that apparent satisfaction to the neutral theory patterns may not be due to the neutral processes; instead, nonneutral processes may generate the similar or same patterns indistinguishable statistically from what are predicted by the neutral theory model. If this objection to neutral theory is true, then neutral theory and, to a larger extent, the SAD data sets, are of little or no value in discerning the underlying mechanisms of community structures (specifically community assembly and diversity maintenance). To resolve this issue, Hammal et al. developed a framework to determine when SAD data sets and what neutral models can indeed detect nonneutrality (13). They formulated the problem as a power analysis problem for controlling type II error, and their approach is of critical importance for neutral-theoretic studies like ours.

The power of a statistical test refers to the probability that a false null hypothesis (the alternative hypothesis is true) is correctly rejected, which is equal to 1 − *β*, where *β* is type II error rate. In general, the power of a statistical test will depend on three factors, namely, the sample size, statistical significance as measured by the threshold *P* value (hence influenced by type I error), and the effect size that is quantified by the deviation from the null hypothesis. In the Hammal et al. framework (13), they controlled the effect size with the parameter value of the nonneutral models they developed to simulate possible nonneutral processes in the SAD data to be analyzed. They introduced three nonneutral local community models and two nonneutral metacommunity models, of which all are stochastic and similar to the standard neutral model (SNM) but driven by explicit nonneutral forces, such as competitions and unequal species fitness. They demonstrated that the presence of nonneutral processes in SADs that also satisfy the SNM is detectable as long as the sample size is sufficiently large and/or the effect size (amplitude of nonneutral process effect) is sufficiently strong. They concluded that, although the power analysis (calculation) can indeed be rather complex and computationally exceedingly expensive in particular, resolving the issues related to type II error in analyzing SAD patterns with neutral theory models is possible. In other words, by applying their framework to cross-verifying the tests results from SNM, it is possible to cast convincing evidence to either support or reject the findings from a neutral theory model. In the present study, we adapt their framework to check the validity of our findings from applying the MSN model to analyze the AGM data sets.

The power of a statistical test depends on which alternative hypothesis is assumed. The Hammal et al. framework focused on two classes of nonneutral processes, namely, interspecific competition and intrinsic (density independent) fitness differences between species (13). The former promotes species coexistence and the latter represent the niche differentiations—the mean environmental factors in a particular habitat should favor one species over another. They represent opposite ends of a spectrum of possible nonneutral processes (which could potentially be of infinite varieties). On the one end, the symmetric interspecific competition is likely to generate equal abundances among species (hardly discernible from neutrally generated equal abundances); on the other end, the intrinsic fitness differences tend to generate heterogeneous abundances. Specifically, they introduced two competition models, namely, HL (a multispecies stochastic Lotka-Volterra model similar to one studied by Haegeman and Loreau) and PC (density-dependent dynamics model similar to one studied by Pigolotti and Cencini) (45, 46), and one intrinsic fitness (IF) model that assumes the fecundity of each species is a random variable following a gamma distribution. Furthermore, they introduced two nonneutral metacommunity models, namely, a LOGS model described by a log-series distribution and EVEN model in which all species have equal abundances. Specific forms of these 3 nonneutral local and 2 metacommunity models are referred to in Hammal et al. (13). When linked to the LOGS metacommunity model, each of the three local community models should be equivalent to the SNM model when the local dynamics are neutral (the control parameter is set to zero). When the dynamics turns to be more nonneutral (by increasing the control parameter) and the deviations from the SNM (the effect size) become stronger, it is expected that the power of the test for the neutral null hypothesis will rise.

We slightly revised the Hammal et al. procedure for calculating the power of neutrality tests to achieve our objective to cross-verify the findings from the MSN-based neutrality tests (13). Our steps are detailed as follows:

(i) We randomly chose certain numbers of metacommunities from each of the 3 investigation scales (microbial metacommunity or animal species, microbial landscape or animal class, and microbial global landscape or animal kingdom), namely, 10 metacommunity samples from 10 selected animal species (1 from each species), 10 metacommunities from 10 animal classes (1 from each class), 1 metacommunity sample from animal kingdom (the whole data sets we collected), and 1 metacommunity sample from AGM data sets representing the humans.

(ii) Each metacommunity consists of some local communities. We simulated local community dynamics with PC and IF models using the same parameter values as observed local communities of AGM samples (described by the actual OTU tables) (13, 45, 46). The parameters used to drive simulations include the total number of individuals (*J*), species richness (*S*), immigration rate (*M*), and fundamental biodiversity number (*θ*), which are estimated by the MSN model. The generated local communities are assembled as metacommunity.

(iii) Fitting the MSN model with the simulated metacommunity samples from the previous step.

(iv) Repeat the steps ii and iii 60 times and obtain 60 *P* values. If a *P* value is <0.05, the neutral null hypothesis is rejected, i.e., the alternative nonneutral process is significant. We consider 50 repetitions as a big sample and 60 (> 50) should be sufficient for calculating *P* value.

(v) Compute the power value with the 60 *P* values from step iv. The power of the test is the proportion of nonneutral data sets (generated by PC or IF model simulations) for which the test of nonneutral effect was significant (i.e., the neutral null hypothesis is rejected and the alternative nonneutral process simulated by PC or IF models is accepted).

In summary, the small power value indicates that there is no no-neutral process or the nonneutral process is not sufficiently strong in the metacommunity. Alternatively, the large power indicates that there is a sufficiently strong nonneutral process in the community. Finally, we compare the power test finding (conclusion) with the finding from MSN testing. If both findings are consistent, we conclude that the MSN testing results are reasonably reliable and that the risk of incurring a type II error is tolerable; if not, we conclude that MSN testing results should be questioned. As mentioned previously, the computational load of the Hammal et al. framework is excessive (13); the 60 repetitions (simulations) mentioned previously for calculating the power value means that fitting the MSN model has to be repeated 60 times, i.e., 60 times increase of computational load for each selected sample. It may take us years to complete the computation had we applied the procedure to all 4,903 samples of the AGM data sets. We believe that the randomly chosen metacommunity samples are sufficient to cross-verify the findings from the MSN testing, which is still the primary methodology of this study.

### Computational codes.

We used the computational codes originally provided by Harris et al. for fitting the HDP-MSN model (14). For practical computational limitations, we excluded very small number of insect or mammal samples, with over 30,000 reads from the computation. Even with this sampling scheme, the computation work took nearly 3 months in our server with 512 gigabites of memory space and dual central processing units (CPUs). Nevertheless, we believe that the limitation of extremely large sample sizes should have little influence on the results. We used the computational codes originally developed by Hammal et al. for power analysis (13). Coincidently, Hammal et al. also found that the power calculation would be computationally infeasible for samples with individuals (16S rRNA reads in our case) exceeding approximately 30,000. In fact, completely checking each SAD-MSN data set with the Hammal et al. approach will be hundreds (thousands) of times of computational load increases because each of the hundreds (thousands) of simulations must be tested with the MSN model again (13). With our current computational facility, complete checking of the 4,903 AGM samples would take years finish. For this practical reason, we performed power analysis for selected samples as explained in the previous subsection.

### Data availability.

Table S1 (an Excel table) presents detailed sample information including the data accession numbers. The data were already available in the public domain and no ethic or administrative approval is applicable. The computational codes are available from Harris et al. (14).
