# Supplementary material for: Cross-Scale Analyses of Animal and Human Gut Microbiome Assemblies from Metacommunity to Global Landscape
Source: mSystems. 2021 Jul 6;6(4):e00633-21. doi: 10.1128/mSystems.00633-21 (PMC8407200; doi:10.1128/mSystems.00633-21)
Supplement: TABLE S2 [file msystems.00633-21-st002.pdf]

Table S2. The results of fitting the MSN (multi-site neutral) model at the host species-level or microbial metacommunity scale: the AGM samples from a single animal species were treated as a meta-community, and a total of 274 meta-communities (corresponding to 274 animal species) were tested against the MSN model.

Classes	Species	No. of Samples	L_O	θ	M-value	m	Metacommunity				Local community			
							L_M	N_M	N	p_M	L_L	N_L	N	p_L
Chromadorea	Caenorhabditis_briggsae	5	-1903.792	23.584	6115.352	0.0569	-1013.550	0	2500	0.000	-254.030	0	2500	0.000
	Caenorhabditis_remanei	39	-23952.599	1378.138	7.853	0.0002	-24044.377	1411	2500	0.564	-23671.050	821	2500	0.328
	#Passing rate (%)									50				50
Arachnida	Agele_difficilis	5	-7408.597	1059.461	25.640	0.0007	-6550.243	5	2500	0.002	-6667.656	12	2500	0.005
	Agele_labyrinthica	5	-4098.018	700.037	12.925	0.0004	-3918.288	509	2500	0.204	-3965.724	681	2500	0.272
	Araneae	3	-1672.726	791.039	8.556	0.0006	-1518.761	361	2500	0.144	-1557.761	491	2500	0.196
	Artema_atlanta	5	-2464.458	177.164	10.103	0.0002	-2721.574	2297	2500	0.919	-2548.797	1781	2500	0.712
	Dictis_striatipes	5	-9965.705	1306.892	37.190	0.0010	-8684.506	0	2500	0.000	-8828.366	0	2500	0.000
	Hylyphantes_graminicola	5	-6726.821	1228.894	23.444	0.0006	-5995.510	7	2500	0.003	-6128.189	28	2500	0.011
	Nurscia_albofasciata	5	-3816.326	336.186	15.031	0.0004	-4008.710	2039	2500	0.816	-3884.117	1579	2500	0.632
	Pirata_subpiraticus	5	-5527.738	864.407	16.322	0.0003	-5277.102	379	2500	0.152	-5329.552	531	2500	0.212
	Spider	2	-100.550	21.320	0.733	0.0000	-116.810	1665	2500	0.666	-119.426	1728	2500	0.691
	Ummeliata_insecticeps	5	-6299.336	1074.720	21.748	0.0009	-5454.626	0	2500	0.000	-5580.758	4	2500	0.002
	Passing rate (%)									60				60
Malacostraca	Callinectes_sapidus	7	-5373.477	344.864	14.516	0.0005	-5214.159	636	2500	0.254	-5251.601	729	2500	0.292
	Eriocheir_sinensis	4	-4035.219	2276.076	15.265	0.0013	-3414.437	3	2500	0.001	-3466.705	5	2500	0.002
	not_applicable	2	-4857.558	370.429	55.844	0.0002	-4614.889	310	2500	0.124	-4693.312	468	2500	0.187
	Orchestia_stephenseni	2	-3230.574	386.918	28.299	0.0001	-3038.512	332	2500	0.133	-3119.896	621	2500	0.248
	Porcellio_laevis	4	-9034.694	1495.311	58.642	0.0030	-8150.636	0	2500	0.000	-8378.197	11	2500	0.004
	Talitrus_saltator	3	-3340.999	262.977	25.107	0.0001	-3166.278	448	2500	0.179	-3225.563	599	2500	0.240
	Talorchestia_pelecaniformis	3	-8338.420	521.861	57.951	0.0002	-8327.657	1222	2500	0.489	-8186.861	681	2500	0.272
	#Passing rate (%)									71.4				71.4
Insecta	Acanthocinus_aedilis	2	-2328.678	841.499	17.615	0.0008	-1836.414	2	2500	0.001	-1878.723	1	2500	0.000
	Acheta_domesticus	8	-11240.433	621.121	29.210	0.0009	-10535.695	45	2500	0.018	-10597.707	42	2500	0.017
	Acmaeops_septentrionis	2	-3056.393	614.131	34.693	0.0017	-2517.703	2	2500	0.001	-2602.189	3	2500	0.001
	Aleochara_bilineata	4	-391.719	61.443	1.183	0.0002	-320.543	372	2500	0.149	-334.295	461	2500	0.184
	Aleochara_bipustulata	4	-276.038	83.259	0.770	0.0002	-224.264	466	2500	0.186	-227.196	523	2500	0.209
	Anabrus_simplex	46	-15179.798	143.651	12.457	0.0023	-17288.973	2500	2500	1.000	-14287.690	2	2500	0.001
	Anopheles_stephensi	2	-1012.534	357.486	17.057	0.0010	-873.789	168	2500	0.067	-909.267	300	2500	0.120
	Apis_mellifera	231	-75709.632	337.356	7.150	0.0005	-90656.911	2500	2500	1.000	-70363.400	0	2500	0.000
	Azteca_sp	2	-786.316	346.152	7.156	0.0011	-641.345	138	2500	0.055	-661.961	186	2500	0.074
	Bemisia_tabaci	20	-1401.708	8.983	19.412	0.0022	-2998.327	2499	2500	1.000	-1301.031	410	2500	0.164
	Boisea_trivittata	2	-220.726	23.385	1.981	0.0000	-217.769	1197	2500	0.479	-219.259	1206	2500	0.482
	Bombus_bimaculatus	5	-1413.940	138.439	4.887	0.0002	-1356.446	813	2500	0.325	-1382.306	988	2500	0.395
	Bombus_pascuorum	106	-46650.019	695.439	12.558	0.0002	-66290.722	2500	2500	1.000	-50858.281	2500	2500	1.000
	Cephalotes_atratus	3	-650.636	322.468	3.126	0.0009	-514.189	134	2500	0.054	-526.954	155	2500	0.062
	Cephalotes_borgmeieri	2	-182.183	80.924	1.217	0.0004	-140.646	412	2500	0.165	-145.017	469	2500	0.188
	Cephalotes_clypeatus	3	-316.399	167.273	1.150	0.0002	-262.949	488	2500	0.195	-269.162	579	2500	0.232
	Cephalotes_cordatus	3	-519.627	244.491	2.434	0.0008	-406.409	155	2500	0.062	-418.231	198	2500	0.079
	Cephalotes_eduarduli	3	-347.778	172.724	1.469	0.0004	-270.047	302	2500	0.121	-277.724	326	2500	0.130
	Cephalotes_grandinosus	2	-180.157	67.228	1.124	0.0002	-134.227	373	2500	0.149	-137.867	433	2500	0.173
	Cephalotes_maculatus	3	-325.496	153.944	1.219	0.0003	-251.737	310	2500	0.124	-259.979	331	2500	0.132
	Cephalotes_minutus	5	-711.798	370.455	1.617	0.0005	-560.912	146	2500	0.058	-574.965	174	2500	0.070
	Cephalotes_pallens	4	-552.848	283.021	1.580	0.0004	-442.366	188	2500	0.075	-451.291	257	2500	0.103
	Cephalotes_pallidoides	3	-531.391	260.173	2.398	0.0008	-412.903	147	2500	0.059	-419.779	153	2500	0.061

	Cephalotes_placidus	11	-1701.151	890.629	1.722	0.0005	-1347.975	17	2500	0.007	-1368.409	20	2500	0.008
	Cephalotes_rohweri	2	-308.924	129.013	2.107	0.0005	-240.626	278	2500	0.111	-247.709	335	2500	0.134
	Cephalotes_simillimus	2	-277.110	113.033	2.006	0.0006	-219.137	360	2500	0.144	-227.517	413	2500	0.165
	Cephalotes_spinosus	3	-413.778	187.586	1.603	0.0004	-313.148	187	2500	0.075	-320.572	213	2500	0.085
	Cephalotes_targionii	3	-486.734	223.875	2.035	0.0006	-365.214	107	2500	0.043	-374.343	161	2500	0.064
	Cephalotes_umbraculatus	3	-485.544	240.269	2.196	0.0007	-386.281	220	2500	0.088	-396.388	277	2500	0.111
	Cephalotes_varians	34	-7972.271	3946.001	2.363	0.0007	-6523.168	0	2500	0.000	-6605.727	0	2500	0.000
	Choristoneura_fumifera	176	-46232.489	1378.795	3.630	0.0003	-51792.678	2500	2500	1.000	-44514.809	48	2500	0.019
	Cimex_lectularius	4	-199.150	20.035	0.806	0.0001	-203.904	1359	2500	0.544	-207.337	1423	2500	0.569
	Coleoptera	3	-14506.298	3042.877	151.264	0.0081	-12231.512	0	2500	0.000	-12667.046	0	2500	0.000
	Coptotermes_formosanus	12	-28008.596	545.461	125.818	0.0023	-38292.105	2500	2500	1.000	-29225.675	2495	2500	0.998
	Cornitermes_sp.	3	-19027.834	7826.446	78.270	0.0006	-14634.611	0	2500	0.000	-14931.264	0	2500	0.000
	Cubitermes_ugandensis	3	-3787.050	1650.674	20.867	0.0016	-2989.065	0	2500	0.000	-3057.591	0	2500	0.000
	Delia_radicum	4	-93.986	13.568	0.201	0.0000	-69.303	579	2500	0.232	-72.516	571	2500	0.228
	Drosophila_melanogaster	19	-7779.501	1491.742	4.703	0.0002	-7058.372	33	2500	0.013	-7169.868	83	2500	0.033
	Echidnophaga	8	-557.559	7.530	9.300	0.0009	-930.930	2452	2500	0.981	-526.765	778	2500	0.311
	Formicidae	2	-1361.820	528.088	14.742	0.0009	-1248.274	409	2500	0.164	-1286.547	644	2500	0.258
	Gromphadorhina_portentosa	2	-5427.821	201.750	442.610	0.0079	-5570.759	1843	2500	0.737	-5349.884	642	2500	0.257
	Lucilia_sericata	2	-649.514	180.379	5.256	0.0002	-629.520	1028	2500	0.411	-643.524	1173	2500	0.469
	Lymantria_dispar	9	-6124.827	3589.778	11.244	0.0030	-5052.574	1	2500	0.000	-5133.041	0	2500	0.000
	Manduca_quinquemaculata	7	-7514.000	3202.455	19.783	0.0008	-6440.852	1	2500	0.000	-6604.463	3	2500	0.001
	Manduca_sexta	2	-460.239	173.331	2.048	0.0000	-373.385	344	2500	0.138	-384.501	395	2500	0.158
	Mantis_religiosa	2	-491.701	252.571	3.013	0.0001	-463.096	872	2500	0.349	-471.698	1020	2500	0.408
	Microcerotermes_parvus	3	-10829.835	4625.309	40.882	0.0004	-8422.173	0	2500	0.000	-8583.619	0	2500	0.000
	Musca_domestica	15	-3261.433	22.863	8.770	0.0004	-3331.885	1446	2500	0.578	-3204.500	823	2500	0.329
	Nasutitermes_corniger	3	-6804.373	3288.272	40.960	0.0026	-5376.676	0	2500	0.000	-5495.082	0	2500	0.000
	Neocapritermes_taracua	3	-6686.489	2959.501	31.616	0.0011	-5185.068	0	2500	0.000	-5296.201	0	2500	0.000
	Odonata	2	-1108.837	253.633	13.464	0.0019	-992.800	273	2500	0.109	-1028.666	458	2500	0.183
	Odontotermes_formosanus	12	-46275.174	615.489	271.795	0.0055	-52317.257	2500	2500	1.000	-45386.664	56	2500	0.022
	Ophiotermes_sp.	3	-5312.750	2395.304	30.667	0.0020	-4192.400	0	2500	0.000	-4293.986	0	2500	0.000
	Promirotermes_sp.	3	-6541.175	2821.676	29.276	0.0008	-5059.175	0	2500	0.000	-5148.122	0	2500	0.000
	Pyrrhocoroidea	16	-4390.667	2124.771	2.414	0.0002	-3517.522	0	2500	0.000	-3561.926	2	2500	0.001
	Rhagium_mordax	2	-4947.855	1146.712	46.281	0.0013	-3877.566	0	2500	0.000	-4012.303	0	2500	0.000
	Subpsaltria_yangi	37	-52886.029	1268.154	45.490	0.0009	-79286.301	2500	2500	1.000	-57572.006	2500	2500	1.000
	Tenebrio_molitor	4	-3836.728	111.381	31.290	0.0006	-3654.594	478	2500	0.191	-3649.500	238	2500	0.095
	Termes_hospes	3	-8529.971	3605.315	36.205	0.0007	-6542.098	0	2500	0.000	-6682.769	0	2500	0.000
	Trinervitermes_sp.	3	-2564.674	1194.085	14.269	0.0021	-1989.654	0	2500	0.000	-2038.395	0	2500	0.000
	Trybliographa_rapae	4	-568.212	75.949	2.362	0.0005	-480.431	293	2500	0.117	-496.073	385	2500	0.154
	Passing rate (%)									65.6				60.6
Chondrichthyes	*Carcharhinus_leucas*	9	-12925.714	3375.831	21.475	0.0004	-11690.034	5	2500	0.002	-11928.748	14	2500	0.006
	Carcharhinus_plumbeus	12	-11094.072	1469.066	21.713	0.0012	-10244.964	19	2500	0.008	-10390.335	36	2500	0.014
	Galeocerdo_cuvier	4	-1426.352	61.344	7.439	0.0002	-1393.327	990	2500	0.396	-1385.245	868	2500	0.347
	Leucoraja_ocellata	2	-5168.096	716.169	91.207	0.0022	-4775.815	55	2500	0.022	-4943.471	266	2500	0.106
	Negaprion_brevirostris	5	-2379.134	201.916	11.594	0.0004	-2347.793	1040	2500	0.416	-2352.605	1049	2500	0.420
	#Passing rate (%)									40				60
Actinopteri	*Astyanax_paranae*	9	-12248.045	456.459	124.644	0.0196	-14901.852	2500	2500	1.000	-12118.026	719	2500	0.288
	Aulonocranus_dewindti	5	-10516.821	1996.946	48.057	0.0027	-8669.301	0	2500	0.000	-8933.019	0	2500	0.000
	Carassius_auratus	5	-1982.982	438.183	2.769	0.0000	-1538.799	9	2500	0.004	-1570.390	15	2500	0.006
	Coreius_guichenoti	30	-10119.722	579.103	3.736	0.0002	-8763.351	0	2500	0.000	-8786.530	0	2500	0.000
	Ctenochromis_horei	4	-9670.509	1011.052	75.541	0.0043	-8218.209	0	2500	0.000	-8421.362	0	2500	0.000
	Ctenopharyngodon_idella	108	-81653.167	247.606	24.483	0.0006	-109679.939	2500	2500	1.000	-84232.364	2499	2500	1.000
	Cyprichromis_coloratus	3	-6570.274	651.684	74.276	0.0042	-5843.273	0	2500	0.000	-5914.633	0	2500	0.000

	Eretmodus_cyanostictus	5	-11020.910	1322.302	57.802	0.0033	-9389.344	0	2500	0.000	-9592.796	0	2500	0.000
	Gthochromis_pfefferi	2	-4655.112	456.339	92.104	0.0049	-3952.028	0	2500	0.000	-4076.744	0	2500	0.000
	Huso_dauricus	5	-1217.411	64.039	6.087	0.0001	-1567.705	2472	2500	0.989	-1361.780	2251	2500	0.900
	Interochromis_loocki	5	-23708.820	1940.774	156.366	0.0056	-21506.119	0	2500	0.000	-21475.276	0	2500	0.000
	Julidochromis_ornatus	5	-21688.081	1040.544	220.830	0.0064	-22736.638	2480	2500	0.992	-20640.170	2	2500	0.001
	Konia_dikume	3	-6333.121	337.159	145.573	0.0072	-6288.999	1031	2500	0.412	-6021.591	39	2500	0.016
	Konia_eisentrauti	4	-3164.846	83.192	107.325	0.0062	-3652.262	2479	2500	0.992	-3110.781	732	2500	0.293
	Lamprologus_lemairii	4	-2103.000	213.668	12.967	0.0018	-1877.672	112	2500	0.045	-1897.300	105	2500	0.042
	Lepidiolamprologus_attenuatus	5	-5205.807	331.274	33.644	0.0015	-5050.288	555	2500	0.222	-4973.328	254	2500	0.102
	Lepidiolamprologus_elongatus	3	-3320.857	257.116	35.376	0.0017	-3007.324	74	2500	0.030	-3073.649	109	2500	0.044
	Myaka_myaka	4	-5792.387	379.621	64.610	0.0049	-5508.395	229	2500	0.092	-5426.726	42	2500	0.017
	Neolamprologus_fasciatus	5	-4377.769	372.476	20.390	0.0012	-4085.491	202	2500	0.081	-4109.272	180	2500	0.072
	Neolamprologus_pulcher	5	-17571.363	1290.380	125.762	0.0051	-16797.902	26	2500	0.010	-16439.385	0	2500	0.000
	Neolamprologus_savoryi	4	-12006.378	954.710	106.313	0.0050	-10995.423	0	2500	0.000	-10934.250	0	2500	0.000
	Oncorhynchus_mykiss	110	-136897.673	5598.142	24.380	0.0041	-130312.081	0	2500	0.000	-124911.448	0	2500	0.000
	Ophthalmotilapia_ventralis	3	-13641.592	1073.349	185.766	0.0062	-12101.649	0	2500	0.000	-12256.123	0	2500	0.000
	Oreochromis_niloticus	27	-43784.659	5073.673	26.013	0.0011	-38377.480	0	2500	0.000	-38754.979	0	2500	0.000
	Perca_fluviatilis	145	-28452.799	7632.169	1.525	0.0003	-23049.199	0	2500	0.000	-23395.699	0	2500	0.000
	Plecodus_straeleni	3	-2198.606	144.227	23.868	0.0007	-2389.060	2239	2500	0.896	-2226.091	1508	2500	0.603
	Poecilia_reticulata	48	-17506.133	1074.079	12.359	0.0033	-17426.462	1039	2500	0.416	-16992.602	206	2500	0.082
	Pomacentrus_chrysurus	3	-241.865	8.728	9.540	0.0022	-299.545	2087	2500	0.835	-240.243	1203	2500	0.481
	Pomacentrus_moluccensis	3	-1164.851	432.569	6.842	0.0008	-1007.769	202	2500	0.081	-1043.587	290	2500	0.116
	Pomacentrus_unknown	4	-458.099	21.984	1.992	0.0001	-437.010	977	2500	0.391	-430.850	799	2500	0.320
	Prionotus_carolinus	2	-10453.072	4284.542	110.182	0.0022	-9129.666	1	2500	0.000	-9382.966	1	2500	0.000
	Pungu_maclareni	5	-4747.272	140.720	62.766	0.0041	-5220.805	2424	2500	0.970	-4627.809	503	2500	0.201
	Salmo_trutta	61	-49602.480	1188.374	40.652	0.0048	-87907.624	2500	2500	1.000	-50240.811	2059	2500	0.824
	Sarotherodon_caroli	4	-4934.135	145.649	124.804	0.0072	-5547.827	2489	2500	0.996	-4735.445	176	2500	0.070
	Sarotherodon_linnellii	5	-5540.943	142.200	117.804	0.0075	-6622.420	2500	2500	1.000	-5417.730	488	2500	0.195
	Sarotherodon_lohbergeri	3	-4442.996	453.309	44.580	0.0025	-3942.582	10	2500	0.004	-4017.712	7	2500	0.003
	Sarotherodon_steinbachi	5	-16381.395	1828.899	94.904	0.0049	-14662.698	0	2500	0.000	-14768.350	0	2500	0.000
	Siganus_fuscescens	11	-4277.092	76.909	31.921	0.0049	-5547.370	2500	2500	1.000	-4069.548	198	2500	0.079
	Simochromis_babaulti	2	-4665.531	341.679	128.264	0.0067	-4048.882	0	2500	0.000	-4155.250	0	2500	0.000
	Siniperca_chuatsi	41	-51942.020	1622.108	29.491	0.0015	-57283.625	2500	2500	1.000	-51961.936	1278	2500	0.511
	Stomatepia_mariae	4	-3437.701	129.073	56.440	0.0030	-3473.689	1448	2500	0.579	-3316.118	415	2500	0.166
	Stomatepia_pindu	5	-4460.753	101.484	69.229	0.0043	-4437.990	1153	2500	0.461	-4265.818	151	2500	0.060
	Variabilichromis_moorii	2	-9445.104	777.532	277.412	0.0090	-8476.178	0	2500	0.000	-8662.511	0	2500	0.000
	Xenotilapia_melanogenys	2	-2375.668	184.994	49.999	0.0021	-2350.636	1080	2500	0.432	-2318.546	785	2500	0.314
	Xenotilapia_spiloptera	5	-14336.263	1240.326	90.594	0.0051	-12961.080	0	2500	0.000	-12977.407	0	2500	0.000
	#Passing rate (%)									51.1				44.4
	Dendropsophus_minutus	2	-603.252	44.304	23.397	0.0075	-581.965	937	2500	0.375	-568.196	637	2500	0.255
Amphibia	*Fejervarya_limnocharis*	6	-10751.177	6789.271	39.151	0.0074	-8941.362	0	2500	0.000	-9104.423	0	2500	0.000
	Scix_fuscovarius	13	-20619.041	694.202	127.130	0.0124	-24733.771	2500	2500	1.000	-20492.026	850	2500	0.340
	#Passing rate (%)									66.7				66.7
Sauropsida	*Chelonia_mydas*	24	-29889.049	13120.572	9.661	0.0002	-23297.323	0	2500	0.000	-23579.473	0	2500	0.000
	Lampropeltis_triangulum	2	-1294.627	622.988	9.480	0.0004	-1104.020	103	2500	0.041	-1127.140	199	2500	0.080
	Liolaemus_parvus	88	-186500.243	3767.292	52.280	0.0021	-221616.012	2500	2500	1.000	-188227.283	2255	2500	0.902
	Liolaemus_ruibali	96	-189421.024	4359.188	41.154	0.0018	-208365.593	2500	2500	1.000	-189344.183	1191	2500	0.476
	Pantherophis_obsoletus	4	-2577.903	1418.027	9.363	0.0006	-2183.017	25	2500	0.010	-2220.996	52	2500	0.021
	Phymaturus_williamsi	57	-144675.474	3037.905	75.248	0.0025	-179072.361	2500	2500	1.000	-146961.792	2454	2500	0.982
	Pogona_barbata	3	-5756.488	2894.730	30.218	0.0010	-4798.758	0	2500	0.000	-4891.243	0	2500	0.000
	Sauromalus_ater	2	-1948.046	652.552	15.215	0.0005	-1622.410	36	2500	0.014	-1674.120	51	2500	0.020
	Varanus_komodoensis	24	-24908.529	541.395	21.780	0.0005	-27525.895	2500	2500	1.000	-24776.405	953	2500	0.381

	Varanus_olivaceus	4	-10197.332	412.694	191.597	0.0035	-11998.810	2500	2500	1.000	-10403.983	2079	2500	0.832
	Passing rate (%)									50				60
Aves	*Anser_cygnoides*	18	-54815.204	7662.009	46.117	0.0019	-45426.501	0	2500	0.000	-46208.573	0	2500	0.000
	Bubo_bubo	3	-1834.299	53.901	168.357	0.0114	-2085.401	2347	2500	0.939	-1782.819	644	2500	0.258
	Buteo_jamaicensis	3	-3697.315	1099.744	25.162	0.0009	-3198.139	6	2500	0.002	-3285.812	28	2500	0.011
	Chiroxiphia_lanceolata	9	-19947.448	937.590	35.894	0.0003	-19657.065	689	2500	0.276	-18450.940	0	2500	0.000
	Chroicocephalus_ridibundus	86	-68334.318	1614.235	22.981	0.0010	-109775.098	2500	2500	1.000	-72749.620	2500	2500	1.000
	Columbi_passerina	39	-73639.271	2650.745	28.665	0.0004	-74416.529	1924	2500	0.770	-69617.038	0	2500	0.000
	Columbi_talpacoti	24	-24503.741	1024.912	16.209	0.0004	-24432.553	1120	2500	0.448	-23316.109	28	2500	0.011
	Duck	3	-3531.042	1573.842	20.166	0.0012	-2938.191	1	2500	0.000	-3007.323	5	2500	0.002
	Eudyptula_minor	2	-855.721	371.464	9.324	0.0040	-665.099	35	2500	0.014	-683.769	43	2500	0.017
	Haliaeetus_leucocephalus	2	-1935.166	885.937	14.861	0.0005	-1649.758	80	2500	0.032	-1688.969	109	2500	0.044
	Hirundo_rustica	17	-21355.373	1804.645	23.538	0.0013	-20530.066	123	2500	0.049	-20250.764	18	2500	0.007
	Melopsittacus_undulatus	14	-6903.044	129.163	13.571	0.0007	-7531.831	2424	2500	0.970	-6624.538	279	2500	0.112
	Nymphicus_hollandicus	6	-4210.213	584.181	10.148	0.0003	-3609.458	2	2500	0.001	-3689.638	17	2500	0.007
	Peafowl	3	-2167.843	972.238	11.978	0.0008	-1858.477	43	2500	0.017	-1903.162	92	2500	0.037
	Penguin	4	-1463.357	752.334	4.729	0.0003	-1176.187	37	2500	0.015	-1196.966	52	2500	0.021
	Phoenicopterus_chilensis	2	-1286.742	27.790	2084.660	0.1528	-1098.612	117	2500	0.047	-1391.199	2500	2500	1.000
	Puffinus_tenuirostris	4	-824.598	443.328	2.642	0.0006	-682.124	170	2500	0.068	-695.617	217	2500	0.087
	Ramphocelus_carbo	8	-6609.274	656.459	13.237	0.0004	-6114.571	81	2500	0.032	-6161.177	82	2500	0.033
	Saltator_maximus	11	-9616.925	910.145	14.072	0.0005	-8806.613	9	2500	0.004	-8825.714	10	2500	0.004
	Sarcoramphus_papa	2	-1602.316	750.461	11.632	0.0003	-1350.533	68	2500	0.027	-1377.965	124	2500	0.050
	Serinus_caria	6	-3961.490	381.945	11.093	0.0004	-3593.688	82	2500	0.033	-3579.267	71	2500	0.028
	Strigops_habroptilus	28	-7902.316	3815.365	3.573	0.0013	-6477.441	0	2500	0.000	-6584.832	1	2500	0.000
	Struthio_camelus	167	-259559.842	2280.352	35.852	0.0025	-275626.318	2500	2500	1.000	-243711.421	0	2500	0.000
	Tangara_palmarum	3	-6451.989	436.968	40.242	0.0003	-5829.965	14	2500	0.006	-5771.360	0	2500	0.000
	Thraupis_gaucocolpa	8	-15505.748	864.002	29.141	0.0002	-14904.441	194	2500	0.078	-14419.783	7	2500	0.003
	Turdus_flavipes	6	-5078.193	672.482	14.080	0.0005	-4487.083	11	2500	0.004	-4572.876	20	2500	0.008
	Turdus_olivater	6	-4255.106	467.759	12.092	0.0003	-3852.124	66	2500	0.026	-3915.021	131	2500	0.052
	Urocolius_macrourus	3	-1171.253	584.601	7.102	0.0003	-963.031	78	2500	0.031	-993.036	165	2500	0.066
	Zonotrichia_capensis	6	-10492.714	942.705	33.378	0.0011	-9376.971	2	2500	0.001	-9383.615	0	2500	0.000
	#Passing rate (%)									31.0				24.1
Mammalia	*Acinonyx_jubatus*	67	-40297.783	658.109	8.442	0.0005	-38539.822	24	2500	0.010	-35297.133	0	2500	0.000
	Aepyceros_melampus	6	-49299.335	3822.700	158.301	0.0012	-42756.479	0	2500	0.000	-43408.075	0	2500	0.000
	Ailuropoda_melanoleuca	11	-4891.561	2407.390	5.821	0.0005	-4063.482	2	2500	0.001	-4134.376	3	2500	0.001
	Ailurus_fulgens	5	-2510.350	356.353	5.689	0.0001	-2214.982	127	2500	0.051	-2265.378	181	2500	0.072
	Alces_alces	7	-18577.783	7087.036	39.840	0.0010	-16071.654	0	2500	0.000	-16361.222	0	2500	0.000
	Antidorcas_marsupialis	8	-70140.362	3628.581	205.800	0.0017	-63545.517	0	2500	0.000	-64324.900	0	2500	0.000
	Arctictis_binturong	2	-925.975	417.172	6.066	0.0002	-767.412	159	2500	0.064	-785.374	226	2500	0.090
	Arctocephalus_australis	6	-6000.796	175.842	22.868	0.0002	-6252.671	2013	2500	0.805	-5711.914	221	2500	0.088
	Arctocephalus_tropicalis	4	-4060.864	143.014	22.820	0.0002	-3967.251	842	2500	0.337	-3819.113	181	2500	0.072
	Armadillo	2	-1471.993	553.236	10.327	0.0003	-1231.545	77	2500	0.031	-1262.609	98	2500	0.039
	Bison_bison	7	-24246.873	15197.688	79.678	0.0110	-19682.025	0	2500	0.000	-20071.721	0	2500	0.000
	Bos_indicus	9	-10194.779	6241.378	23.627	0.0079	-8320.283	0	2500	0.000	-8475.514	0	2500	0.000
	Bos_taurus	105	-329138.147	36670.364	59.313	0.0054	-289728.898	0	2500	0.000	-294876.671	0	2500	0.000
	Bubalus_bubalis	2	-2588.704	1386.158	39.361	0.0124	-2054.234	0	2500	0.000	-2126.455	1	2500	0.000
	Canis_latrans	80	-135006.091	3420.734	22.524	0.0003	-135981.377	1878	2500	0.751	-132238.188	39	2500	0.016
	Canis_mesomelas	50	-21292.313	1239.574	5.033	0.0006	-18542.309	0	2500	0.000	-18355.354	0	2500	0.000
	Capreolus_capreolus	2	-2314.409	1169.569	33.649	0.0110	-1819.177	0	2500	0.000	-1880.530	1	2500	0.000
	Castor_cadensis	3	-1920.368	342.153	14.869	0.0011	-1571.907	5	2500	0.002	-1633.556	23	2500	0.009
	Cavia_porcellus	7	-27473.487	9140.923	73.060	0.0023	-23105.741	0	2500	0.000	-23678.125	0	2500	0.000
	Cebus_capucinus	49	-7760.039	3410.876	1.093	0.0001	-6259.923	0	2500	0.000	-6327.927	0	2500	0.000

	Ceratotherium_simum	7	-38879.162	3185.253	106.482	0.0012	-34565.415	0	2500	0.000	-35621.293	0	2500	0.000
	Cercocebus_atys	2	-8699.388	1259.199	157.893	0.0056	-7408.382	0	2500	0.000	-7709.474	0	2500	0.000
	Cervus_elaphus	12	-34731.262	12155.918	49.974	0.0020	-29544.464	0	2500	0.000	-30205.338	0	2500	0.000
	Chaetophractus_vellerosus	3	-4506.644	197.343	74.437	0.0032	-4253.002	244	2500	0.098	-4286.205	122	2500	0.049
	Chinchilla_brevicaudataus	2	-3287.966	494.261	45.622	0.0010	-2851.086	3	2500	0.001	-2971.251	27	2500	0.011
	Choloepus_didactylus	3	-5939.303	379.887	73.000	0.0026	-5558.603	93	2500	0.037	-5575.702	52	2500	0.021
	Choloepus_hoffmanni	4	-5954.530	198.839	80.204	0.0034	-5855.557	876	2500	0.350	-5755.250	237	2500	0.095
	Choloepus	2	-4337.903	117.954	436.250	0.0053	-4320.125	1166	2500	0.466	-4203.156	229	2500	0.092
	Colobus_guereza	4	-10197.024	376.156	113.408	0.0027	-9876.063	391	2500	0.156	-9698.447	22	2500	0.009
	Connochaetes_taurinus	7	-57373.246	4769.275	161.714	0.0013	-49563.624	0	2500	0.000	-50845.027	0	2500	0.000
	Dasypus_novemcinctus	12	-24197.040	670.450	75.195	0.0039	-25868.840	2500	2500	1.000	-23540.139	94	2500	0.038
	Diceros_bicornis	4	-27590.855	1818.317	166.507	0.0013	-24455.178	0	2500	0.000	-25044.100	0	2500	0.000
	Didelphis_virginiana	4	-5049.090	2144.199	21.124	0.0012	-4171.751	0	2500	0.000	-4279.737	0	2500	0.000
	Dipodomys_ordii	6	-41248.223	11468.386	115.972	0.0018	-35177.105	0	2500	0.000	-36017.246	0	2500	0.000
	Dolichotis_patagonum	3	-4978.344	1794.135	32.199	0.0016	-4095.262	0	2500	0.000	-4219.565	0	2500	0.000
	Echinops_telfairi	8	-3714.937	1490.517	5.961	0.0003	-3108.725	5	2500	0.002	-3164.350	16	2500	0.006
	Equus_caballus	44	-175452.380	3998.540	136.654	0.0069	-180853.842	2500	2500	1.000	-167357.042	0	2500	0.000
	Erethizon_dorsatum	2	-1028.358	103.716	28.572	0.0038	-869.426	65	2500	0.026	-915.248	130	2500	0.052
	Eubalaena_glacialis	3	-14262.712	391.144	232.533	0.0006	-14911.960	2317	2500	0.927	-13546.238	8	2500	0.003
	Eulemur_mongoz	3	-3531.613	1530.718	19.320	0.0008	-2999.990	8	2500	0.003	-3070.632	27	2500	0.011
	Euphractus_sexcinctus	4	-6017.996	233.764	65.550	0.0030	-5649.560	127	2500	0.051	-5701.527	62	2500	0.025
	Gerbillus_gerbillus	3	-4536.083	124.374	386.491	0.0183	-4914.058	2351	2500	0.940	-4308.773	42	2500	0.017
	Giraffa_camelopardalis	6	-47615.630	3546.084	166.065	0.0015	-42051.467	0	2500	0.000	-42630.373	0	2500	0.000
	Gorilla_gorilla	10	-40700.364	4263.991	75.200	0.0017	-36249.634	0	2500	0.000	-37116.663	0	2500	0.000
	Hipposideros_armiger	18	-13505.681	426.048	22.403	0.0031	-14255.201	2436	2500	0.974	-13185.207	333	2500	0.133
	Homo_sapiens	23	-46469.034	1679.352	35.772	0.0009	-43649.342	0	2500	0.000	-42993.236	0	2500	0.000
	Hydrochoerus_hydrochaeris	2	-9090.523	369.643	626.026	0.0078	-8964.939	804	2500	0.322	-8629.358	9	2500	0.004
	Hystrix_indica	2	-8524.286	312.148	479.766	0.0046	-8176.926	297	2500	0.119	-8144.001	30	2500	0.012
	Kogia_breviceps	9	-22502.096	11523.824	30.036	0.0010	-17847.999	0	2500	0.000	-18113.233	0	2500	0.000
	Kogia_sima	4	-10383.681	5104.455	33.751	0.0009	-8155.970	0	2500	0.000	-8315.915	0	2500	0.000
	Lemur_catta	6	-12202.717	1814.494	37.826	0.0012	-10508.726	0	2500	0.000	-10834.810	0	2500	0.000
	Leontopithecus_rosalia	2	-1941.847	632.863	15.806	0.0005	-1629.890	44	2500	0.018	-1677.984	73	2500	0.029
	Lontra_cadensis	5	-3891.403	1308.044	14.340	0.0007	-3191.615	1	2500	0.000	-3281.776	2	2500	0.001
	Loxodonta	2	-4690.195	174.946	334.234	0.0078	-4651.666	1076	2500	0.430	-4510.816	141	2500	0.056
	Lynx_rufus	38	-73788.149	3361.037	26.424	0.0002	-72261.448	161	2500	0.064	-71608.052	30	2500	0.012
	Macaca_mulatta	6	-27266.714	1536.891	136.942	0.0030	-26292.263	46	2500	0.018	-25764.718	1	2500	0.000
	Macropus_rufus	18	-44425.290	23495.820	30.900	0.0014	-36580.388	0	2500	0.000	-37108.201	0	2500	0.000
	Mandrillus_sphinx	2	-8960.646	908.935	167.279	0.0036	-8022.081	1	2500	0.000	-8235.045	0	2500	0.000
	Manis_pentadactyla	2	-1499.856	158.729	20.754	0.0012	-1366.897	255	2500	0.102	-1407.886	398	2500	0.159
	Megaptera_novaeangliae	3	-12988.123	826.606	109.772	0.0006	-11680.300	1	2500	0.000	-11786.346	0	2500	0.000
	Mesocricetus_auratus	3	-9721.971	440.122	255.678	0.0078	-9862.850	1755	2500	0.702	-9299.076	38	2500	0.015
	Microtus_californicus	2	-8304.907	4344.717	65.071	0.0008	-7256.472	1	2500	0.000	-7406.702	2	2500	0.001
	Mus_musculus	12	-21348.951	1868.631	33.184	0.0015	-18568.720	0	2500	0.000	-19064.538	0	2500	0.000
	Mustela_putorius	3	-2305.226	932.830	12.807	0.0005	-2101.303	245	2500	0.098	-2145.038	384	2500	0.154
	Myrmecophaga_tridactyla	35	-46063.663	1427.022	24.799	0.0013	-44693.456	65	2500	0.026	-43498.056	0	2500	0.000
	Ochotona_curzoniae	41	-47902.498	21625.569	10.185	0.0004	-37963.569	0	2500	0.000	-38408.637	0	2500	0.000
	Odocoileus_virginianus	8	-51268.828	6402.659	123.062	0.0016	-45418.157	0	2500	0.000	-46673.114	0	2500	0.000
	Onychomys_leucogaster	2	-6544.965	2516.117	43.064	0.0002	-5564.428	2	2500	0.001	-5687.168	2	2500	0.001
	Orycteropus_afer	14	-19383.275	692.025	32.067	0.0016	-18278.148	9	2500	0.004	-18185.818	1	2500	0.000
	Oryctolagus_cuniculus	15	-50265.930	6043.039	60.823	0.0016	-43916.843	0	2500	0.000	-44905.968	0	2500	0.000
	Oryctolagus	102	-388563.150	160118.413	42.890	0.0017	-327393.080	1134	1366	0.000	-331568.703	1134	1366	0.000
	Ovis_aries	29	-100712.136	20650.777	77.129	0.0069	-85877.956	0	2500	0.000	-88031.916	0	2500	0.000

	Pan_paniscus	68	-250555.182	3070.209	91.496	0.0018	-284188.960	2500	2247	1.000	-239464.854	253	2247	0.000
	Panthera_leo	2	-964.468	338.328	6.383	0.0002	-796.801	154	2500	0.062	-815.183	181	2500	0.072
	Panthera_tigris	3	-1133.080	510.554	4.367	0.0002	-913.607	95	2500	0.038	-936.051	107	2500	0.043
	Perogthus_parvus	6	-23039.645	1790.619	68.013	0.0003	-21231.155	1	2500	0.000	-21800.778	8	2500	0.003
	Piliocolobus_badius	2	-9151.116	1332.551	162.169	0.0055	-7880.202	0	2500	0.000	-8176.083	0	2500	0.000
	Pongo_pygmaeus	3	-8526.785	312.523	179.542	0.0026	-8448.467	1011	2500	0.404	-8186.818	72	2500	0.029
	Porcupine	3	-5609.011	1993.945	31.888	0.0009	-4649.544	0	2500	0.000	-4781.658	1	2500	0.000
	Priodontes_maximus	4	-10026.444	3493.988	50.041	0.0030	-8346.036	0	2500	0.000	-8561.543	0	2500	0.000
	Proteles_cristata	2	-1412.123	33.633	430.748	0.0235	-1361.373	836	2500	0.334	-1378.899	599	2500	0.240
	Puma_concolor	24	-56550.423	2185.276	36.439	0.0004	-55842.824	472	2500	0.189	-55279.860	112	2500	0.045
	Rangifer_tarandus	8	-39075.736	1934.191	122.614	0.0024	-35545.036	0	2500	0.000	-36582.790	0	2500	0.000
	Rattus_norvegicus	5	-11699.502	1814.285	47.847	0.0018	-10042.661	0	2500	0.000	-10358.025	0	2500	0.000
	Rhinolophus_ferrumequinum	18	-12007.052	364.878	17.078	0.0019	-12198.221	1779	2500	0.712	-11519.872	130	2500	0.052
	Saguinus_oedipus	2	-1689.476	681.275	12.307	0.0004	-1431.968	69	2500	0.028	-1466.688	106	2500	0.042
	Simia_paniscus	4	-3635.100	1891.971	14.850	0.0012	-3041.215	5	2500	0.002	-3108.911	8	2500	0.003
	Suricata_suricatta	2	-1648.185	421.826	14.604	0.0005	-1403.780	63	2500	0.025	-1451.232	121	2500	0.048
	Sus_scrofa	2	-2315.313	674.726	23.785	0.0011	-1966.831	13	2500	0.005	-2032.701	58	2500	0.023
	Tamandua_tetradactyla	2	-2653.332	251.273	41.782	0.0017	-2391.139	50	2500	0.020	-2455.483	113	2500	0.045
	Tamias_minimus	6	-16198.539	4867.053	38.970	0.0006	-14177.680	0	2500	0.000	-14484.705	0	2500	0.000
	Tapirus_indicus	3	-15167.719	963.757	174.678	0.0023	-14021.730	0	2500	0.000	-14218.225	0	2500	0.000
	Taxidea_taxus	2	-1255.619	60.433	16.997	0.0001	-1255.295	1249	2500	0.500	-1244.928	1130	2500	0.452
	Tolypeutes_matacus	3	-4734.529	207.973	80.098	0.0032	-4523.036	355	2500	0.142	-4495.840	113	2500	0.045
	Tupaia_belangeri	4	-2104.535	998.445	6.955	0.0004	-1789.475	52	2500	0.021	-1829.311	107	2500	0.043
	Tursiops_truncatus	41	-10876.961	5702.035	3.154	0.0011	-9314.351	0	2500	0.000	-9442.420	0	2500	0.000
	Ursus_arctos	2	-1139.913	469.829	7.726	0.0002	-919.668	67	2500	0.027	-942.806	85	2500	0.034
	Varecia_rubra	3	-4081.501	1494.750	21.945	0.0007	-3385.497	0	2500	0.000	-3463.964	5	2500	0.002
	Vespertilio_sinensis	17	-12352.185	411.895	19.302	0.0023	-12605.251	1900	2500	0.760	-11777.572	81	2500	0.032
	Vulpes_lagopus	5	-7207.401	1092.861	26.199	0.0008	-6133.731	0	2500	0.000	-6326.786	1	2500	0.000
	Zaedyus_pichiy	2	-2650.833	295.983	50.619	0.0028	-2385.633	53	2500	0.021	-2476.917	164	2500	0.066
	Zalophus_californianus	13	-11509.865	6034.048	12.655	0.0005	-9535.517	0	2500	0.000	-9688.166	0	2500	0.000
	Passing rate (%)									30.4				15.7
#Passing rate (%) (P-value=0.05)										45.3				37.6
**Mean**			-20036.273	2502.204	85.378	0.003	-19988.854	582.6	2494.9	0.231	-18593.889	295.29	2494.9	0.116
**Std. Err.**			2730.537	620.126	23.841	0.001	2744.906	53.288	4.237	0.021	2525.490	33.012	4.237	0.013

**N*=2500 is the number of Gibb samples selected from 25000 simulated communities (*i.e.,* every tenth iteration of the last 25,000 Gibbs samples), it is chosen to compute the pseudo *p*-value (as explained below) for conducting the neutrality test. $\boldsymbol{L_0}$ is the *actual log-likelihood*. θ is the median of biodiversity parameters computed from 25,000 times of simulations. *M*-value is the average medians of the migration rates of local communities in each meta-community, also computed from 25,000 times of simulations. $\boldsymbol{L_M}$ is the median of the log-likelihoods of the simulated neutral meta-community samples; and $\boldsymbol{N_M}$ is the number of simulated neutral meta-community samples with their likelihoods not exceeding the actual likelihood satisfying (*i.e*., $\boldsymbol{L \leq L_0}$ where $\boldsymbol{L}$ and $\boldsymbol{L_0}$ are the simulated and actual likelihood respectively), $\boldsymbol{P_M}=\boldsymbol{N_M}/\boldsymbol{N}$ is the pseudo *p*-value for testing the neutrality at meta-community level; traditionally, if $\boldsymbol{P_M}>0.05$, the meta-community is considered indistinguishable from pattern predicted by the MSN model. $\boldsymbol{L_L}$ is the median of the log-likelihoods of the simulated local community samples, and $\boldsymbol{N_L}$ is the number of simulated local community samples with their likelihoods not exceeding the L_0, (*i.e*., $\boldsymbol{L \leq L_0}$ where $\boldsymbol{L}$ and $\boldsymbol{L_0}$ are the simulated and actual likelihood respectively), $\boldsymbol{P_L=N_L/N}$, is the pseudo *p*-value for testing the neutrality at the local community level; if $\boldsymbol{P_L}>0.05$, the local community satisfies the neutral model. *See* Harris *et al*. (2017) for more detailed explanations of these fitting parameters. Also *see* legends for Table 2 for additional information on P-value.

**Due to a typo error in Harris *et al*. (2017), the $\boldsymbol{P_M}$-values exhibited here are adjusted as ($\boldsymbol{P_M}=1-\boldsymbol{P_{MS}}$), where $\boldsymbol{P_{MS}}$ is output from their computational program. Similarly, the $\boldsymbol{P_L}$-values are adjusted as ($\boldsymbol{P_L}=1-\boldsymbol{P_{LS}}$), where $\boldsymbol{P_{LS}}$ is output from their computational program. See https://arxiv.org/abs/1410.4038 for the latest update of Harris et al. (2017).
