# Supplementary material for: Cross-Scale Analyses of Animal and Human Gut Microbiome Assemblies from Metacommunity to Global Landscape
Source: mSystems. 2021 Jul 6;6(4):e00633-21. doi: 10.1128/mSystems.00633-21 (PMC8407200; doi:10.1128/mSystems.00633-21)
Supplement: TABLE S5 [file msystems.00633-21-st005.pdf]

Table S5. Fitting the MSN model at host-*class* level (microbiome landscape level): For each animal class, one AGM sample was randomly taken from each animal species within the same class, and all obtained samples were treated as a super-metacommunity to represent for the class, and were fitted to a MSN model for the class. A total of 10 MSN models for 10 super-meta-communities, corresponding to 10 animal classes, were fitted. To increase the robustness of the result, 100 times of repetitions of the process were performed by re-sampling samples from each animal species without replacement. Therefore, a total of 1000 (100x10) MSN models were built at the class level. For the classes of insects and mammals, occasionally, the samples with more than 30,000 reads were excluded due to the limitation of computational program used)*.

Sampling No.		L_O	θ	M	*Metacommunity*				*Local community*			
					L_M	N_M	N	P_M	L_L	N_L	N	P_L
Chromadorea	1	-2050.506	926.918	6.804	-1650.141	7	2500	0.003	-1679.705	23	2500	0.009
	2	-2289.902	1022.118	7.198	-1838.764	7	2500	0.003	-1872.308	9	2500	0.004
	3	-2921.391	1110.427	11.938	-2454.365	6	2500	0.002	-2518.928	14	2500	0.006
	4	-1797.996	886.861	6.321	-1505.408	46	2500	0.018	-1534.333	77	2500	0.031
	5	-2662.252	1198.121	12.836	-2223.062	9	2500	0.004	-2283.487	17	2500	0.007
	6	-1475.366	626.424	4.662	-1200.850	44	2500	0.018	-1224.176	78	2500	0.031
	7	-1704.259	728.764	5.189	-1393.897	35	2500	0.014	-1421.205	54	2500	0.022
	8	-1836.134	756.223	5.691	-1457.209	11	2500	0.004	-1487.260	21	2500	0.008
	9	-1413.028	571.515	5.409	-1117.808	16	2500	0.006	-1142.510	29	2500	0.012
	10	-2942.728	1405.172	12.653	-2474.824	6	2500	0.002	-2538.188	23	2500	0.009
	11	-1895.069	922.894	6.459	-1603.417	57	2500	0.023	-1633.331	100	2500	0.040
	12	-2226.674	1084.402	9.430	-1795.494	7	2500	0.003	-1837.321	10	2500	0.004
	13	-1064.692	512.195	3.499	-897.564	178	2500	0.071	-915.102	209	2500	0.084
	14	-1734.286	857.020	7.298	-1428.844	35	2500	0.014	-1466.243	58	2500	0.023
	15	-1744.993	780.212	7.133	-1418.077	14	2500	0.006	-1445.382	36	2500	0.014
	16	-1912.851	912.872	6.893	-1583.897	23	2500	0.009	-1614.054	46	2500	0.018
	17	-1541.975	647.152	5.432	-1240.124	29	2500	0.012	-1272.624	31	2500	0.012
	18	-1769.755	651.990	5.645	-1408.610	14	2500	0.006	-1436.311	16	2500	0.006
	19	-2974.247	1053.073	12.573	-2480.382	5	2500	0.002	-2546.717	17	2500	0.007
	20	-1424.636	530.677	6.717	-1166.824	34	2500	0.014	-1195.361	56	2500	0.022
	21	-2362.057	796.718	9.692	-1868.311	3	2500	0.001	-1917.075	3	2500	0.001
	22	-1919.426	812.025	7.849	-1558.326	12	2500	0.005	-1601.696	17	2500	0.007
	23	-984.895	400.902	3.305	-816.261	153	2500	0.061	-832.797	197	2500	0.079
	24	-2570.272	1181.735	9.596	-2099.401	4	2500	0.002	-2144.357	12	2500	0.005
	25	-1720.457	830.673	6.242	-1393.131	20	2500	0.008	-1416.899	34	2500	0.014
	26	-2345.747	1027.118	7.609	-1863.921	4	2500	0.002	-1907.501	5	2500	0.002
	27	-1919.131	948.605	7.777	-1602.148	41	2500	0.016	-1634.771	60	2500	0.024
	28	-1458.952	635.443	5.683	-1203.707	64	2500	0.026	-1227.617	91	2500	0.036
	29	-1743.448	782.312	6.930	-1411.351	19	2500	0.008	-1440.849	33	2500	0.013
	30	-1201.719	614.202	4.248	-1017.962	163	2500	0.065	-1037.115	206	2500	0.082
	31	-2158.374	893.102	6.538	-1750.564	9	2500	0.004	-1777.164	27	2500	0.011
	32	-2342.766	1157.719	8.683	-1983.270	22	2500	0.009	-2033.561	47	2500	0.019
	33	-2785.252	1045.028	11.058	-2277.534	4	2500	0.002	-2343.396	7	2500	0.003
	34	-2098.357	1006.779	7.811	-1699.343	14	2500	0.006	-1737.035	12	2500	0.005
	35	-1464.785	614.082	6.416	-1215.770	65	2500	0.026	-1245.781	95	2500	0.038
	36	-1421.197	593.345	5.528	-1145.576	17	2500	0.007	-1173.565	38	2500	0.015
	37	-2659.194	1299.770	9.260	-2286.161	42	2500	0.017	-2335.245	74	2500	0.030
	38	-2200.233	723.467	7.963	-1803.420	7	2500	0.003	-1855.344	27	2500	0.011
	39	-1603.529	739.484	6.860	-1339.967	51	2500	0.020	-1371.282	74	2500	0.030
	40	-2333.211	1121.278	8.028	-1975.845	43	2500	0.017	-2018.658	69	2500	0.028
	41	-1690.693	715.101	6.689	-1365.286	20	2500	0.008	-1409.005	37	2500	0.015
	42	-1706.548	757.148	6.218	-1422.012	61	2500	0.024	-1456.713	83	2500	0.033
	43	-1146.380	466.354	3.778	-956.640	115	2500	0.046	-976.117	189	2500	0.076
	44	-2944.924	1027.622	13.108	-2413.685	2	2500	0.001	-2480.762	6	2500	0.002
	45	-2664.198	1378.932	11.553	-2222.526	8	2500	0.003	-2264.624	19	2500	0.008
	46	-1757.377	760.067	7.034	-1417.649	6	2500	0.002	-1447.150	18	2500	0.007
	47	-1041.564	525.637	3.500	-880.035	188	2500	0.075	-895.950	242	2500	0.097
	48	-1928.759	940.054	7.935	-1566.695	16	2500	0.006	-1607.769	24	2500	0.010
	49	-1579.966	723.292	6.778	-1299.262	34	2500	0.014	-1327.637	51	2500	0.020
	50	-1572.418	759.078	4.639	-1277.379	44	2500	0.018	-1296.684	58	2500	0.023
	51	-2786.282	1220.602	9.969	-2354.639	12	2500	0.005	-2407.898	43	2500	0.017

	52	-1444.945	449.765	5.012	-1129.274	15	2500	0.006	-1159.936	25	2500	0.010
	53	-2226.674	1084.402	9.430	-1795.494	7	2500	0.003	-1837.321	10	2500	0.004
	54	-3068.582	1463.738	12.641	-2552.662	4	2500	0.002	-2619.577	11	2500	0.004
	55	-2509.801	1241.270	8.794	-2101.046	21	2500	0.008	-2140.327	32	2500	0.013
	56	-2714.756	1238.110	9.008	-2263.245	16	2500	0.006	-2311.052	25	2500	0.010
	57	-1817.081	893.776	6.752	-1437.310	6	2500	0.002	-1464.599	16	2500	0.006
	58	-1264.384	590.837	3.833	-1025.296	58	2500	0.023	-1046.658	104	2500	0.042
	59	-1648.488	761.133	5.390	-1369.407	40	2500	0.016	-1383.425	87	2500	0.035
	60	-2399.422	1184.564	8.389	-2028.660	27	2500	0.011	-2065.527	55	2500	0.022
	61	-2278.177	1114.621	8.597	-1905.739	16	2500	0.006	-1934.529	35	2500	0.014
	62	-1836.265	568.917	7.438	-1472.166	10	2500	0.004	-1507.012	17	2500	0.007
	63	-2029.594	1055.902	8.789	-1665.947	18	2500	0.007	-1710.121	26	2500	0.010
	64	-2909.453	1384.597	14.137	-2423.005	4	2500	0.002	-2490.433	15	2500	0.006
	65	-2723.828	1280.771	12.566	-2285.381	13	2500	0.005	-2340.859	22	2500	0.009
	66	-2036.141	965.322	7.209	-1701.472	34	2500	0.014	-1738.913	52	2500	0.021
	67	-1346.225	703.177	5.765	-1077.182	36	2500	0.014	-1101.784	48	2500	0.019
	68	-1543.419	773.249	5.195	-1261.994	49	2500	0.020	-1288.573	65	2500	0.026
	69	-2941.070	1402.990	12.994	-2514.440	22	2500	0.009	-2568.859	32	2500	0.013
	70	-1721.328	782.929	5.675	-1416.154	35	2500	0.014	-1444.390	59	2500	0.024
	71	-1766.838	757.257	8.494	-1428.659	11	2500	0.004	-1458.479	17	2500	0.007
	72	-3269.701	1572.628	13.021	-2706.831	5	2500	0.002	-2764.998	8	2500	0.003
	73	-2172.017	1117.498	9.011	-1782.650	12	2500	0.005	-1822.050	24	2500	0.010
	74	-1926.245	931.200	6.808	-1598.272	33	2500	0.013	-1633.840	59	2500	0.024
	75	-3249.748	1524.272	13.010	-2665.975	2	2500	0.001	-2727.422	4	2500	0.002
	76	-1058.959	430.949	3.775	-867.185	78	2500	0.031	-884.450	119	2500	0.048
	77	-2512.683	1233.155	9.833	-2110.627	20	2500	0.008	-2150.319	27	2500	0.011
	78	-3945.702	1725.871	16.373	-3217.985	0	2500	0.000	-3290.091	2	2500	0.001
	79	-1959.356	971.931	8.467	-1636.569	39	2500	0.016	-1680.389	64	2500	0.026
	80	-2625.893	1385.136	9.994	-2223.561	29	2500	0.012	-2269.768	41	2500	0.016
	81	-3311.094	1415.752	14.391	-2730.508	0	2500	0.000	-2798.332	3	2500	0.001
	82	-1484.252	697.042	5.375	-1189.031	35	2500	0.014	-1213.743	33	2500	0.013
	83	-1402.031	660.781	4.481	-1191.740	144	2500	0.058	-1214.360	174	2500	0.070
	84	-3111.383	1470.079	12.734	-2548.499	1	2500	0.000	-2614.015	5	2500	0.002
	85	-1775.337	821.480	6.483	-1521.382	88	2500	0.035	-1545.493	134	2500	0.054
	86	-1910.749	822.925	8.542	-1539.975	6	2500	0.002	-1577.652	19	2500	0.008
	87	-1708.003	802.386	5.466	-1396.807	31	2500	0.012	-1423.645	39	2500	0.016
	88	-1804.006	810.026	6.126	-1491.349	36	2500	0.014	-1528.354	80	2500	0.032
	89	-2844.672	1148.776	12.410	-2367.743	6	2500	0.002	-2432.474	16	2500	0.006
	90	-1906.665	890.129	6.401	-1537.987	17	2500	0.007	-1570.235	29	2500	0.012
	91	-1859.163	796.605	5.855	-1501.546	17	2500	0.007	-1531.449	40	2500	0.016
	92	-1313.510	609.270	4.991	-1085.693	67	2500	0.027	-1110.814	104	2500	0.042
	93	-1676.035	732.626	6.653	-1393.958	44	2500	0.018	-1416.198	64	2500	0.026
	94	-1029.168	333.754	3.868	-852.807	106	2500	0.042	-869.654	173	2500	0.069
	95	-1094.339	455.371	3.600	-926.321	165	2500	0.066	-944.996	231	2500	0.092
	96	-1771.501	765.982	7.608	-1440.924	22	2500	0.009	-1482.271	32	2500	0.013
	97	-1079.911	436.833	3.470	-903.060	156	2500	0.062	-917.151	187	2500	0.075
	98	-1153.675	515.297	4.855	-941.972	72	2500	0.029	-961.425	106	2500	0.042
	99	-2280.880	1127.133	7.515	-1895.857	24	2500	0.010	-1927.206	40	2500	0.016
	100	-3042.410	1459.218	13.253	-2508.276	2	2500	0.001	-2570.728	4	2500	0.002
**Mean**		-2020.004	910.441	7.785	-1664.570	35.5	2500.0	0.014	-1701.725	54.4	2500.0	0.022
**Std. Err.**		62.805	30.469	0.296	52.648	4.2	0.0	0.002	54.033	5.5	0.0	0.002
Arachnida	1	-11027.774	1932.088	17.249	-9641.967	1	2500	0.000	-9826.777	0	2500	0.000
	2	-11181.244	2131.581	17.312	-9664.430	0	2500	0.000	-9853.556	1	2500	0.000
	3	-9804.227	1896.921	14.535	-8640.754	2	2500	0.001	-8815.375	3	2500	0.001
	4	-9337.246	1784.743	14.019	-8161.627	1	2500	0.000	-8322.918	3	2500	0.001
	5	-10085.620	1862.551	15.070	-8890.081	1	2500	0.000	-9075.978	4	2500	0.002
	6	-9610.374	1922.458	14.263	-8539.524	0	2500	0.000	-8708.213	10	2500	0.004
	7	-9282.612	1664.795	14.158	-8200.217	1	2500	0.000	-8359.447	0	2500	0.000
	8	-10207.736	1912.458	15.045	-8904.279	0	2500	0.000	-9087.817	0	2500	0.000
	9	-11524.398	2227.053	17.567	-10001.576	0	2500	0.000	-10209.205	0	2500	0.000
	10	-12468.705	2221.030	19.845	-10680.151	0	2500	0.000	-10873.327	0	2500	0.000
	11	-10671.763	1987.815	16.719	-9291.185	0	2500	0.000	-9458.678	1	2500	0.000
	12	-12770.689	2383.811	20.041	-11081.330	0	2500	0.000	-11293.429	0	2500	0.000
	13	-10498.151	1748.725	16.746	-9173.857	0	2500	0.000	-9348.855	1	2500	0.000
	14	-10498.151	1748.725	16.746	-9173.857	0	2500	0.000	-9348.855	1	2500	0.000
	15	-10117.671	1961.197	15.044	-8958.054	1	2500	0.000	-9142.619	3	2500	0.001

	16	-10973.434	2111.038	16.987	-9569.648	0	2500	0.000	-9760.186	0	2500	0.000
	17	-11053.498	2231.510	16.810	-9580.507	0	2500	0.000	-9777.436	0	2500	0.000
	18	-9962.351	1856.036	14.732	-8820.859	2	2500	0.001	-8992.040	3	2500	0.001
	19	-10085.620	1862.551	15.070	-8890.081	1	2500	0.000	-9075.978	4	2500	0.002
	20	-11043.547	2287.684	16.796	-9634.944	0	2500	0.000	-9831.044	0	2500	0.000
	21	-8818.979	1633.710	13.425	-7694.681	1	2500	0.000	-7854.157	2	2500	0.001
	22	-9835.671	1725.688	14.672	-8791.789	1	2500	0.000	-8960.664	10	2500	0.004
	23	-11196.986	2174.566	17.249	-9762.168	0	2500	0.000	-9951.392	0	2500	0.000
	24	-10681.271	1823.932	16.762	-9388.373	0	2500	0.000	-9571.987	2	2500	0.001
	25	-11057.998	1944.250	17.256	-9645.459	0	2500	0.000	-9845.079	0	2500	0.000
	26	-11371.323	1913.212	18.755	-9843.678	0	2500	0.000	-10022.510	0	2500	0.000
	27	-11140.892	2033.173	17.301	-9689.500	0	2500	0.000	-9846.557	0	2500	0.000
	28	-12063.478	2444.976	18.114	-10491.665	0	2500	0.000	-10693.167	0	2500	0.000
	29	-9315.642	1803.826	13.952	-8246.201	3	2500	0.001	-8397.734	7	2500	0.003
	30	-10346.271	1689.467	16.481	-9075.948	1	2500	0.000	-9248.812	1	2500	0.000
	31	-10735.498	2058.551	16.595	-9201.864	0	2500	0.000	-9383.266	0	2500	0.000
	32	-11359.757	2068.564	17.542	-9946.461	0	2500	0.000	-10148.713	0	2500	0.000
	33	-11887.292	2059.640	19.338	-10239.857	0	2500	0.000	-10429.242	0	2500	0.000
	34	-9873.195	1752.611	14.664	-8800.403	2	2500	0.001	-8961.925	3	2500	0.001
	35	-11359.757	2068.564	17.542	-9946.461	0	2500	0.000	-10148.713	0	2500	0.000
	36	-10520.691	2071.835	15.291	-9314.278	0	2500	0.000	-9486.345	2	2500	0.001
	37	-12426.041	2227.205	19.765	-10764.521	0	2500	0.000	-10960.325	0	2500	0.000
	38	-11690.950	2217.089	17.899	-10098.587	0	2500	0.000	-10275.439	0	2500	0.000
	39	-10654.125	1944.625	16.779	-9191.397	0	2500	0.000	-9378.826	1	2500	0.000
	40	-11532.734	2315.860	17.559	-10082.112	0	2500	0.000	-10278.758	0	2500	0.000
	41	-9742.254	1960.730	14.174	-8575.438	2	2500	0.001	-8762.574	3	2500	0.001
	42	-10069.350	2107.327	14.498	-8899.861	1	2500	0.000	-9091.380	7	2500	0.003
	43	-10041.954	2104.609	14.486	-8892.289	3	2500	0.001	-9061.361	5	2500	0.002
	44	-10671.763	1987.815	16.719	-9291.185	0	2500	0.000	-9458.678	1	2500	0.000
	45	-11013.751	2183.321	16.824	-9582.641	0	2500	0.000	-9757.896	0	2500	0.000
	46	-10396.258	1693.807	16.569	-9027.906	0	2500	0.000	-9192.671	0	2500	0.000
	47	-10735.498	2058.551	16.595	-9201.864	0	2500	0.000	-9383.266	0	2500	0.000
	48	-9337.246	1784.743	14.019	-8161.627	1	2500	0.000	-8322.918	3	2500	0.001
	49	-11678.820	2237.167	17.823	-10150.402	0	2500	0.000	-10352.233	1	2500	0.000
	50	-12255.928	2193.893	19.538	-10573.748	0	2500	0.000	-10784.481	0	2500	0.000
	51	-11895.901	2079.827	19.253	-10307.991	0	2500	0.000	-10515.524	0	2500	0.000
	52	-11245.996	2221.412	17.137	-9650.701	0	2500	0.000	-9846.471	0	2500	0.000
	53	-12468.705	2221.030	19.845	-10680.151	0	2500	0.000	-10873.327	0	2500	0.000
	54	-9285.958	1784.076	13.935	-8230.238	1	2500	0.000	-8391.754	5	2500	0.002
	55	-8993.813	1430.666	13.823	-8005.756	5	2500	0.002	-8165.486	8	2500	0.003
	56	-11027.774	1932.088	17.249	-9641.967	1	2500	0.000	-9826.777	0	2500	0.000
	57	-9789.326	1820.070	14.733	-8655.910	0	2500	0.000	-8826.911	4	2500	0.002
	58	-10270.574	1889.963	15.314	-9042.784	1	2500	0.000	-9222.478	3	2500	0.001
	59	-9029.141	1458.079	13.823	-8012.770	2	2500	0.001	-8162.072	5	2500	0.002
	60	-8661.538	1757.632	11.869	-7816.972	15	2500	0.006	-7950.649	41	2500	0.016
	61	-9102.901	1723.799	12.668	-8214.819	10	2500	0.004	-8357.563	34	2500	0.014
	62	-9835.671	1725.688	14.672	-8791.789	1	2500	0.000	-8960.664	10	2500	0.004
	63	-10891.884	1854.715	17.069	-9490.400	0	2500	0.000	-9659.672	0	2500	0.000
	64	-8335.018	1497.466	11.722	-7473.892	8	2500	0.003	-7609.868	31	2500	0.012
	65	-9851.003	1941.775	14.528	-8650.400	0	2500	0.000	-8819.741	2	2500	0.001
	66	-10328.912	1967.617	15.283	-9086.348	0	2500	0.000	-9255.055	0	2500	0.000
	67	-9102.901	1723.799	12.668	-8214.819	10	2500	0.004	-8357.563	34	2500	0.014
	68	-10041.954	2104.609	14.486	-8892.289	3	2500	0.001	-9061.361	5	2500	0.002
	69	-9254.685	1773.868	13.676	-8047.670	0	2500	0.000	-8198.181	0	2500	0.000
	70	-9504.599	1589.286	14.362	-8485.104	4	2500	0.002	-8629.385	11	2500	0.004
	71	-9298.786	1643.651	14.249	-8123.540	0	2500	0.000	-8280.943	1	2500	0.000
	72	-11242.023	2027.886	17.286	-9877.420	0	2500	0.000	-10062.069	0	2500	0.000
	73	-11140.892	2033.173	17.301	-9689.500	0	2500	0.000	-9846.557	0	2500	0.000
	74	-11681.834	2043.319	19.012	-10149.442	0	2500	0.000	-10354.777	0	2500	0.000
	75	-10361.280	1669.165	16.582	-9021.209	0	2500	0.000	-9177.863	0	2500	0.000
	76	-10117.671	1961.197	15.044	-8958.054	1	2500	0.000	-9142.619	3	2500	0.001
	77	-10671.763	1987.815	16.719	-9291.185	0	2500	0.000	-9458.678	1	2500	0.000
	78	-10678.626	2039.604	16.517	-9247.027	1	2500	0.000	-9439.533	0	2500	0.000
	79	-10136.161	1917.811	15.056	-8900.551	0	2500	0.000	-9075.338	4	2500	0.002
	80	-8661.538	1757.632	11.869	-7816.972	15	2500	0.006	-7950.649	41	2500	0.016
	81	-9216.458	1743.289	13.676	-8037.375	0	2500	0.000	-8197.738	2	2500	0.001

	82	-10688.623	1967.034	16.789	-9188.719	0	2500	0.000	-9378.609	0	2500	0.000
	83	-12063.478	2444.976	18.114	-10491.665	0	2500	0.000	-10693.167	0	2500	0.000
	84	-8848.184	1624.036	12.401	-7840.009	3	2500	0.001	-7993.323	5	2500	0.002
	85	-9756.906	1938.738	14.253	-8523.701	0	2500	0.000	-8683.264	2	2500	0.001
	86	-9727.571	1929.448	14.238	-8525.636	0	2500	0.000	-8676.469	1	2500	0.000
	87	-9572.404	1768.307	14.471	-8489.133	1	2500	0.000	-8658.767	11	2500	0.004
	88	-11013.751	2183.321	16.824	-9582.641	0	2500	0.000	-9757.896	0	2500	0.000
	89	-9636.343	1723.339	14.413	-8499.353	2	2500	0.001	-8694.153	6	2500	0.002
	90	-8661.538	1757.632	11.869	-7816.972	15	2500	0.006	-7950.649	41	2500	0.016
	91	-12792.730	2387.307	20.073	-11076.033	0	2500	0.000	-11292.074	0	2500	0.000
	92	-9804.158	1781.105	14.743	-8598.028	1	2500	0.000	-8772.753	3	2500	0.001
	93	-11205.377	2186.031	17.146	-9659.961	0	2500	0.000	-9857.953	0	2500	0.000
	94	-8284.131	1439.791	11.729	-7443.987	13	2500	0.005	-7588.493	27	2500	0.011
	95	-10597.394	2035.499	15.624	-9366.440	0	2500	0.000	-9551.910	4	2500	0.002
	96	-11042.477	1908.594	17.327	-9567.693	0	2500	0.000	-9740.088	0	2500	0.000
	97	-10681.271	1823.932	16.762	-9388.373	0	2500	0.000	-9571.987	2	2500	0.001
	98	-9868.962	1921.728	14.608	-8588.438	1	2500	0.000	-8745.488	2	2500	0.001
	99	-11140.892	2033.173	17.301	-9689.500	0	2500	0.000	-9846.557	0	2500	0.000
	100	-12018.597	2390.515	18.125	-10469.944	0	2500	0.000	-10684.815	0	2500	0.000
**Mean**		-10455.463	1945.846	15.912	-9153.826	1.4	2500.0	0.000	-9329.885	4.3	2500.0	0.002
**Std. Err.**		104.012	22.464	0.204	82.979	0.3	0.0	0.000	84.588	0.9	0.0	0.000
Malacostraca	1	-4833.935	2717.180	20.555	-4184.077	4	2500	0.002	-4269.157	18	2500	0.007
	2	-4151.055	2418.584	18.145	-3575.953	8	2500	0.003	-3646.770	17	2500	0.007
	3	-6803.217	3187.175	34.929	-5643.022	0	2500	0.000	-5787.108	0	2500	0.000
	4	-5438.202	3097.302	23.325	-4697.983	2	2500	0.001	-4796.480	4	2500	0.002
	5	-6398.393	3636.972	28.585	-5493.655	0	2500	0.000	-5617.375	0	2500	0.000
	6	-5698.305	3254.441	23.664	-4973.998	3	2500	0.001	-5071.153	13	2500	0.005
	7	-5502.996	3040.517	25.604	-4678.921	0	2500	0.000	-4781.840	1	2500	0.000
	8	-5698.305	3254.441	23.664	-4973.998	3	2500	0.001	-5071.153	13	2500	0.005
	9	-3925.308	2280.202	17.357	-3367.550	13	2500	0.005	-3429.534	20	2500	0.008
	10	-7987.676	4035.893	38.293	-6724.947	0	2500	0.000	-6866.269	0	2500	0.000
	11	-6879.342	3237.914	35.097	-5722.788	0	2500	0.000	-5862.206	0	2500	0.000
	12	-6398.393	3636.972	28.585	-5493.655	0	2500	0.000	-5617.375	0	2500	0.000
	13	-5525.039	3036.261	25.126	-4722.160	0	2500	0.000	-4830.370	1	2500	0.000
	14	-5685.901	3141.235	25.566	-4864.416	0	2500	0.000	-4963.478	3	2500	0.001
	15	-4677.042	2624.909	19.999	-4024.204	3	2500	0.001	-4099.618	13	2500	0.005
	16	-6710.384	3112.434	34.697	-5580.590	0	2500	0.000	-5704.762	0	2500	0.000
	17	-5525.039	3036.261	25.126	-4722.160	0	2500	0.000	-4830.370	1	2500	0.000
	18	-6414.328	3611.354	28.126	-5538.204	0	2500	0.000	-5645.360	7	2500	0.003
	19	-4754.757	2690.643	20.308	-4108.152	3	2500	0.001	-4188.083	13	2500	0.005
	20	-3102.057	1795.438	14.215	-2616.010	10	2500	0.004	-2669.677	22	2500	0.009
	21	-6686.001	3110.797	35.191	-5520.048	0	2500	0.000	-5672.310	0	2500	0.000
	22	-3167.508	1810.031	14.450	-2683.897	7	2500	0.003	-2728.378	8	2500	0.003
	23	-4494.994	2515.954	19.996	-3844.418	2	2500	0.001	-3930.632	14	2500	0.006
	24	-3248.807	1882.378	14.690	-2742.116	7	2500	0.003	-2799.047	14	2500	0.006
	25	-5601.707	3081.320	25.323	-4788.304	0	2500	0.000	-4905.181	3	2500	0.001
	26	-3000.946	1735.812	14.441	-2506.697	7	2500	0.003	-2562.698	21	2500	0.008
	27	-6414.328	3611.354	28.126	-5538.204	0	2500	0.000	-5645.360	7	2500	0.003
	28	-4519.457	2512.385	19.548	-3887.043	5	2500	0.002	-3973.633	21	2500	0.008
	29	-5448.220	3000.383	24.906	-4665.939	1	2500	0.000	-4758.185	7	2500	0.003
	30	-3167.508	1810.031	14.450	-2683.897	7	2500	0.003	-2728.378	8	2500	0.003
	31	-6803.217	3187.175	34.929	-5643.022	0	2500	0.000	-5787.108	0	2500	0.000
	32	-4151.055	2418.584	18.145	-3575.953	8	2500	0.003	-3646.770	17	2500	0.007
	33	-6710.384	3112.434	34.697	-5580.590	0	2500	0.000	-5704.762	0	2500	0.000
	34	-3245.764	1880.376	14.770	-2759.396	12	2500	0.005	-2816.455	29	2500	0.012
	35	-7054.603	3371.831	35.559	-5870.891	0	2500	0.000	-6027.383	0	2500	0.000
	36	-6974.609	3341.072	35.293	-5807.519	0	2500	0.000	-5933.626	0	2500	0.000
	37	-4494.994	2515.954	19.996	-3844.418	2	2500	0.001	-3930.632	14	2500	0.006
	38	-3000.946	1735.812	14.441	-2506.697	7	2500	0.003	-2562.698	21	2500	0.008
	39	-4602.556	2586.027	19.796	-3977.854	8	2500	0.003	-4046.224	8	2500	0.003
	40	-4580.021	2582.236	20.267	-3917.500	4	2500	0.002	-3999.732	5	2500	0.002
	41	-4677.042	2624.909	19.999	-4024.204	3	2500	0.001	-4099.618	13	2500	0.005
	42	-4519.457	2512.385	19.548	-3887.043	5	2500	0.002	-3973.633	21	2500	0.008
	43	-5438.202	3097.302	23.325	-4697.983	2	2500	0.001	-4796.480	4	2500	0.002
	44	-6803.217	3187.175	34.929	-5643.022	0	2500	0.000	-5787.108	0	2500	0.000
	45	-3321.667	1928.093	15.022	-2828.543	7	2500	0.003	-2882.354	14	2500	0.006

	46	-4754.757	2690.643	20.308	-4108.152	3	2500	0.001	-4188.083	13	2500	0.005
	47	-3167.508	1810.031	14.450	-2683.897	7	2500	0.003	-2728.378	8	2500	0.003
	48	-6978.139	3348.017	35.341	-5810.898	0	2500	0.000	-5949.717	0	2500	0.000
	49	-3167.508	1810.031	14.450	-2683.897	7	2500	0.003	-2728.378	8	2500	0.003
	50	-4833.935	2717.180	20.555	-4184.077	4	2500	0.002	-4269.157	18	2500	0.007
	51	-5502.996	3040.517	25.604	-4678.921	0	2500	0.000	-4781.840	1	2500	0.000
	52	-6658.071	3780.076	28.877	-5754.188	0	2500	0.000	-5881.170	2	2500	0.001
	53	-4754.757	2690.643	20.308	-4108.152	3	2500	0.001	-4188.083	13	2500	0.005
	54	-4833.935	2717.180	20.555	-4184.077	4	2500	0.002	-4269.157	18	2500	0.007
	55	-3027.335	1760.535	13.976	-2562.798	10	2500	0.004	-2611.744	19	2500	0.008
	56	-3321.667	1928.093	15.022	-2828.543	7	2500	0.003	-2882.354	14	2500	0.006
	57	-3925.308	2280.202	17.357	-3367.550	13	2500	0.005	-3429.534	20	2500	0.008
	58	-5438.202	3097.302	23.325	-4697.983	2	2500	0.001	-4796.480	4	2500	0.002
	59	-3027.335	1760.535	13.976	-2562.798	10	2500	0.004	-2611.744	19	2500	0.008
	60	-6398.393	3636.972	28.585	-5493.655	0	2500	0.000	-5617.375	0	2500	0.000
	61	-6974.609	3341.072	35.293	-5807.519	0	2500	0.000	-5933.626	0	2500	0.000
	62	-6686.001	3110.797	35.191	-5520.048	0	2500	0.000	-5672.310	0	2500	0.000
	63	-6879.342	3237.914	35.097	-5722.788	0	2500	0.000	-5862.206	0	2500	0.000
	64	-4677.042	2624.909	19.999	-4024.204	3	2500	0.001	-4099.618	13	2500	0.005
	65	-7733.713	3839.371	37.608	-6482.761	0	2500	0.000	-6636.336	0	2500	0.000
	66	-5448.220	3000.383	24.906	-4665.939	1	2500	0.000	-4758.185	7	2500	0.003
	67	-3321.667	1928.093	15.022	-2828.543	7	2500	0.003	-2882.354	14	2500	0.006
	68	-6658.071	3780.076	28.877	-5754.188	0	2500	0.000	-5881.170	2	2500	0.001
	69	-3245.764	1880.376	14.770	-2759.396	12	2500	0.005	-2816.455	29	2500	0.012
	70	-4677.042	2624.909	19.999	-4024.204	3	2500	0.001	-4099.618	13	2500	0.005
	71	-5619.898	3194.404	23.340	-4893.123	6	2500	0.002	-4979.832	12	2500	0.005
	72	-6686.001	3110.797	35.191	-5520.048	0	2500	0.000	-5672.310	0	2500	0.000
	73	-6803.217	3187.175	34.929	-5643.022	0	2500	0.000	-5787.108	0	2500	0.000
	74	-4580.021	2582.236	20.267	-3917.500	4	2500	0.002	-3999.732	5	2500	0.002
	75	-4519.457	2512.385	19.548	-3887.043	5	2500	0.002	-3973.633	21	2500	0.008
	76	-5420.502	2975.848	25.366	-4610.871	1	2500	0.000	-4713.287	2	2500	0.001
	77	-5619.898	3194.404	23.340	-4893.123	6	2500	0.002	-4979.832	12	2500	0.005
	78	-6414.328	3611.354	28.126	-5538.204	0	2500	0.000	-5645.360	7	2500	0.003
	79	-4677.042	2624.909	19.999	-4024.204	3	2500	0.001	-4099.618	13	2500	0.005
	80	-4757.864	2691.283	20.245	-4086.157	3	2500	0.001	-4180.111	9	2500	0.004
	81	-6803.217	3187.175	34.929	-5643.022	0	2500	0.000	-5787.108	0	2500	0.000
	82	-5525.039	3036.261	25.126	-4722.160	0	2500	0.000	-4830.370	1	2500	0.000
	83	-4580.021	2582.236	20.267	-3917.500	4	2500	0.002	-3999.732	5	2500	0.002
	84	-6879.342	3237.914	35.097	-5722.788	0	2500	0.000	-5862.206	0	2500	0.000
	85	-5502.996	3040.517	25.604	-4678.921	0	2500	0.000	-4781.840	1	2500	0.000
	86	-5686.206	3152.209	25.634	-4887.479	1	2500	0.000	-4978.141	4	2500	0.002
	87	-6710.384	3112.434	34.697	-5580.590	0	2500	0.000	-5704.762	0	2500	0.000
	88	-7706.328	3818.835	38.080	-6448.752	0	2500	0.000	-6593.921	0	2500	0.000
	89	-5601.707	3081.320	25.323	-4788.304	0	2500	0.000	-4905.181	3	2500	0.001
	90	-3925.308	2280.202	17.357	-3367.550	13	2500	0.005	-3429.534	20	2500	0.008
	91	-6686.001	3110.797	35.191	-5520.048	0	2500	0.000	-5672.310	0	2500	0.000
	92	-5698.305	3254.441	23.664	-4973.998	3	2500	0.001	-5071.153	13	2500	0.005
	93	-7987.676	4035.893	38.293	-6724.947	0	2500	0.000	-6866.269	0	2500	0.000
	94	-3925.308	2280.202	17.357	-3367.550	13	2500	0.005	-3429.534	20	2500	0.008
	95	-4833.935	2717.180	20.555	-4184.077	4	2500	0.002	-4269.157	18	2500	0.007
	96	-6658.071	3780.076	28.877	-5754.188	0	2500	0.000	-5881.170	2	2500	0.001
	97	-6414.328	3611.354	28.126	-5538.204	0	2500	0.000	-5645.360	7	2500	0.003
	98	-4677.042	2624.909	19.999	-4024.204	3	2500	0.001	-4099.618	13	2500	0.005
	99	-4833.935	2717.180	20.555	-4184.077	4	2500	0.002	-4269.157	18	2500	0.007
	100	-3248.807	1882.378	14.690	-2742.116	7	2500	0.003	-2799.047	14	2500	0.006
**Mean**		-5293.104	2846.621	24.420	-4502.146	3.2	2500.0	0.001	-4599.280	8.5	2500.0	0.003
**Std. Err.**		134.182	59.529	0.736	111.284	0.4	0.0	0.000	114.278	0.8	0.0	0.000
Insecta	1	-68503.388	28383.953	12.962	-58437.104	0	2500	0.000	-59331.925	0	2500	0.000
	2	-66953.998	29313.871	13.127	-56734.511	0	2500	0.000	-57630.679	0	2500	0.000
	3	-67442.725	28740.506	12.710	-57365.792	0	2500	0.000	-58177.218	0	2500	0.000
	4	-66128.991	28076.283	12.350	-56187.908	0	2500	0.000	-57030.326	0	2500	0.000
	5	-66039.101	28972.401	12.329	-56154.573	0	2500	0.000	-57009.953	0	2500	0.000
	6	-70814.798	30614.276	13.684	-60053.431	0	2500	0.000	-60966.817	0	2500	0.000
	7	-70862.842	26582.009	13.642	-60587.728	0	2500	0.000	-61619.931	0	2500	0.000
	8	-68198.662	29270.690	12.777	-58255.731	0	2500	0.000	-59103.519	0	2500	0.000
	9	-68696.889	29930.787	13.329	-58272.292	0	2500	0.000	-59169.252	0	2500	0.000

	10	-72633.908	28906.772	13.931	-61885.955	0	2500	0.000	-62887.997	0	2500	0.000
	11	-63128.103	27658.383	11.743	-53666.966	0	2500	0.000	-54483.420	0	2500	0.000
	12	-74620.555	29641.702	14.741	-63493.052	0	2500	0.000	-64551.274	0	2500	0.000
	13	-73621.761	32000.558	14.185	-62309.539	0	2500	0.000	-63232.777	0	2500	0.000
	14	-69171.561	29175.974	12.816	-58906.904	0	2500	0.000	-59779.482	0	2500	0.000
	15	-75560.308	32626.587	14.670	-64200.094	0	2500	0.000	-65206.697	0	2500	0.000
	16	-69204.537	28817.587	13.074	-59040.334	0	2500	0.000	-59950.599	0	2500	0.000
	17	-69678.353	29523.550	13.586	-59373.767	0	2500	0.000	-60301.092	0	2500	0.000
	18	-66434.882	27493.219	12.400	-56620.463	0	2500	0.000	-57472.503	0	2500	0.000
	19	-67906.818	28542.824	12.886	-57768.893	0	2500	0.000	-58659.156	0	2500	0.000
	20	-69019.743	25570.100	13.230	-59060.191	0	2500	0.000	-60014.948	0	2500	0.000
	21	-79250.314	31539.315	15.469	-67410.036	0	2500	0.000	-68527.862	0	2500	0.000
	22	-75022.791	33286.965	14.531	-63599.643	0	2500	0.000	-64560.669	0	2500	0.000
	23	-69485.145	28727.549	13.026	-59352.353	0	2500	0.000	-60251.491	0	2500	0.000
	24	-66923.737	29276.772	12.419	-56678.063	0	2500	0.000	-57542.954	0	2500	0.000
	25	-69993.664	29573.377	13.134	-59501.723	0	2500	0.000	-60393.460	0	2500	0.000
	26	-66916.401	29235.136	12.557	-56911.383	0	2500	0.000	-57742.112	0	2500	0.000
	27	-77511.104	30026.792	14.706	-66258.595	0	2500	0.000	-67320.088	0	2500	0.000
	28	-71943.174	30651.312	13.835	-61067.483	0	2500	0.000	-61969.640	0	2500	0.000
	29	-66798.499	28049.931	12.476	-57218.902	0	2500	0.000	-58098.173	0	2500	0.000
	30	-71849.976	30947.756	13.487	-60909.335	0	2500	0.000	-61863.898	0	2500	0.000
	31	-69928.127	26974.244	13.668	-59975.483	0	2500	0.000	-60979.158	0	2500	0.000
	32	-67451.162	29082.613	13.196	-57506.698	0	2500	0.000	-58406.240	0	2500	0.000
	33	-66735.451	29239.386	12.415	-56674.913	0	2500	0.000	-57537.352	0	2500	0.000
	34	-69331.386	30014.138	13.363	-58681.953	0	2500	0.000	-59557.148	0	2500	0.000
	35	-66823.312	28666.721	13.067	-56734.953	0	2500	0.000	-57639.686	0	2500	0.000
	36	-70159.582	30360.098	13.723	-59637.184	0	2500	0.000	-60582.253	0	2500	0.000
	37	-69696.798	30241.743	13.230	-59682.008	0	2500	0.000	-60569.153	0	2500	0.000
	38	-68487.753	28486.498	12.903	-58637.576	0	2500	0.000	-59508.283	0	2500	0.000
	39	-71010.405	30525.327	13.805	-60226.787	0	2500	0.000	-61201.606	0	2500	0.000
	40	-75962.264	33323.371	14.746	-64345.283	0	2500	0.000	-65278.816	0	2500	0.000
	41	-69837.057	30758.883	13.592	-59108.338	0	2500	0.000	-60028.391	0	2500	0.000
	42	-66449.537	25091.521	13.025	-57165.880	0	2500	0.000	-58156.769	0	2500	0.000
	43	-73857.599	32140.906	14.209	-62768.279	0	2500	0.000	-63701.361	0	2500	0.000
	44	-67566.432	28806.464	12.523	-57539.415	0	2500	0.000	-58441.493	0	2500	0.000
	45	-70446.005	30247.280	13.676	-60004.696	0	2500	0.000	-60912.826	0	2500	0.000
	46	-75129.736	29900.572	14.865	-63963.216	0	2500	0.000	-65028.089	0	2500	0.000
	47	-68156.497	29812.434	13.241	-57958.340	0	2500	0.000	-58858.307	0	2500	0.000
	48	-66352.799	29224.325	12.985	-56335.203	0	2500	0.000	-57207.126	0	2500	0.000
	49	-68874.700	29226.013	12.826	-58709.104	0	2500	0.000	-59616.825	0	2500	0.000
	50	-62541.166	25669.316	11.805	-53598.740	0	2500	0.000	-54432.867	0	2500	0.000
	51	-75543.158	29267.330	14.576	-64719.831	0	2500	0.000	-65754.220	0	2500	0.000
	52	-70956.785	31117.895	13.808	-60139.484	0	2500	0.000	-61055.080	0	2500	0.000
	53	-77488.565	30592.889	15.250	-66095.536	0	2500	0.000	-67152.056	0	2500	0.000
	54	-70337.987	29528.409	13.090	-59973.775	0	2500	0.000	-60885.689	0	2500	0.000
	55	-70154.205	29647.751	13.050	-59752.384	0	2500	0.000	-60659.962	0	2500	0.000
	56	-67061.931	28701.730	12.638	-57190.993	0	2500	0.000	-58063.595	0	2500	0.000
	57	-69873.165	30630.646	13.063	-59344.949	0	2500	0.000	-60175.787	0	2500	0.000
	58	-67244.353	29043.692	13.046	-57202.053	0	2500	0.000	-58040.184	0	2500	0.000
	59	-69676.708	30065.224	13.582	-59254.235	0	2500	0.000	-60135.150	0	2500	0.000
	60	-74073.144	32567.160	14.357	-62981.014	0	2500	0.000	-63976.214	0	2500	0.000
	61	-66404.893	28586.084	12.644	-56864.596	0	2500	0.000	-57730.566	0	2500	0.000
	62	-68793.045	28741.309	12.940	-58865.536	0	2500	0.000	-59773.898	0	2500	0.000
	63	-72957.845	32239.399	14.094	-61637.133	0	2500	0.000	-62585.274	0	2500	0.000
	64	-72223.018	28748.411	13.825	-61602.455	0	2500	0.000	-62583.867	0	2500	0.000
	65	-72270.164	31340.949	14.012	-61235.098	0	2500	0.000	-62166.910	0	2500	0.000
	66	-67340.646	29763.128	13.084	-57184.229	0	2500	0.000	-58024.321	0	2500	0.000
	67	-67405.048	28119.412	12.898	-57515.945	0	2500	0.000	-58435.233	0	2500	0.000
	68	-61375.546	24902.547	11.803	-52911.384	0	2500	0.000	-53758.853	0	2500	0.000
	69	-71262.105	30331.695	13.254	-60495.320	0	2500	0.000	-61389.697	0	2500	0.000
	70	-68475.179	29510.761	13.397	-58096.938	0	2500	0.000	-59021.654	0	2500	0.000
	71	-67706.961	28181.255	12.910	-57892.168	0	2500	0.000	-58759.166	0	2500	0.000
	72	-66925.317	27908.811	12.483	-56964.361	0	2500	0.000	-57893.026	0	2500	0.000
	73	-70559.745	27277.578	14.055	-60320.000	0	2500	0.000	-61350.981	0	2500	0.000
	74	-77932.240	34095.080	14.899	-66399.755	0	2500	0.000	-67367.752	0	2500	0.000
	75	-68936.730	28728.728	12.943	-59029.281	0	2500	0.000	-59925.612	0	2500	0.000

	76	-72832.940	30790.097	13.777	-62210.332	0	2500	0.000	-63167.012	0	2500	0.000
	77	-65226.597	28552.859	12.790	-55249.667	0	2500	0.000	-56129.103	0	2500	0.000
	78	-70949.584	29119.532	13.298	-60590.690	0	2500	0.000	-61492.778	0	2500	0.000
	79	-65370.548	27619.779	12.375	-55796.647	0	2500	0.000	-56685.919	0	2500	0.000
	80	-73545.808	31290.600	14.215	-62588.361	0	2500	0.000	-63548.714	0	2500	0.000
	81	-71048.389	29900.690	13.295	-60472.890	0	2500	0.000	-61455.411	0	2500	0.000
	82	-74395.580	32138.748	14.472	-63175.783	0	2500	0.000	-64158.993	0	2500	0.000
	83	-63034.974	27355.675	11.891	-53600.064	0	2500	0.000	-54409.803	0	2500	0.000
	84	-66861.766	29331.582	12.456	-56953.443	0	2500	0.000	-57823.415	0	2500	0.000
	85	-72267.174	30125.915	13.710	-61769.448	0	2500	0.000	-62697.461	0	2500	0.000
	86	-73695.455	27756.473	14.143	-63282.407	0	2500	0.000	-64272.193	0	2500	0.000
	87	-72822.949	31072.880	13.643	-62050.658	0	2500	0.000	-62979.980	0	2500	0.000
	88	-68090.724	28952.011	12.790	-58110.204	0	2500	0.000	-59017.250	0	2500	0.000
	89	-69292.921	28940.547	12.967	-59199.733	0	2500	0.000	-60034.887	0	2500	0.000
	90	-69136.174	28594.402	13.025	-59106.568	0	2500	0.000	-60038.285	0	2500	0.000
	91	-64520.445	27926.238	11.935	-54821.395	0	2500	0.000	-55680.237	0	2500	0.000
	92	-68495.035	28798.086	12.916	-58677.747	0	2500	0.000	-59618.618	0	2500	0.000
	93	-69646.198	30500.203	12.802	-59013.913	0	2500	0.000	-59876.392	0	2500	0.000
	94	-67179.025	27725.113	12.822	-57600.011	0	2500	0.000	-58466.139	0	2500	0.000
	95	-68270.414	28727.694	12.736	-57971.731	0	2500	0.000	-58817.330	0	2500	0.000
	96	-70406.733	30637.993	13.777	-59768.549	0	2500	0.000	-60694.717	0	2500	0.000
	97	-65814.917	24756.019	12.691	-56356.288	0	2500	0.000	-57319.212	0	2500	0.000
	98	-72619.009	29976.283	13.648	-61971.678	0	2500	0.000	-62930.622	0	2500	0.000
	99	-66816.432	29259.400	12.474	-56829.484	0	2500	0.000	-57705.362	0	2500	0.000
	100	-72657.890	27846.826	14.247	-62334.920	0	2500	0.000	-63342.149	0	2500	0.000
**Mean**		-69730.907	29362.823	13.313	-59397.079	0.0	2500.0	0.000	-60315.204	0.0	2500.0	0.000
**Std. Err.**		344.867	172.368	0.079	291.293	0.0	0.0	0.000	296.265	0.0	0.0	0.000
Chondrichthyes	1	-9312.508	4637.415	36.441	-7818.563	0	2500	0.000	-8004.807	0	2500	0.000
	2	-5993.754	2002.475	25.439	-5145.095	0	2500	0.000	-5295.018	3	2500	0.001
	3	-9312.508	4637.415	36.441	-7818.563	0	2500	0.000	-8004.807	0	2500	0.000
	4	-9386.271	4351.925	36.964	-7867.094	0	2500	0.000	-8059.577	0	2500	0.000
	5	-5287.241	1774.502	24.277	-4541.219	2	2500	0.001	-4682.468	16	2500	0.006
	6	-5748.254	1692.983	25.647	-4923.797	1	2500	0.000	-5073.955	0	2500	0.000
	7	-6706.202	2379.270	27.587	-5668.701	0	2500	0.000	-5831.286	1	2500	0.000
	8	-9348.589	4469.750	36.494	-7865.516	0	2500	0.000	-8053.057	0	2500	0.000
	9	-10132.566	5113.868	39.275	-8424.773	0	2500	0.000	-8624.633	0	2500	0.000
	10	-6360.157	2469.662	26.648	-5369.718	1	2500	0.000	-5514.502	1	2500	0.000
	11	-5546.124	1969.104	24.695	-4758.639	0	2500	0.000	-4915.764	13	2500	0.005
	12	-10132.566	5113.868	39.275	-8424.773	0	2500	0.000	-8624.633	0	2500	0.000
	13	-5220.259	1599.580	24.741	-4467.966	0	2500	0.000	-4612.971	5	2500	0.002
	14	-9247.197	4621.883	36.276	-7703.281	0	2500	0.000	-7893.577	0	2500	0.000
	15	-6836.999	2523.502	27.768	-5788.332	0	2500	0.000	-5952.986	1	2500	0.000
	16	-9407.221	4761.815	36.574	-7907.212	0	2500	0.000	-8083.033	0	2500	0.000
	17	-6436.443	2463.812	26.987	-5465.504	0	2500	0.000	-5608.586	2	2500	0.001
	18	-9539.432	4691.798	37.102	-7984.065	0	2500	0.000	-8176.438	0	2500	0.000
	19	-6706.202	2379.270	27.587	-5668.701	0	2500	0.000	-5831.286	1	2500	0.000
	20	-5134.641	1537.327	24.757	-4401.078	0	2500	0.000	-4547.307	10	2500	0.004
	21	-6778.155	2564.936	27.771	-5740.682	0	2500	0.000	-5901.983	0	2500	0.000
	22	-6800.867	2549.957	29.727	-5761.835	0	2500	0.000	-5941.456	0	2500	0.000
	23	-10561.644	4791.703	44.366	-8868.967	0	2500	0.000	-9096.097	0	2500	0.000
	24	-7764.240	3281.051	32.269	-6549.930	0	2500	0.000	-6715.776	0	2500	0.000
	25	-5690.992	1909.005	24.856	-4845.320	0	2500	0.000	-5002.259	4	2500	0.002
	26	-10251.300	4771.816	39.845	-8565.503	0	2500	0.000	-8767.345	0	2500	0.000
	27	-6164.891	2203.492	26.479	-5225.667	0	2500	0.000	-5381.309	1	2500	0.000
	28	-5748.254	1692.983	25.647	-4923.797	1	2500	0.000	-5073.955	0	2500	0.000
	29	-10706.872	5116.648	44.286	-9011.964	0	2500	0.000	-9239.594	0	2500	0.000
	30	-6985.476	2752.011	30.110	-5939.731	0	2500	0.000	-6103.713	0	2500	0.000
	31	-6360.157	2469.662	26.648	-5369.718	1	2500	0.000	-5514.502	1	2500	0.000
	32	-5675.256	1838.672	24.951	-4870.669	1	2500	0.000	-5027.643	7	2500	0.003
	33	-6100.269	2229.219	26.390	-5137.983	0	2500	0.000	-5285.304	0	2500	0.000
	34	-5502.725	1891.498	24.474	-4721.961	0	2500	0.000	-4865.830	12	2500	0.005
	35	-9240.841	4659.419	36.362	-7686.650	0	2500	0.000	-7870.971	0	2500	0.000
	36	-11372.523	5910.954	44.770	-9508.165	0	2500	0.000	-9721.558	0	2500	0.000
	37	-9251.875	4646.136	36.364	-7731.485	0	2500	0.000	-7922.942	0	2500	0.000
	38	-11517.300	5452.304	47.095	-9645.573	0	2500	0.000	-9875.001	0	2500	0.000
	39	-6626.500	2494.654	27.211	-5599.973	0	2500	0.000	-5757.855	0	2500	0.000

	40	-7764.240	3281.051	32.269	-6549.930	0	2500	0.000	-6715.776	0	2500	0.000
	41	-5161.218	1434.376	24.978	-4429.727	1	2500	0.000	-4575.298	7	2500	0.003
	42	-5523.246	1595.715	25.386	-4717.115	0	2500	0.000	-4865.889	3	2500	0.001
	43	-5923.919	1866.686	25.773	-5045.219	1	2500	0.000	-5202.159	3	2500	0.001
	44	-5386.850	1717.195	24.993	-4601.710	2	2500	0.001	-4756.672	3	2500	0.001
	45	-6436.443	2463.812	26.987	-5465.504	0	2500	0.000	-5608.586	2	2500	0.001
	46	-9407.221	4761.815	36.574	-7907.212	0	2500	0.000	-8083.033	0	2500	0.000
	47	-9247.197	4621.883	36.276	-7703.281	0	2500	0.000	-7893.577	0	2500	0.000
	48	-9240.841	4659.419	36.362	-7686.650	0	2500	0.000	-7870.971	0	2500	0.000
	49	-5949.178	2060.767	25.389	-5100.576	2	2500	0.001	-5248.517	2	2500	0.001
	50	-5993.754	2002.475	25.439	-5145.095	0	2500	0.000	-5295.018	3	2500	0.001
	51	-9655.564	4629.806	37.668	-8102.030	0	2500	0.000	-8283.258	0	2500	0.000
	52	-9287.634	4471.478	36.371	-7777.117	0	2500	0.000	-7958.555	0	2500	0.000
	53	-6985.476	2752.011	30.110	-5939.731	0	2500	0.000	-6103.713	0	2500	0.000
	54	-5752.312	1897.911	25.291	-4881.392	0	2500	0.000	-5032.117	0	2500	0.000
	55	-5594.641	1770.905	25.464	-4797.787	1	2500	0.000	-4954.620	2	2500	0.001
	56	-6706.202	2379.270	27.587	-5668.701	0	2500	0.000	-5831.286	1	2500	0.000
	57	-5134.641	1537.327	24.757	-4401.078	0	2500	0.000	-4547.307	10	2500	0.004
	58	-9334.440	4732.193	36.515	-7810.961	0	2500	0.000	-7984.715	0	2500	0.000
	59	-9445.397	4641.883	37.083	-7922.395	0	2500	0.000	-8095.111	0	2500	0.000
	60	-6626.500	2494.654	27.211	-5599.973	0	2500	0.000	-5757.855	0	2500	0.000
	61	-5845.299	1764.236	25.763	-4995.313	0	2500	0.000	-5160.377	1	2500	0.000
	62	-5523.246	1595.715	25.386	-4717.115	0	2500	0.000	-4865.889	3	2500	0.001
	63	-5675.256	1838.672	24.951	-4870.669	1	2500	0.000	-5027.643	7	2500	0.003
	64	-9489.803	4774.498	37.137	-7942.316	0	2500	0.000	-8123.450	0	2500	0.000
	65	-9407.221	4761.815	36.574	-7907.212	0	2500	0.000	-8083.033	0	2500	0.000
	66	-6985.476	2752.011	30.110	-5939.731	0	2500	0.000	-6103.713	0	2500	0.000
	67	-10104.610	4936.201	39.207	-8420.104	0	2500	0.000	-8599.606	0	2500	0.000
	68	-6567.542	2290.837	27.211	-5559.425	0	2500	0.000	-5720.744	0	2500	0.000
	69	-5121.544	1534.252	24.764	-4354.356	0	2500	0.000	-4500.038	2	2500	0.001
	70	-5949.178	2060.767	25.389	-5100.576	2	2500	0.001	-5248.517	2	2500	0.001
	71	-6626.500	2494.654	27.211	-5599.973	0	2500	0.000	-5757.855	0	2500	0.000
	72	-5523.246	1595.715	25.386	-4717.115	0	2500	0.000	-4865.889	3	2500	0.001
	73	-9247.197	4621.883	36.276	-7703.281	0	2500	0.000	-7893.577	0	2500	0.000
	74	-11083.691	5020.937	45.221	-9323.048	0	2500	0.000	-9562.257	0	2500	0.000
	75	-6944.027	2812.030	29.596	-5910.245	0	2500	0.000	-6070.455	2	2500	0.001
	76	-6022.341	1913.220	26.032	-5145.294	1	2500	0.000	-5308.756	6	2500	0.002
	77	-6586.213	2687.463	29.319	-5610.112	0	2500	0.000	-5772.015	0	2500	0.000
	78	-9386.271	4351.925	36.964	-7867.094	0	2500	0.000	-8059.577	0	2500	0.000
	79	-9419.633	4301.192	37.069	-7913.781	0	2500	0.000	-8098.282	0	2500	0.000
	80	-6100.269	2229.219	26.390	-5137.983	0	2500	0.000	-5285.304	0	2500	0.000
	81	-9348.589	4469.750	36.494	-7865.516	0	2500	0.000	-8053.057	0	2500	0.000
	82	-9655.564	4629.806	37.668	-8102.030	0	2500	0.000	-8283.258	0	2500	0.000
	83	-10490.296	5523.774	41.822	-8775.434	0	2500	0.000	-8976.017	0	2500	0.000
	84	-10612.621	5488.532	42.363	-8904.486	0	2500	0.000	-9101.663	0	2500	0.000
	85	-10172.669	4908.238	39.281	-8505.789	0	2500	0.000	-8693.196	0	2500	0.000
	86	-5995.928	2021.110	25.875	-5119.782	0	2500	0.000	-5276.326	2	2500	0.001
	87	-10132.566	5113.868	39.275	-8424.773	0	2500	0.000	-8624.633	0	2500	0.000
	88	-11099.821	4826.346	45.776	-9326.611	0	2500	0.000	-9563.809	0	2500	0.000
	89	-9287.634	4471.478	36.371	-7777.117	0	2500	0.000	-7958.555	0	2500	0.000
	90	-10132.540	5058.480	39.257	-8460.520	0	2500	0.000	-8657.995	0	2500	0.000
	91	-9495.196	4335.470	37.516	-7958.853	0	2500	0.000	-8141.342	0	2500	0.000
	92	-5287.241	1774.502	24.277	-4541.219	2	2500	0.001	-4682.468	16	2500	0.006
	93	-6836.999	2523.502	27.768	-5788.332	0	2500	0.000	-5952.986	1	2500	0.000
	94	-5949.178	2060.767	25.389	-5100.576	2	2500	0.001	-5248.517	2	2500	0.001
	95	-10429.440	5128.668	40.007	-8717.853	0	2500	0.000	-8915.149	0	2500	0.000
	96	-5923.919	1866.686	25.773	-5045.219	1	2500	0.000	-5202.159	3	2500	0.001
	97	-10561.644	4791.703	44.366	-8868.967	0	2500	0.000	-9096.097	0	2500	0.000
	98	-9445.397	4641.883	37.083	-7922.395	0	2500	0.000	-8095.111	0	2500	0.000
	99	-13417.504	2242.219	68.096	-11646.971	0	2500	0.000	-12039.143	0	2500	0.000
	100	-9247.197	4621.883	36.276	-7703.281	0	2500	0.000	-7893.577	0	2500	0.000
**Mean**		-7825.827	3310.259	32.358	-6602.618	0.2	2500.0	0.000	-6776.819	1.6	2500.0	0.001
**Std. Err.**		203.384	138.257	0.751	167.290	0.1	0.0	0.000	170.027	0.3	0.0	0.000
Actinopteri	1	-87234.771	6926.452	36.656	-79472.244	0	2500	0.000	-79261.624	0	2500	0.000
	2	-96945.013	7250.634	40.475	-88651.362	0	2500	0.000	-88394.659	0	2500	0.000
	3	-89000.705	7042.252	37.113	-81348.886	0	2500	0.000	-81185.857	0	2500	0.000

	4	-95468.667	7730.114	39.611	-86024.425	0	2500	0.000	-86094.757	0	2500	0.000
	5	-94558.884	6936.833	39.931	-86328.844	0	2500	0.000	-85939.371	0	2500	0.000
	6	-91108.805	7095.665	37.663	-83137.428	0	2500	0.000	-83031.806	0	2500	0.000
	7	-104738.123	8433.246	42.484	-95212.983	0	2500	0.000	-95139.451	0	2500	0.000
	8	-96826.108	7252.229	40.696	-87610.282	0	2500	0.000	-87543.066	0	2500	0.000
	9	-94226.034	7564.944	38.760	-84750.482	0	2500	0.000	-84862.733	0	2500	0.000
	10	-94939.881	7484.938	39.532	-86298.007	0	2500	0.000	-86159.353	0	2500	0.000
	11	-101559.081	8195.945	42.600	-92136.473	0	2500	0.000	-92178.096	0	2500	0.000
	12	-88100.240	6504.834	36.414	-80342.679	0	2500	0.000	-80010.984	0	2500	0.000
	13	-90332.583	7619.445	37.022	-81679.304	0	2500	0.000	-81900.724	0	2500	0.000
	14	-96625.455	7396.455	40.111	-87189.707	0	2500	0.000	-87198.008	0	2500	0.000
	15	-97505.020	8540.978	39.607	-87651.665	0	2500	0.000	-87967.162	0	2500	0.000
	16	-91937.160	6837.065	38.309	-83475.207	0	2500	0.000	-83315.334	0	2500	0.000
	17	-89847.372	7800.562	36.583	-81602.257	0	2500	0.000	-81717.536	0	2500	0.000
	18	-93318.151	6769.084	39.623	-84728.284	0	2500	0.000	-84438.555	0	2500	0.000
	19	-94209.239	8072.134	38.621	-85476.788	0	2500	0.000	-85607.977	0	2500	0.000
	20	-97020.600	7559.921	41.132	-88087.287	0	2500	0.000	-88026.467	0	2500	0.000
	21	-94890.352	7532.274	39.547	-85709.936	0	2500	0.000	-85650.660	0	2500	0.000
	22	-95049.425	7822.021	39.697	-85323.834	0	2500	0.000	-85573.905	0	2500	0.000
	23	-91548.431	8359.444	36.572	-82589.456	0	2500	0.000	-82994.586	0	2500	0.000
	24	-90457.903	7107.451	37.104	-82094.480	0	2500	0.000	-82018.504	0	2500	0.000
	25	-94020.012	7394.848	39.217	-86121.478	0	2500	0.000	-85769.006	0	2500	0.000
	26	-96661.016	8756.991	38.781	-86583.188	0	2500	0.000	-87041.174	0	2500	0.000
	27	-94925.052	7415.196	39.986	-85990.546	0	2500	0.000	-86067.628	0	2500	0.000
	28	-90989.410	6710.801	39.122	-82794.522	0	2500	0.000	-82642.310	0	2500	0.000
	29	-95817.138	7994.672	39.000	-86008.796	0	2500	0.000	-86339.729	0	2500	0.000
	30	-95948.842	7921.300	39.022	-86268.532	0	2500	0.000	-86490.990	0	2500	0.000
	31	-95502.802	7144.206	40.974	-87278.538	0	2500	0.000	-86960.752	0	2500	0.000
	32	-102302.225	8531.770	42.015	-92787.564	0	2500	0.000	-92838.751	0	2500	0.000
	33	-96734.493	7346.038	40.851	-87716.969	0	2500	0.000	-87550.340	0	2500	0.000
	34	-96266.324	7742.655	40.005	-87001.019	0	2500	0.000	-86981.085	0	2500	0.000
	35	-93912.350	8480.251	37.258	-84261.082	0	2500	0.000	-84745.497	0	2500	0.000
	36	-91527.201	7144.889	37.666	-83537.904	0	2500	0.000	-83286.025	0	2500	0.000
	37	-87415.608	6276.653	36.989	-79794.736	0	2500	0.000	-79522.169	0	2500	0.000
	38	-101834.183	8173.774	42.360	-92151.056	0	2500	0.000	-92299.574	0	2500	0.000
	39	-98505.964	8783.121	40.900	-88634.003	0	2500	0.000	-88993.599	0	2500	0.000
	40	-96955.179	8089.367	39.682	-87258.427	0	2500	0.000	-87440.193	0	2500	0.000
	41	-91528.826	8462.883	37.595	-82590.777	0	2500	0.000	-82971.150	0	2500	0.000
	42	-97792.724	7820.691	40.663	-88052.317	0	2500	0.000	-88027.289	0	2500	0.000
	43	-85830.891	7462.068	35.265	-77525.784	0	2500	0.000	-77658.283	0	2500	0.000
	44	-85569.811	6317.216	35.986	-77601.258	0	2500	0.000	-77462.151	0	2500	0.000
	45	-93847.373	8285.736	38.700	-84063.018	0	2500	0.000	-84480.308	0	2500	0.000
	46	-98536.743	8787.049	40.143	-88737.077	0	2500	0.000	-89086.852	0	2500	0.000
	47	-84971.249	6195.216	35.732	-78198.605	0	2500	0.000	-77663.410	0	2500	0.000
	48	-98345.829	8070.153	40.779	-89180.710	0	2500	0.000	-89396.512	0	2500	0.000
	49	-99991.175	8394.521	40.726	-90329.044	0	2500	0.000	-90415.760	0	2500	0.000
	50	-96085.250	7032.976	40.937	-87786.768	0	2500	0.000	-87485.801	0	2500	0.000
	51	-86175.115	5886.108	37.228	-79101.127	0	2500	0.000	-78579.190	0	2500	0.000
	52	-93518.582	7410.054	39.011	-84693.175	0	2500	0.000	-84673.702	0	2500	0.000
	53	-85800.077	6846.875	35.373	-77546.067	0	2500	0.000	-77742.499	0	2500	0.000
	54	-100904.075	8464.897	41.119	-90459.599	0	2500	0.000	-90845.681	0	2500	0.000
	55	-91455.823	6713.450	38.753	-83126.197	0	2500	0.000	-82962.596	0	2500	0.000
	56	-86053.671	6222.873	36.083	-78376.088	0	2500	0.000	-78022.630	0	2500	0.000
	57	-102785.559	8731.145	42.472	-92430.046	0	2500	0.000	-92821.752	0	2500	0.000
	58	-97201.368	7953.444	39.834	-87824.685	0	2500	0.000	-87872.427	0	2500	0.000
	59	-100714.465	8827.581	41.289	-90575.999	0	2500	0.000	-90897.511	0	2500	0.000
	60	-95140.215	6541.944	40.588	-87638.840	0	2500	0.000	-86922.561	0	2500	0.000
	61	-88159.595	6532.922	37.155	-80254.853	0	2500	0.000	-80011.248	0	2500	0.000
	62	-92522.317	6321.977	39.286	-84955.768	0	2500	0.000	-84315.348	0	2500	0.000
	63	-91848.260	7690.991	37.887	-83255.930	0	2500	0.000	-83366.400	0	2500	0.000
	64	-91350.533	7210.012	37.250	-83047.671	0	2500	0.000	-82945.469	0	2500	0.000
	65	-97998.899	7349.171	40.458	-88664.180	0	2500	0.000	-88547.631	0	2500	0.000
	66	-88443.176	6565.087	37.271	-80766.578	0	2500	0.000	-80430.141	0	2500	0.000
	67	-99271.692	7871.355	41.707	-89477.673	0	2500	0.000	-89516.129	0	2500	0.000
	68	-91742.369	7437.159	37.450	-83225.415	0	2500	0.000	-83230.647	0	2500	0.000
	69	-99908.307	7926.315	40.666	-90185.081	0	2500	0.000	-90390.858	0	2500	0.000

	70	-102695.663	8685.777	42.225	-92379.309	0	2500	0.000	-92705.040	0	2500	0.000
	71	-81982.763	6859.856	34.007	-74498.100	0	2500	0.000	-74541.196	0	2500	0.000
	72	-89964.499	6866.956	37.919	-81197.797	0	2500	0.000	-81224.854	0	2500	0.000
	73	-88508.741	7109.671	36.815	-80172.670	0	2500	0.000	-80194.190	0	2500	0.000
	74	-88446.453	6216.991	37.015	-81161.231	0	2500	0.000	-80620.286	0	2500	0.000
	75	-96811.795	8432.441	40.168	-87281.185	0	2500	0.000	-87686.438	0	2500	0.000
	76	-94274.830	7114.389	39.926	-85872.277	0	2500	0.000	-85689.230	0	2500	0.000
	77	-93468.944	7827.622	38.706	-84430.029	0	2500	0.000	-84693.057	0	2500	0.000
	78	-91931.633	6696.673	38.889	-83930.136	0	2500	0.000	-83599.519	0	2500	0.000
	79	-94167.872	6983.201	39.930	-85752.536	0	2500	0.000	-85411.049	0	2500	0.000
	80	-92881.406	6806.835	38.873	-85148.379	0	2500	0.000	-84718.820	0	2500	0.000
	81	-99548.187	7807.829	41.154	-90267.459	0	2500	0.000	-90278.283	0	2500	0.000
	82	-96012.047	7783.622	39.549	-86794.815	0	2500	0.000	-86948.961	0	2500	0.000
	83	-88954.873	6743.023	37.748	-81620.380	0	2500	0.000	-81386.317	0	2500	0.000
	84	-86335.744	7002.463	36.761	-78714.487	0	2500	0.000	-78729.660	0	2500	0.000
	85	-88943.273	6899.597	37.182	-80438.831	0	2500	0.000	-80419.409	0	2500	0.000
	86	-94986.533	8062.904	38.720	-85611.175	0	2500	0.000	-85849.021	0	2500	0.000
	87	-96746.763	8167.535	39.646	-87734.009	0	2500	0.000	-87757.064	0	2500	0.000
	88	-95029.002	7782.791	39.211	-86110.724	0	2500	0.000	-86171.596	0	2500	0.000
	89	-102575.655	8562.606	42.423	-92288.555	0	2500	0.000	-92562.937	0	2500	0.000
	90	-92575.336	7892.493	38.136	-83984.051	0	2500	0.000	-84238.370	0	2500	0.000
	91	-100108.393	8422.721	40.770	-89628.697	0	2500	0.000	-89880.213	0	2500	0.000
	92	-101974.761	8000.170	42.001	-92663.699	0	2500	0.000	-92434.721	0	2500	0.000
	93	-99550.177	7828.096	41.485	-90086.548	0	2500	0.000	-89925.905	0	2500	0.000
	94	-91179.093	7433.016	37.696	-82615.452	0	2500	0.000	-82775.430	0	2500	0.000
	95	-97760.054	7630.185	40.753	-88678.318	0	2500	0.000	-88576.623	0	2500	0.000
	96	-85595.202	7116.967	35.681	-77822.850	0	2500	0.000	-77888.518	0	2500	0.000
	97	-94201.654	7649.000	38.720	-85653.827	0	2500	0.000	-85607.294	0	2500	0.000
	98	-102331.351	8289.355	42.054	-92404.157	0	2500	0.000	-92515.754	0	2500	0.000
	99	-93776.176	7140.434	39.867	-85481.680	0	2500	0.000	-85270.861	0	2500	0.000
	100	-104744.389	9096.211	42.917	-94107.992	0	2500	0.000	-94642.451	0	2500	0.000
**Mean**		-94283.230	7539.848	39.140	-85453.123	0.0	2500.0	0.000	-85448.650	0.0	2500.0	0.000
**Std. Err.**		496.721	72.882	0.195	427.007	0.0	0.0	0.000	435.523	0.0	0.0	0.000
Amphibia	1	-2645.070	1035.906	21.738	-2201.924	3	2500	0.001	-2266.757	13	2500	0.005
	2	-6385.715	3329.296	50.663	-5371.405	0	2500	0.000	-5517.658	0	2500	0.000
	3	-2259.606	1079.996	14.269	-1922.463	18	2500	0.007	-1962.361	41	2500	0.016
	4	-3933.084	2268.957	30.802	-3287.170	0	2500	0.000	-3364.407	2	2500	0.001
	5	-5509.257	2963.478	44.340	-4605.306	0	2500	0.000	-4722.995	0	2500	0.000
	6	-5136.253	3037.225	42.690	-4247.958	0	2500	0.000	-4355.729	0	2500	0.000
	7	-2132.198	918.284	17.318	-1745.840	3	2500	0.001	-1796.044	10	2500	0.004
	8	-3203.624	1441.062	26.418	-2613.858	0	2500	0.000	-2678.432	1	2500	0.000
	9	-3254.361	1814.892	28.930	-2688.284	1	2500	0.000	-2757.580	4	2500	0.002
	10	-2602.596	971.281	24.379	-2119.104	0	2500	0.000	-2187.779	4	2500	0.002
	11	-6044.024	3385.447	48.311	-5070.482	0	2500	0.000	-5198.394	0	2500	0.000
	12	-3123.907	970.456	28.017	-2601.754	2	2500	0.001	-2692.034	6	2500	0.002
	13	-6241.263	3486.819	52.659	-5154.867	0	2500	0.000	-5300.278	0	2500	0.000
	14	-6281.600	3415.870	50.716	-5241.904	0	2500	0.000	-5371.460	0	2500	0.000
	15	-2887.446	1378.340	23.973	-2384.489	4	2500	0.002	-2449.175	4	2500	0.002
	16	-3241.101	1510.578	24.066	-2681.787	4	2500	0.002	-2752.035	4	2500	0.002
	17	-4995.756	2868.934	44.148	-4081.785	0	2500	0.000	-4197.201	0	2500	0.000
	18	-3027.251	1444.442	23.534	-2495.490	1	2500	0.000	-2563.924	9	2500	0.004
	19	-1751.218	772.696	12.982	-1454.601	32	2500	0.013	-1502.209	51	2500	0.020
	20	-5875.905	3243.709	48.263	-4854.945	0	2500	0.000	-4973.620	0	2500	0.000
	21	-6202.110	3185.560	48.518	-5240.056	0	2500	0.000	-5384.532	0	2500	0.000
	22	-2179.032	1124.760	15.486	-1820.759	17	2500	0.007	-1868.938	27	2500	0.011
	23	-2426.567	1069.990	17.292	-2017.472	5	2500	0.002	-2068.283	22	2500	0.009
	24	-3483.008	2055.002	27.524	-2893.308	1	2500	0.000	-2961.411	2	2500	0.001
	25	-2599.306	1320.442	16.360	-2208.883	14	2500	0.006	-2257.270	38	2500	0.015
	26	-4435.101	2591.154	37.250	-3631.443	0	2500	0.000	-3719.216	1	2500	0.000
	27	-2463.947	1101.906	19.372	-2019.841	2	2500	0.001	-2073.544	5	2500	0.002
	28	-4598.820	2256.963	40.729	-3832.575	0	2500	0.000	-3945.238	1	2500	0.000
	29	-4755.192	2737.829	40.788	-3920.699	0	2500	0.000	-4035.554	0	2500	0.000
	30	-3084.465	1108.647	29.737	-2514.018	0	2500	0.000	-2597.703	1	2500	0.000
	31	-1497.490	693.480	11.463	-1259.902	52	2500	0.021	-1293.131	110	2500	0.044
	32	-3912.679	2173.159	33.448	-3258.575	0	2500	0.000	-3358.687	2	2500	0.001
	33	-3336.264	1623.180	24.644	-2776.141	2	2500	0.001	-2859.479	8	2500	0.003

	34	-3920.092	2254.992	33.328	-3221.725	0	2500	0.000	-3308.788	0	2500	0.000
	35	-6365.352	3321.750	52.667	-5307.970	0	2500	0.000	-5450.317	0	2500	0.000
	36	-1976.965	953.815	14.258	-1656.180	29	2500	0.012	-1699.161	49	2500	0.020
	37	-5630.385	3057.924	48.479	-4670.059	0	2500	0.000	-4797.195	1	2500	0.000
	38	-6044.024	3385.447	48.311	-5070.482	0	2500	0.000	-5198.394	0	2500	0.000
	39	-5943.076	3137.484	47.814	-4978.476	0	2500	0.000	-5114.634	0	2500	0.000
	40	-4389.148	2505.697	36.421	-3627.452	0	2500	0.000	-3717.204	0	2500	0.000
	41	-3483.008	2055.002	27.524	-2893.308	1	2500	0.000	-2961.411	2	2500	0.001
	42	-2089.473	1017.083	14.963	-1741.077	7	2500	0.003	-1779.831	32	2500	0.013
	43	-4088.621	2345.317	33.262	-3393.765	0	2500	0.000	-3492.144	0	2500	0.000
	44	-5901.989	3284.645	46.202	-4931.182	0	2500	0.000	-5053.483	0	2500	0.000
	45	-3912.679	2173.159	33.448	-3258.575	0	2500	0.000	-3358.687	2	2500	0.001
	46	-2675.367	995.793	27.109	-2168.316	0	2500	0.000	-2257.381	4	2500	0.002
	47	-3963.361	2252.663	35.430	-3279.373	0	2500	0.000	-3368.932	2	2500	0.001
	48	-2760.265	1252.223	19.329	-2297.597	3	2500	0.001	-2355.214	10	2500	0.004
	49	-3074.606	1359.749	28.114	-2496.018	0	2500	0.000	-2576.434	2	2500	0.001
	50	-2309.703	1171.090	16.324	-1931.437	16	2500	0.006	-1983.057	17	2500	0.007
	51	-3203.624	1441.062	26.418	-2613.858	0	2500	0.000	-2678.432	1	2500	0.000
	52	-5486.262	2932.543	46.409	-4518.571	0	2500	0.000	-4645.909	0	2500	0.000
	53	-3512.979	1610.208	27.518	-2889.197	0	2500	0.000	-2967.245	1	2500	0.000
	54	-6281.600	3415.870	50.716	-5241.904	0	2500	0.000	-5371.460	0	2500	0.000
	55	-3912.679	2173.159	33.448	-3258.575	0	2500	0.000	-3358.687	2	2500	0.001
	56	-2599.306	1320.442	16.360	-2208.883	14	2500	0.006	-2257.270	38	2500	0.015
	57	-4995.756	2868.934	44.148	-4081.785	0	2500	0.000	-4197.201	0	2500	0.000
	58	-1228.668	580.096	11.478	-1010.975	40	2500	0.016	-1042.398	67	2500	0.027
	59	-4460.087	2629.048	35.229	-3708.366	1	2500	0.000	-3798.866	1	2500	0.000
	60	-6726.562	3587.387	53.556	-5631.941	0	2500	0.000	-5781.105	0	2500	0.000
	61	-5076.764	2596.514	42.516	-4265.129	0	2500	0.000	-4401.772	2	2500	0.001
	62	-1976.965	953.815	14.258	-1656.180	29	2500	0.012	-1699.161	49	2500	0.020
	63	-1751.218	772.696	12.982	-1454.601	32	2500	0.013	-1502.209	51	2500	0.020
	64	-3336.264	1623.180	24.644	-2776.141	2	2500	0.001	-2859.479	8	2500	0.003
	65	-2645.070	1035.906	21.738	-2201.924	3	2500	0.001	-2266.757	13	2500	0.005
	66	-4995.756	2868.934	44.148	-4081.785	0	2500	0.000	-4197.201	0	2500	0.000
	67	-3535.301	1631.494	28.521	-2908.554	3	2500	0.001	-2988.444	0	2500	0.000
	68	-4795.207	2782.947	41.691	-3928.555	0	2500	0.000	-4036.326	0	2500	0.000
	69	-6480.011	3664.607	52.474	-5353.647	0	2500	0.000	-5480.474	0	2500	0.000
	70	-5845.240	2899.504	48.819	-4918.281	0	2500	0.000	-5052.921	0	2500	0.000
	71	-6225.490	3502.433	49.200	-5180.214	0	2500	0.000	-5304.748	0	2500	0.000
	72	-2760.265	1252.223	19.329	-2297.597	3	2500	0.001	-2355.214	10	2500	0.004
	73	-1475.130	656.712	13.002	-1204.364	19	2500	0.008	-1238.135	38	2500	0.015
	74	-5486.262	2932.543	46.409	-4518.571	0	2500	0.000	-4645.909	0	2500	0.000
	75	-4598.820	2256.963	40.729	-3832.575	0	2500	0.000	-3945.238	1	2500	0.000
	76	-2189.178	845.085	16.748	-1831.112	12	2500	0.005	-1883.278	30	2500	0.012
	77	-4063.144	2310.805	35.375	-3312.383	0	2500	0.000	-3405.135	0	2500	0.000
	78	-1475.130	656.712	13.002	-1204.364	19	2500	0.008	-1238.135	38	2500	0.015
	79	-3312.734	986.292	32.410	-2727.538	0	2500	0.000	-2825.241	3	2500	0.001
	80	-6044.024	3385.447	48.311	-5070.482	0	2500	0.000	-5198.394	0	2500	0.000
	81	-4995.756	2868.934	44.148	-4081.785	0	2500	0.000	-4197.201	0	2500	0.000
	82	-2488.513	1092.919	22.564	-2011.058	0	2500	0.000	-2078.640	4	2500	0.002
	83	-5901.989	3284.645	46.202	-4931.182	0	2500	0.000	-5053.483	0	2500	0.000
	84	-1475.130	656.712	13.002	-1204.364	19	2500	0.008	-1238.135	38	2500	0.015
	85	-2259.606	1079.996	14.269	-1922.463	18	2500	0.007	-1962.361	41	2500	0.016
	86	-5133.553	2692.403	44.446	-4288.486	0	2500	0.000	-4419.383	2	2500	0.001
	87	-1556.914	825.089	13.374	-1280.466	22	2500	0.009	-1317.291	34	2500	0.014
	88	-1228.668	580.096	11.478	-1010.975	40	2500	0.016	-1042.398	67	2500	0.027
	89	-3027.251	1444.442	23.534	-2495.490	1	2500	0.000	-2563.924	9	2500	0.004
	90	-6675.930	3716.390	52.601	-5577.127	0	2500	0.000	-5722.794	0	2500	0.000
	91	-4732.708	2337.096	42.877	-3951.244	0	2500	0.000	-4082.401	1	2500	0.000
	92	-6202.110	3185.560	48.518	-5240.056	0	2500	0.000	-5384.532	0	2500	0.000
	93	-2815.336	1593.379	25.659	-2309.138	2	2500	0.001	-2368.500	2	2500	0.001
	94	-4088.621	2345.317	33.262	-3393.765	0	2500	0.000	-3492.144	0	2500	0.000
	95	-3241.101	1510.578	24.066	-2681.787	4	2500	0.002	-2752.035	4	2500	0.002
	96	-5438.919	2554.577	47.266	-4573.335	0	2500	0.000	-4713.969	0	2500	0.000
	97	-5943.076	3137.484	47.814	-4978.476	0	2500	0.000	-5114.634	0	2500	0.000
	98	-2789.421	1253.604	23.603	-2300.454	2	2500	0.001	-2365.954	9	2500	0.004
	99	-6675.930	3716.390	52.601	-5577.127	0	2500	0.000	-5722.794	0	2500	0.000

	100	-3350.064	1378.281	29.687	-2711.122	0	2500	0.000	-2795.520	1	2500	0.000
**Mean**		-3945.294	2043.310	32.364	-3275.500	5.0	2500.0	0.002	-3365.162	10.5	2500.0	0.004
**Std. Err.**		158.521	96.289	1.331	132.784	1.0	0.0	0.000	136.210	1.9	0.0	0.001
Sauropsida	1	-12962.411	5322.905	20.348	-11011.572	0	2500	0.000	-11214.688	0	2500	0.000
	2	-11864.336	4882.154	19.109	-10017.774	0	2500	0.000	-10211.832	0	2500	0.000
	3	-10700.774	4167.598	16.311	-9151.874	0	2500	0.000	-9343.310	2	2500	0.001
	4	-7754.670	3316.288	11.695	-6615.466	0	2500	0.000	-6742.615	3	2500	0.001
	5	-13267.737	5755.744	20.456	-11279.198	0	2500	0.000	-11475.520	0	2500	0.000
	6	-12494.283	4779.873	18.569	-10658.792	0	2500	0.000	-10870.582	0	2500	0.000
	7	-9702.313	3593.629	14.708	-8158.402	0	2500	0.000	-8347.470	0	2500	0.000
	8	-9589.799	3980.841	14.218	-8231.018	0	2500	0.000	-8384.598	0	2500	0.000
	9	-13480.855	4611.074	21.198	-11386.516	0	2500	0.000	-11634.027	0	2500	0.000
	10	-11260.258	3709.564	17.416	-9545.252	0	2500	0.000	-9742.683	0	2500	0.000
	11	-11445.652	4914.197	18.158	-9601.347	0	2500	0.000	-9785.580	0	2500	0.000
	12	-14202.234	6093.438	21.683	-11993.653	0	2500	0.000	-12216.376	0	2500	0.000
	13	-12041.252	4933.263	18.603	-10292.248	0	2500	0.000	-10501.315	0	2500	0.000
	14	-9946.589	4046.542	15.473	-8368.294	0	2500	0.000	-8554.058	0	2500	0.000
	15	-13310.733	5731.423	20.283	-11390.773	0	2500	0.000	-11592.152	0	2500	0.000
	16	-15818.685	6413.524	24.492	-13536.403	0	2500	0.000	-13811.543	0	2500	0.000
	17	-11443.259	3473.192	18.491	-9833.818	0	2500	0.000	-10068.193	0	2500	0.000
	18	-10448.532	4258.029	16.008	-8850.837	0	2500	0.000	-9030.360	0	2500	0.000
	19	-7330.698	3633.896	10.427	-6090.666	0	2500	0.000	-6197.816	0	2500	0.000
	20	-13387.672	5534.122	20.116	-11321.205	0	2500	0.000	-11539.490	0	2500	0.000
	21	-15507.386	5311.792	25.017	-13251.764	0	2500	0.000	-13549.232	0	2500	0.000
	22	-11449.186	5513.147	16.325	-9817.389	0	2500	0.000	-10000.334	0	2500	0.000
	23	-10900.917	5729.525	16.394	-9160.936	0	2500	0.000	-9336.248	0	2500	0.000
	24	-13285.694	4399.631	22.647	-11256.139	0	2500	0.000	-11525.141	0	2500	0.000
	25	-14304.901	6282.607	21.409	-12189.135	0	2500	0.000	-12433.062	0	2500	0.000
	26	-15798.017	6425.495	25.624	-13450.872	0	2500	0.000	-13730.531	0	2500	0.000
	27	-10754.118	4515.725	16.668	-9116.011	0	2500	0.000	-9303.838	1	2500	0.000
	28	-8396.419	3559.386	11.320	-7040.726	0	2500	0.000	-7170.141	1	2500	0.000
	29	-11556.003	4162.959	19.280	-9777.737	0	2500	0.000	-10000.508	0	2500	0.000
	30	-10704.013	4132.338	16.776	-9151.244	0	2500	0.000	-9335.506	0	2500	0.000
	31	-14584.021	6129.952	22.536	-12396.660	0	2500	0.000	-12675.441	0	2500	0.000
	32	-12271.081	4779.310	20.787	-10406.140	0	2500	0.000	-10635.830	0	2500	0.000
	33	-15067.993	6905.779	24.300	-12722.300	0	2500	0.000	-12946.010	0	2500	0.000
	34	-10543.041	4499.415	16.854	-8962.803	0	2500	0.000	-9135.355	0	2500	0.000
	35	-12481.157	4776.980	19.929	-10651.169	0	2500	0.000	-10871.856	0	2500	0.000
	36	-10893.856	3231.428	18.528	-9347.276	0	2500	0.000	-9568.628	0	2500	0.000
	37	-10263.613	4276.577	15.546	-8842.865	0	2500	0.000	-9006.186	0	2500	0.000
	38	-12446.403	5247.032	18.220	-10575.953	0	2500	0.000	-10779.638	0	2500	0.000
	39	-10792.560	4264.887	16.826	-9225.594	0	2500	0.000	-9414.581	0	2500	0.000
	40	-13725.913	6919.464	21.021	-11666.380	0	2500	0.000	-11877.254	0	2500	0.000
	41	-12303.849	5853.950	19.740	-10433.901	0	2500	0.000	-10615.613	0	2500	0.000
	42	-13303.076	4050.732	21.448	-11379.848	0	2500	0.000	-11616.679	0	2500	0.000
	43	-12670.238	5104.195	19.083	-10746.064	0	2500	0.000	-10938.903	0	2500	0.000
	44	-9880.695	4178.609	15.443	-8431.228	0	2500	0.000	-8594.387	1	2500	0.000
	45	-12044.747	5010.342	18.448	-10125.410	0	2500	0.000	-10338.318	0	2500	0.000
	46	-9702.775	4097.174	14.773	-8225.667	1	2500	0.000	-8406.985	0	2500	0.000
	47	-11408.593	3950.772	18.253	-9726.394	0	2500	0.000	-9937.882	0	2500	0.000
	48	-10023.995	3152.637	16.828	-8476.212	0	2500	0.000	-8675.881	0	2500	0.000
	49	-11786.256	4825.527	18.485	-9992.704	0	2500	0.000	-10187.324	0	2500	0.000
	50	-9185.002	3743.249	13.693	-7892.348	0	2500	0.000	-8044.358	1	2500	0.000
	51	-11659.076	4629.814	19.517	-9894.708	0	2500	0.000	-10113.357	0	2500	0.000
	52	-12894.502	3979.895	21.228	-11141.701	0	2500	0.000	-11379.872	0	2500	0.000
	53	-8994.641	4045.910	12.902	-7591.514	0	2500	0.000	-7711.456	0	2500	0.000
	54	-12379.287	5224.629	17.623	-10513.052	0	2500	0.000	-10684.694	0	2500	0.000
	55	-12758.129	3717.275	23.270	-10875.490	0	2500	0.000	-11141.375	0	2500	0.000
	56	-13042.051	5708.491	19.983	-11084.114	0	2500	0.000	-11302.428	0	2500	0.000
	57	-7556.419	3258.083	10.648	-6450.020	1	2500	0.000	-6561.395	2	2500	0.001
	58	-11845.441	5638.124	17.793	-10024.072	0	2500	0.000	-10220.625	0	2500	0.000
	59	-10628.496	4420.279	16.412	-8987.085	0	2500	0.000	-9168.872	0	2500	0.000
	60	-7706.912	3281.767	10.794	-6605.132	1	2500	0.000	-6746.969	3	2500	0.001
	61	-12977.327	5578.749	19.938	-11243.900	0	2500	0.000	-11459.930	0	2500	0.000
	62	-14141.482	6227.766	22.316	-12062.048	0	2500	0.000	-12291.710	0	2500	0.000
	63	-15850.988	6390.527	24.540	-13545.695	0	2500	0.000	-13809.856	0	2500	0.000

	64	-11299.944	5694.572	17.566	-9441.170	0	2500	0.000	-9611.271	0	2500	0.000
	65	-12085.845	5147.787	20.445	-10261.681	0	2500	0.000	-10471.768	0	2500	0.000
	66	-14888.275	5075.286	25.060	-12626.209	0	2500	0.000	-12943.802	0	2500	0.000
	67	-9851.993	2877.152	16.196	-8434.684	0	2500	0.000	-8608.743	0	2500	0.000
	68	-13233.946	5713.601	20.054	-11391.649	0	2500	0.000	-11621.291	0	2500	0.000
	69	-11818.786	5719.800	19.014	-9939.560	0	2500	0.000	-10112.616	0	2500	0.000
	70	-10725.417	4903.524	17.762	-9081.384	0	2500	0.000	-9248.587	0	2500	0.000
	71	-7952.232	3323.997	12.419	-6713.010	0	2500	0.000	-6841.015	0	2500	0.000
	72	-11520.249	3943.477	19.579	-9709.184	0	2500	0.000	-9939.107	0	2500	0.000
	73	-12438.779	5357.882	18.418	-10540.678	0	2500	0.000	-10755.974	0	2500	0.000
	74	-10989.040	4871.044	16.661	-9297.789	0	2500	0.000	-9489.597	0	2500	0.000
	75	-8465.206	3792.726	11.703	-7099.861	0	2500	0.000	-7226.586	0	2500	0.000
	76	-13957.925	4892.425	22.688	-11945.115	0	2500	0.000	-12210.230	0	2500	0.000
	77	-13609.852	5246.993	22.271	-11600.326	0	2500	0.000	-11868.194	0	2500	0.000
	78	-11447.400	4980.767	18.166	-9703.712	0	2500	0.000	-9904.889	0	2500	0.000
	79	-15610.638	6565.767	24.185	-13324.556	0	2500	0.000	-13577.560	0	2500	0.000
	80	-11704.533	4830.575	19.014	-9796.409	0	2500	0.000	-9986.984	0	2500	0.000
	81	-9683.063	4096.450	14.422	-8291.724	0	2500	0.000	-8439.942	0	2500	0.000
	82	-10311.800	3313.429	17.856	-8711.407	0	2500	0.000	-8922.485	0	2500	0.000
	83	-9491.568	3206.014	14.634	-8026.106	0	2500	0.000	-8197.367	0	2500	0.000
	84	-12158.914	4739.073	18.126	-10339.417	0	2500	0.000	-10557.534	0	2500	0.000
	85	-9251.650	3356.257	13.931	-7846.929	0	2500	0.000	-8013.448	0	2500	0.000
	86	-9599.346	3809.063	15.451	-8165.734	0	2500	0.000	-8345.092	0	2500	0.000
	87	-15417.485	6133.918	24.383	-13086.487	0	2500	0.000	-13354.235	0	2500	0.000
	88	-15016.104	5863.509	23.243	-12764.054	0	2500	0.000	-13027.353	0	2500	0.000
	89	-10670.490	2934.367	17.378	-9088.666	0	2500	0.000	-9315.468	0	2500	0.000
	90	-10033.470	4088.096	15.686	-8592.967	0	2500	0.000	-8765.172	0	2500	0.000
	91	-11013.263	5085.313	16.497	-9311.542	0	2500	0.000	-9509.453	0	2500	0.000
	92	-11230.993	5643.154	16.675	-9543.978	0	2500	0.000	-9709.720	0	2500	0.000
	93	-10620.435	4055.901	17.136	-8897.832	0	2500	0.000	-9096.529	0	2500	0.000
	94	-9570.535	3151.612	14.710	-8242.830	0	2500	0.000	-8421.340	1	2500	0.000
	95	-14459.037	6252.176	22.676	-12164.855	0	2500	0.000	-12415.909	0	2500	0.000
	96	-12368.791	5097.583	18.629	-10409.122	0	2500	0.000	-10615.182	0	2500	0.000
	97	-10383.575	3748.741	16.485	-8837.909	0	2500	0.000	-9047.896	0	2500	0.000
	98	-15569.234	6669.494	25.900	-13206.582	0	2500	0.000	-13466.085	0	2500	0.000
	99	-9870.503	4239.952	15.333	-8256.638	0	2500	0.000	-8428.277	0	2500	0.000
	100	-12607.208	5734.482	19.420	-10790.874	0	2500	0.000	-10996.587	0	2500	0.000
**Mean**		-11738.505	4744.392	18.367	-9972.751	0.0	2500.0	0.000	-10175.717	0.2	2500.0	0.000
**Std. Err.**		204.483	101.648	0.355	175.096	0.0	0.0	0.000	178.742	0.1	0.0	0.000
Aves	1	-34865.996	8015.504	15.825	-29572.531	0	2500	0.000	-30152.317	0	2500	0.000
	2	-37583.435	8509.847	17.454	-31771.865	0	2500	0.000	-32410.100	0	2500	0.000
	3	-33220.121	8117.768	15.271	-28146.511	0	2500	0.000	-28740.639	0	2500	0.000
	4	-31764.846	8109.649	14.324	-26936.832	0	2500	0.000	-27492.176	0	2500	0.000
	5	-36324.108	9751.346	16.630	-30853.181	0	2500	0.000	-31452.473	0	2500	0.000
	6	-37826.599	9085.395	17.123	-32052.415	0	2500	0.000	-32691.406	0	2500	0.000
	7	-34513.815	7975.494	15.724	-29291.385	0	2500	0.000	-29887.386	0	2500	0.000
	8	-37040.481	8102.449	16.992	-31232.766	0	2500	0.000	-31894.390	0	2500	0.000
	9	-37117.838	8289.144	17.007	-31466.113	0	2500	0.000	-32065.417	0	2500	0.000
	10	-36120.476	7573.456	16.534	-30596.292	0	2500	0.000	-31228.426	0	2500	0.000
	11	-38298.111	8531.213	17.657	-32550.817	0	2500	0.000	-33186.824	0	2500	0.000
	12	-34411.257	7751.897	15.645	-29215.523	0	2500	0.000	-29771.520	0	2500	0.000
	13	-39624.870	9217.196	18.106	-33545.771	0	2500	0.000	-34248.233	0	2500	0.000
	14	-38552.671	8651.328	17.491	-32705.257	0	2500	0.000	-33341.666	0	2500	0.000
	15	-36023.043	9482.623	16.387	-30407.548	0	2500	0.000	-30996.607	0	2500	0.000
	16	-31161.313	7039.179	14.174	-26284.350	0	2500	0.000	-26850.815	0	2500	0.000
	17	-34483.088	8761.407	15.880	-29213.568	0	2500	0.000	-29796.827	0	2500	0.000
	18	-36800.009	8128.337	16.867	-31186.672	0	2500	0.000	-31814.579	0	2500	0.000
	19	-39086.648	8477.969	17.803	-33130.456	0	2500	0.000	-33773.115	0	2500	0.000
	20	-35633.431	8632.761	16.154	-30137.248	0	2500	0.000	-30736.307	0	2500	0.000
	21	-35644.608	8351.535	16.330	-30112.299	0	2500	0.000	-30738.633	0	2500	0.000
	22	-38802.867	8977.020	17.616	-32755.590	0	2500	0.000	-33403.129	0	2500	0.000
	23	-36956.934	8500.845	16.737	-31316.611	0	2500	0.000	-31935.110	0	2500	0.000
	24	-40302.500	9109.506	18.356	-34170.503	0	2500	0.000	-34845.178	0	2500	0.000
	25	-35881.709	7905.138	16.669	-30368.142	0	2500	0.000	-30953.346	0	2500	0.000
	26	-33890.990	7568.130	15.418	-28914.162	0	2500	0.000	-29487.174	0	2500	0.000
	27	-37097.770	9187.429	16.979	-31407.299	0	2500	0.000	-32006.988	0	2500	0.000

	28	-31782.716	7858.864	14.584	-26956.455	0	2500	0.000	-27519.085	0	2500	0.000
	29	-33638.510	8653.063	15.425	-28345.065	0	2500	0.000	-28920.738	0	2500	0.000
	30	-36842.567	9001.594	16.930	-31256.090	0	2500	0.000	-31843.457	0	2500	0.000
	31	-32803.348	8023.974	14.806	-27918.735	0	2500	0.000	-28457.596	0	2500	0.000
	32	-32391.450	8654.215	14.867	-27351.431	0	2500	0.000	-27927.712	0	2500	0.000
	33	-35768.420	8506.481	16.531	-30258.202	0	2500	0.000	-30871.549	0	2500	0.000
	34	-38948.474	9239.277	17.902	-33022.558	0	2500	0.000	-33682.819	0	2500	0.000
	35	-38988.385	8892.276	17.993	-33025.732	0	2500	0.000	-33692.478	0	2500	0.000
	36	-33154.385	7755.331	14.994	-28073.999	0	2500	0.000	-28641.289	0	2500	0.000
	37	-33293.385	7393.908	15.250	-28242.163	0	2500	0.000	-28814.416	0	2500	0.000
	38	-34800.587	7756.445	15.936	-29414.611	0	2500	0.000	-30045.908	0	2500	0.000
	39	-35301.295	8830.731	16.141	-29783.408	0	2500	0.000	-30363.633	0	2500	0.000
	40	-34721.879	8272.152	16.061	-29403.147	0	2500	0.000	-30002.148	0	2500	0.000
	41	-36139.492	8342.362	16.624	-30531.779	0	2500	0.000	-31148.887	0	2500	0.000
	42	-32163.514	8046.914	14.742	-27121.608	0	2500	0.000	-27674.469	0	2500	0.000
	43	-36683.289	9100.814	16.631	-31119.663	0	2500	0.000	-31731.596	0	2500	0.000
	44	-35360.460	7893.421	16.096	-29969.854	0	2500	0.000	-30588.549	0	2500	0.000
	45	-30249.586	7210.596	13.938	-25815.309	0	2500	0.000	-26336.481	0	2500	0.000
	46	-34046.365	7772.588	15.540	-28865.102	0	2500	0.000	-29443.710	0	2500	0.000
	47	-35833.615	8554.746	16.401	-30421.715	0	2500	0.000	-31036.347	0	2500	0.000
	48	-34206.881	8683.289	15.501	-28974.054	0	2500	0.000	-29526.766	0	2500	0.000
	49	-34315.850	8085.098	15.611	-29059.992	0	2500	0.000	-29661.443	0	2500	0.000
	50	-36722.340	7682.432	16.775	-31117.824	0	2500	0.000	-31718.418	0	2500	0.000
	51	-32864.642	8739.640	15.028	-27833.991	0	2500	0.000	-28377.722	0	2500	0.000
	52	-36309.313	9468.447	16.585	-30900.454	0	2500	0.000	-31533.173	0	2500	0.000
	53	-40853.518	9888.513	18.690	-34595.680	0	2500	0.000	-35282.969	0	2500	0.000
	54	-36243.114	9838.222	16.808	-30766.364	0	2500	0.000	-31394.967	0	2500	0.000
	55	-38405.709	8423.365	17.511	-32395.725	0	2500	0.000	-33017.066	0	2500	0.000
	56	-33190.774	7270.372	15.170	-28079.897	0	2500	0.000	-28650.372	0	2500	0.000
	57	-32675.998	8507.435	14.978	-27518.758	0	2500	0.000	-28071.465	0	2500	0.000
	58	-33381.242	7495.008	15.121	-28327.069	0	2500	0.000	-28880.561	0	2500	0.000
	59	-35184.422	8417.263	16.156	-29752.792	0	2500	0.000	-30364.173	0	2500	0.000
	60	-32608.238	7326.940	14.755	-27692.530	0	2500	0.000	-28260.308	0	2500	0.000
	61	-35338.538	7237.441	16.271	-30020.869	0	2500	0.000	-30600.733	0	2500	0.000
	62	-35160.975	7430.500	16.257	-29781.479	0	2500	0.000	-30379.421	0	2500	0.000
	63	-33774.414	8088.281	15.485	-28642.072	0	2500	0.000	-29215.206	0	2500	0.000
	64	-36833.897	7419.103	16.810	-31333.633	0	2500	0.000	-31941.693	0	2500	0.000
	65	-34985.152	8532.836	15.930	-29755.693	0	2500	0.000	-30368.340	0	2500	0.000
	66	-34286.117	7373.564	15.608	-29070.308	0	2500	0.000	-29641.463	0	2500	0.000
	67	-34926.362	8209.850	15.810	-29632.811	0	2500	0.000	-30254.280	0	2500	0.000
	68	-34560.591	7600.576	16.093	-29245.465	0	2500	0.000	-29846.425	0	2500	0.000
	69	-38180.805	7715.100	17.295	-32302.837	0	2500	0.000	-32978.686	0	2500	0.000
	70	-37289.160	9080.674	17.139	-31592.338	0	2500	0.000	-32227.417	0	2500	0.000
	71	-35471.627	8483.239	16.476	-30088.544	0	2500	0.000	-30679.051	0	2500	0.000
	72	-32638.487	7952.719	14.997	-27540.393	0	2500	0.000	-28111.843	0	2500	0.000
	73	-36761.496	7908.564	16.771	-31145.453	0	2500	0.000	-31788.033	0	2500	0.000
	74	-35585.371	9360.768	16.367	-29942.957	0	2500	0.000	-30520.761	0	2500	0.000
	75	-31722.920	6884.253	14.623	-26805.605	0	2500	0.000	-27371.086	0	2500	0.000
	76	-37403.672	8951.708	17.150	-31705.004	0	2500	0.000	-32348.793	0	2500	0.000
	77	-32248.085	7317.979	14.782	-27370.485	0	2500	0.000	-27944.346	0	2500	0.000
	78	-38091.193	9068.251	17.453	-32335.662	0	2500	0.000	-33020.508	0	2500	0.000
	79	-39412.344	8983.705	17.993	-33331.005	0	2500	0.000	-33950.168	0	2500	0.000
	80	-32572.122	7781.231	14.788	-27604.454	0	2500	0.000	-28136.030	0	2500	0.000
	81	-38762.360	9601.181	17.632	-32768.610	0	2500	0.000	-33445.429	0	2500	0.000
	82	-35011.645	8067.814	16.105	-29675.264	0	2500	0.000	-30310.771	0	2500	0.000
	83	-37997.938	8678.927	17.236	-32218.627	0	2500	0.000	-32869.897	0	2500	0.000
	84	-37751.578	8138.958	17.289	-31883.172	0	2500	0.000	-32540.067	0	2500	0.000
	85	-38247.821	8865.308	17.462	-32460.199	0	2500	0.000	-33079.674	0	2500	0.000
	86	-32600.917	7806.687	14.841	-27695.044	0	2500	0.000	-28243.602	0	2500	0.000
	87	-34348.999	8390.120	15.739	-29010.681	0	2500	0.000	-29578.229	0	2500	0.000
	88	-34479.519	8272.529	15.993	-29268.907	0	2500	0.000	-29856.306	0	2500	0.000
	89	-32858.884	8024.183	14.971	-27924.785	0	2500	0.000	-28472.445	0	2500	0.000
	90	-35292.421	8000.068	16.056	-30133.284	0	2500	0.000	-30700.526	0	2500	0.000
	91	-34252.900	8446.774	15.536	-28946.842	0	2500	0.000	-29535.753	0	2500	0.000
	92	-32745.293	8724.329	15.030	-27576.301	0	2500	0.000	-28127.504	0	2500	0.000
	93	-34733.447	8147.429	16.062	-29486.046	0	2500	0.000	-30092.265	0	2500	0.000

	94	-35425.359	8222.194	16.252	-30021.433	0	2500	0.000	-30618.858	0	2500	0.000
	95	-37606.544	8200.226	17.376	-31843.837	0	2500	0.000	-32485.781	0	2500	0.000
	96	-35827.461	7543.195	16.580	-30401.233	0	2500	0.000	-31027.778	0	2500	0.000
	97	-30610.279	7353.275	13.984	-25997.114	0	2500	0.000	-26514.749	0	2500	0.000
	98	-34847.780	8865.370	15.773	-29410.225	0	2500	0.000	-29953.078	0	2500	0.000
	99	-36669.387	8722.080	16.559	-31155.104	0	2500	0.000	-31770.040	0	2500	0.000
	100	-34386.489	7679.019	15.504	-29129.380	0	2500	0.000	-29744.466	0	2500	0.000
**Mean**		-35406.036	8305.193	16.193	-29995.066	0.0	2500.0	0.000	-30596.746	0.0	2500.0	0.000
**Std. Err.**		227.688	66.301	0.105	192.746	0.0	0.0	0.000	196.172	0.0	0.0	0.000
Mammalia	1	-206103.290	64500.162	26.087	-177340.184	0	2500	0.000	-180136.975	0	2500	0.000
	2	-210963.155	65107.756	26.941	-181494.494	0	2500	0.000	-184329.369	0	2500	0.000
	3	-202253.757	58571.524	25.796	-173865.322	0	2500	0.000	-176726.665	0	2500	0.000
	4	-210027.347	64652.840	26.586	-180624.721	0	2500	0.000	-183487.255	0	2500	0.000
	5	-207414.693	64116.759	26.790	-178088.400	0	2500	0.000	-180934.819	0	2500	0.000
	6	-194473.117	61410.226	25.209	-167564.772	0	2500	0.000	-170214.167	0	2500	0.000
	7	-193598.200	56015.542	24.542	-166836.933	0	2500	0.000	-169530.068	0	2500	0.000
	8	-210609.700	68828.979	26.947	-180568.261	0	2500	0.000	-183411.006	0	2500	0.000
	9	-211966.722	62400.097	27.342	-182395.019	0	2500	0.000	-185334.249	0	2500	0.000
	10	-199495.899	65214.371	25.570	-171448.069	0	2500	0.000	-174094.286	0	2500	0.000
	11	-191949.878	60065.842	24.075	-164903.111	0	2500	0.000	-167572.293	0	2500	0.000
	12	-211801.776	62860.896	27.409	-182374.882	0	2500	0.000	-185349.665	0	2500	0.000
	13	-206578.876	62135.927	26.443	-177791.912	0	2500	0.000	-180585.956	0	2500	0.000
	14	-213154.352	65225.397	27.606	-183548.413	0	2500	0.000	-186545.002	0	2500	0.000
	15	-205172.770	61451.144	26.798	-176799.603	0	2500	0.000	-179717.092	0	2500	0.000
	16	-207814.064	62551.589	26.896	-178954.812	0	2500	0.000	-181843.138	0	2500	0.000
	17	-210982.782	67498.293	27.278	-181339.488	0	2500	0.000	-184183.244	0	2500	0.000
	18	-195384.669	61585.995	24.677	-168233.821	0	2500	0.000	-170866.221	0	2500	0.000
	19	-200725.229	60955.320	25.501	-173085.023	0	2500	0.000	-175745.709	0	2500	0.000
	20	-213999.845	62398.826	27.895	-184705.348	0	2500	0.000	-187542.534	0	2500	0.000
	21	-204135.543	59609.201	26.411	-175411.400	0	2500	0.000	-178316.505	0	2500	0.000
	22	-201445.385	62181.363	25.610	-173569.058	0	2500	0.000	-176349.571	0	2500	0.000
	23	-202137.159	56155.955	26.021	-174192.424	0	2500	0.000	-177099.128	0	2500	0.000
	24	-209180.609	62856.453	26.686	-179748.919	0	2500	0.000	-182565.649	0	2500	0.000
	25	-197746.287	60062.275	25.092	-170407.755	0	2500	0.000	-173119.967	0	2500	0.000
	26	-212574.442	62439.833	26.792	-182915.320	0	2500	0.000	-185767.202	0	2500	0.000
	27	-214312.030	62992.940	27.623	-184803.253	0	2500	0.000	-187776.912	0	2500	0.000
	28	-197574.340	58027.596	25.031	-170459.596	0	2500	0.000	-173181.155	0	2500	0.000
	29	-188885.017	53525.461	23.990	-162663.805	0	2500	0.000	-165294.061	0	2500	0.000
	30	-216302.236	65455.610	28.096	-186271.623	0	2500	0.000	-189295.697	0	2500	0.000
	31	-202740.958	63785.529	25.933	-174623.464	0	2500	0.000	-177390.919	0	2500	0.000
	32	-196895.334	57017.308	25.244	-169998.171	0	2500	0.000	-172731.188	0	2500	0.000
	33	-213589.584	65483.384	28.131	-183509.821	0	2500	0.000	-186524.951	0	2500	0.000
	34	-214075.691	68774.243	27.620	-183871.009	0	2500	0.000	-186760.238	0	2500	0.000
	35	-196484.944	57791.504	24.596	-169837.779	0	2500	0.000	-172466.039	0	2500	0.000
	36	-207515.323	65448.006	26.678	-178441.930	0	2500	0.000	-181256.616	0	2500	0.000
	37	-198597.684	60606.765	24.671	-170731.179	0	2500	0.000	-173357.776	0	2500	0.000
	38	-187944.033	56955.118	23.611	-161824.792	0	2500	0.000	-164353.123	0	2500	0.000
	39	-214452.795	65925.568	27.808	-184685.638	0	2500	0.000	-187625.559	0	2500	0.000
	40	-224957.715	73814.448	29.345	-193149.382	0	2500	0.000	-196193.900	0	2500	0.000
	41	-204395.413	60040.450	26.103	-175953.452	0	2500	0.000	-178760.939	0	2500	0.000
	42	-204003.922	57061.565	26.222	-175867.491	0	2500	0.000	-178761.679	0	2500	0.000
	43	-194652.486	59726.825	24.821	-167914.964	0	2500	0.000	-170591.149	0	2500	0.000
	44	-201292.505	59526.839	25.857	-172818.278	0	2500	0.000	-175626.690	0	2500	0.000
	45	-206352.432	60326.657	26.498	-177782.884	0	2500	0.000	-180678.168	0	2500	0.000
	46	-204635.979	60070.788	25.928	-176452.361	0	2091	0.000	-179206.282	0	2091	0.000
	47	-195941.516	57203.653	24.937	-168653.641	0	2500	0.000	-171348.403	0	2500	0.000
	48	-208455.203	64941.942	26.273	-178984.667	0	2500	0.000	-181808.827	0	2500	0.000
	49	-205196.411	60769.022	26.437	-176453.732	0	2500	0.000	-179308.183	0	2500	0.000
	50	-212511.591	64101.207	27.296	-182767.060	0	2500	0.000	-185738.988	0	2500	0.000
	51	-202327.257	59311.833	25.958	-174013.735	0	2500	0.000	-176953.730	0	2500	0.000
	52	-203280.436	62470.905	26.210	-175031.265	0	2500	0.000	-177687.774	0	2500	0.000
	53	-210142.530	62439.285	26.853	-180691.634	0	2500	0.000	-183536.483	0	2500	0.000
	54	-202293.391	59125.770	25.713	-173976.986	0	2500	0.000	-176705.037	0	2500	0.000
	55	-204662.802	61963.677	25.904	-175864.737	0	2500	0.000	-178687.283	0	2500	0.000
	56	-198298.324	59554.660	25.026	-171151.978	0	2500	0.000	-173839.604	0	2500	0.000

	57	-205074.358	59398.227	26.324	-176971.182	0	2500	0.000	-179843.212	0	2500	0.000
	58	-197192.466	57554.248	25.144	-169986.207	0	2500	0.000	-172695.004	0	2500	0.000
	59	-207791.702	62678.883	27.110	-178477.669	0	2500	0.000	-181387.919	0	2500	0.000
	60	-195382.357	58903.531	24.893	-168181.366	0	2500	0.000	-170876.910	0	2500	0.000
	61	-196452.034	61450.095	24.733	-169162.424	0	2500	0.000	-171843.105	0	2500	0.000
	62	-206258.440	66185.602	26.395	-176992.744	0	2500	0.000	-179830.133	0	2500	0.000
	63	-199178.670	56379.650	25.446	-171358.565	0	2500	0.000	-174198.594	0	2500	0.000
	64	-201153.848	59332.026	25.900	-173494.500	0	2500	0.000	-176287.799	0	2500	0.000
	65	-187607.523	57348.719	23.354	-161587.879	0	2500	0.000	-164171.351	0	2500	0.000
	66	-206978.625	60244.413	26.368	-178177.628	0	2500	0.000	-181084.246	0	2500	0.000
	67	-195071.174	60367.373	24.619	-168009.135	0	2500	0.000	-170607.895	0	2500	0.000
	68	-212644.066	63359.087	27.849	-182899.563	0	2500	0.000	-185864.513	0	2500	0.000
	69	-204349.567	62714.347	26.360	-175630.854	0	2500	0.000	-178371.534	0	2500	0.000
	70	-213442.905	65302.462	27.172	-184051.170	0	2500	0.000	-186940.791	0	2500	0.000
	71	-207126.907	65037.405	26.612	-177986.583	0	2500	0.000	-180760.090	0	2500	0.000
	72	-205388.717	67684.863	26.398	-176479.089	0	2500	0.000	-179220.838	0	2500	0.000
	73	-195190.530	54880.764	24.889	-168315.140	0	2500	0.000	-171041.841	0	2500	0.000
	74	-211835.308	63881.692	27.209	-182464.277	0	2500	0.000	-185471.158	0	2500	0.000
	75	-199051.611	58847.415	25.442	-171076.624	0	2500	0.000	-173951.033	0	2500	0.000
	76	-207246.411	59427.346	26.661	-178819.267	0	2500	0.000	-181692.404	0	2500	0.000
	77	-185776.031	53080.325	23.731	-160264.537	0	2500	0.000	-162866.317	0	2500	0.000
	78	-204042.249	60983.931	26.594	-175598.305	0	2500	0.000	-178358.589	0	2500	0.000
	79	-202135.003	58282.592	26.100	-173891.719	0	2500	0.000	-176724.060	0	2500	0.000
	80	-209841.060	63558.814	27.096	-180669.698	0	2500	0.000	-183534.886	0	2500	0.000
	81	-197307.807	56182.785	25.590	-170354.165	0	2500	0.000	-173142.417	0	2500	0.000
	82	-200105.875	61172.282	25.247	-172235.495	0	2500	0.000	-174972.545	0	2500	0.000
	83	-195536.752	61722.021	24.879	-168118.729	0	2500	0.000	-170752.785	0	2500	0.000
	84	-197479.101	58175.776	25.454	-169995.437	0	2500	0.000	-172742.329	0	2500	0.000
	85	-200487.709	59077.766	25.781	-172784.836	0	2500	0.000	-175577.248	0	2500	0.000
	86	-214507.740	65451.979	28.132	-184303.608	0	2500	0.000	-187236.374	0	2500	0.000
	87	-198859.727	60524.668	25.246	-171094.426	0	2500	0.000	-173833.963	0	2500	0.000
	88	-210261.776	63693.741	26.747	-180878.474	0	2500	0.000	-183711.115	0	2500	0.000
	89	-201892.732	60071.593	26.285	-173490.520	0	2500	0.000	-176378.750	0	2500	0.000
	90	-196633.457	55411.572	25.089	-169511.329	0	2500	0.000	-172234.774	0	2500	0.000
	91	-199500.928	55749.623	25.729	-172063.273	0	2500	0.000	-174844.576	0	2500	0.000
	92	-215272.598	69483.229	28.002	-184854.546	0	2500	0.000	-187816.992	0	2500	0.000
	93	-203701.807	59086.438	25.794	-175860.215	0	2500	0.000	-178624.225	0	2500	0.000
	94	-196111.010	56939.131	24.724	-168710.189	0	2500	0.000	-171372.112	0	2500	0.000
	95	-200479.207	58261.205	26.047	-172677.959	0	2500	0.000	-175536.988	0	2500	0.000
	96	-186550.673	52570.145	23.577	-160916.080	0	2500	0.000	-163512.728	0	2500	0.000
	97	-209964.113	62357.857	27.538	-180953.501	0	2500	0.000	-183872.365	0	2500	0.000
	98	-201886.580	60054.401	25.350	-173725.086	0	2500	0.000	-176444.097	0	2500	0.000
	99	-211877.170	60902.409	27.212	-182516.279	0	2500	0.000	-185474.563	0	2500	0.000
	100	-218206.503	66165.653	28.425	-188099.754	0	2500	0.000	-191124.386	0	2500	0.000
**Mean**		-203772.943	61250.292	26.107	-175411.478	0.0	2495.9	0.000	-178216.446	0.0	2495.9	0.000
**Std. Err.**		758.282	374.891	0.118	644.373	0.0	4.1	0.000	654.522	0.0	4.1	0.000

* The column legends are the same as Table S2.
