# Supplementary material for: Cross-Scale Analyses of Animal and Human Gut Microbiome Assemblies from Metacommunity to Global Landscape
Source: mSystems. 2021 Jul 6;6(4):e00633-21. doi: 10.1128/mSystems.00633-21 (PMC8407200; doi:10.1128/mSystems.00633-21)
Supplement: TABLE S6 [file msystems.00633-21-st006.pdf]

Table S6. Fitting the MSN model at the microbiome global landscape level (or animal host kingdom level): 100 AGM samples were randomly taken (one from each of the 153 animal host species with PT information available), treated as a super-super-metacommunity representing for the animal kingdom, and used to fit the MSN model. The fitting process was repeated for 100 times by 100 times of re-sampling, and therefore a total of 100 MSN models were built at the global landscape level (Due to the computational limitation, the samples with more than 30,000 reads were excluded occasionally)*.

Sampling No.	L_O	θ	*M-value*	*Metacommunity*				*Local community*			
				L_M	N_M	N	P_M	L_L	N_L	N	P_L
1	-171602.076	42550.108	23.243	-147163.324	3	2499	0.001	-149753.929	3	2499	0.001
2	-159930.257	40959.421	21.535	-137209.861	2	2500	0.001	-139551.642	2	2500	0.001
3	-178075.806	49552.623	23.878	-153006.191	0	2500	0.000	-155587.682	0	2500	0.000
4	-157760.381	44530.758	21.145	-135554.747	1	2496	0.000	-137817.745	1	2496	0.000
5	-148303.759	46931.297	19.562	-127282.677	2	2497	0.001	-129308.742	2	2497	0.001
6	-169129.024	40688.632	23.209	-145712.269	2	2500	0.001	-148221.891	2	2500	0.001
7	-165309.116	47136.296	22.055	-141962.737	2	2496	0.001	-144301.708	1	2495	0.000
8	-171339.431	38986.772	23.607	-148308.025	0	2500	0.000	-150909.306	0	2500	0.000
9	-170955.225	53484.555	23.018	-146876.260	0	2497	0.000	-149163.472	0	2497	0.000
10	-180348.053	48999.383	24.365	-154635.673	0	2500	0.000	-157278.591	0	2500	0.000
11	-155991.046	45819.729	20.471	-134457.487	0	2500	0.000	-136595.167	0	2500	0.000
12	-172936.135	47302.951	23.048	-148640.245	2	2496	0.001	-151179.763	3	2496	0.001
13	-158205.782	36875.641	21.328	-136052.157	0	2500	0.000	-138436.793	0	2500	0.000
14	-186869.113	64568.549	25.185	-160212.903	1	2500	0.000	-162663.988	1	2500	0.000
15	-166148.469	40806.897	21.786	-143408.620	1	2498	0.000	-145822.111	1	2498	0.000
16	-142511.043	36350.318	18.397	-122630.089	1	2500	0.000	-124725.441	1	2500	0.000
17	-150226.054	40821.885	20.018	-128629.300	1	2497	0.000	-130732.281	1	2497	0.000
18	-173387.439	46184.738	23.985	-149599.312	2	2500	0.001	-152173.722	2	2500	0.001
19	-159097.664	34391.645	21.446	-137723.900	1	2498	0.000	-140154.076	1	2498	0.000
20	-170182.135	38333.137	23.271	-147295.640	1	2499	0.000	-149826.325	2	2499	0.001
21	-175571.629	47345.388	23.335	-151090.263	1	2499	0.000	-153639.046	1	2499	0.000
22	-161765.403	47999.862	20.871	-138529.646	1	2498	0.000	-140800.984	1	2498	0.000
23	-187993.921	54509.247	25.574	-161044.994	3	2498	0.001	-163633.242	3	2498	0.001
24	-175334.458	39787.774	23.796	-151728.258	2	2500	0.001	-154352.131	2	2500	0.001
25	-167399.768	49141.138	22.329	-143830.340	0	2500	0.000	-146108.331	0	2500	0.000
26	-186835.403	62382.920	24.936	-160234.417	0	2499	0.000	-162649.430	0	2499	0.000
27	-173136.184	49680.221	23.216	-148632.667	0	2499	0.000	-151100.365	0	2499	0.000
28	-167639.105	48061.729	21.851	-143930.814	0	2500	0.000	-146232.014	0	2500	0.000
29	-174486.162	40972.040	24.278	-150469.752	0	2496	0.000	-153188.116	0	2496	0.000
30	-174682.158	43700.686	23.542	-150652.813	0	2499	0.000	-153263.969	0	2499	0.000
31	-198928.212	48228.853	27.427	-171234.798	1	2496	0.000	-174228.823	1	2496	0.000
32	-183233.744	44987.475	25.251	-157687.027	2	2500	0.001	-160393.492	2	2500	0.001
33	-129408.075	32278.302	16.945	-111335.467	1	2498	0.000	-113247.536	1	2498	0.000
34	-166626.706	41054.914	22.126	-143173.296	0	2495	0.000	-145622.307	0	2495	0.000
35	-152161.715	30336.901	20.965	-132148.661	1	2499	0.000	-134366.478	1	2499	0.000
36	-165686.537	43127.709	22.690	-142792.495	0	2500	0.000	-145192.650	0	2500	0.000
37	-185868.279	57060.041	24.258	-159609.443	0	2500	0.000	-162184.703	0	2500	0.000
38	-163128.918	47348.788	21.515	-139819.108	0	2498	0.000	-142219.014	0	2498	0.000

39	-168114.836	41204.255	22.763	-144744.982	2	2497	0.001	-147233.132	2	2497	0.001
40	-169114.481	36881.446	23.151	-145590.213	0	2500	0.000	-148256.691	0	2500	0.000
41	-186385.357	58002.720	26.077	-159756.159	2	2495	0.001	-162357.599	2	2495	0.001
42	-149439.820	39036.639	20.450	-128622.643	0	2497	0.000	-130825.076	0	2497	0.000
43	-167156.176	40763.431	23.238	-144283.995	1	2499	0.000	-146818.532	1	2499	0.000
44	-190510.057	50889.496	26.710	-163772.120	2	2499	0.001	-166657.682	2	2499	0.001
45	-165711.414	57342.859	22.054	-141848.145	0	2500	0.000	-144037.729	0	2500	0.000
46	-157885.394	36979.001	21.483	-136499.527	1	2497	0.000	-138815.572	2	2498	0.001
47	-175323.871	54151.717	23.430	-150407.497	0	2500	0.000	-152810.792	0	2500	0.000
48	-192242.862	49049.181	25.987	-165668.317	1	2497	0.000	-168573.668	1	2497	0.000
49	-189596.690	61255.246	25.560	-163083.254	0	2500	0.000	-165579.879	0	2500	0.000
50	-171629.059	35173.769	24.178	-148464.398	2	2499	0.001	-151107.547	2	2499	0.001
51	-167260.366	50964.993	22.159	-143004.467	0	2497	0.000	-145353.977	0	2497	0.000
52	-167864.558	40604.905	22.627	-145107.356	0	2500	0.000	-147649.041	0	2500	0.000
53	-167766.405	42501.054	22.723	-144343.490	2	2497	0.001	-146904.891	2	2497	0.001
54	-168742.697	49281.387	22.567	-145032.488	0	2500	0.000	-147416.623	0	2500	0.000
55	-170928.115	44813.812	22.214	-147100.877	1	2497	0.000	-149624.622	1	2497	0.000
56	-169776.889	41102.485	23.312	-146820.010	1	2500	0.000	-149359.086	2	2500	0.001
57	-163074.361	50035.012	22.081	-139890.355	0	2500	0.000	-142138.947	0	2500	0.000
58	-154563.051	35763.964	21.164	-133350.271	1	2500	0.000	-135690.411	1	2500	0.000
59	-179717.936	45298.410	25.006	-154463.324	2	2498	0.001	-157099.845	2	2498	0.001
60	-175041.466	43324.435	23.447	-150812.923	3	2500	0.001	-153462.873	3	2500	0.001
61	-181514.748	42550.123	25.239	-156506.852	0	2500	0.000	-159258.042	0	2500	0.000
62	-161029.450	46355.717	21.668	-137966.194	0	2500	0.000	-140309.355	0	2500	0.000
63	-185397.693	45827.844	25.483	-160184.789	0	2500	0.000	-162939.003	0	2500	0.000
64	-170029.303	36771.833	23.625	-146830.413	1	2495	0.000	-149426.023	1	2495	0.000
65	-176219.274	42685.871	23.633	-151735.494	0	2500	0.000	-154346.243	0	2500	0.000
66	-156476.864	42493.432	20.572	-134191.559	1	2498	0.000	-136408.888	1	2498	0.000
67	-164072.661	38049.699	22.597	-141810.526	1	2500	0.000	-144301.575	1	2500	0.000
68	-184775.631	48285.684	25.004	-159202.121	3	2496	0.001	-161961.816	4	2496	0.002
69	-151352.218	42905.992	19.730	-130985.016	0	2500	0.000	-133136.252	0	2500	0.000
70	-160965.884	43643.169	21.575	-138654.152	0	2499	0.000	-140997.973	0	2499	0.000
71	-165918.865	42158.528	23.393	-143158.710	1	2497	0.000	-145655.692	1	2497	0.000
72	-167290.387	37863.959	22.588	-144177.362	3	2499	0.001	-146658.908	3	2499	0.001
73	-158647.285	35635.837	21.778	-136626.555	0	2500	0.000	-139038.067	0	2500	0.000
74	-167334.034	52886.570	22.412	-144009.924	1	2497	0.000	-146377.410	1	2497	0.000
75	-153701.728	45687.802	21.062	-131661.351	0	2499	0.000	-133918.165	0	2499	0.000
76	-174937.705	49619.248	23.484	-150217.521	2	2498	0.001	-152691.747	2	2498	0.001
77	-186804.952	55388.049	24.499	-159901.247	1	2491	0.000	-162523.885	1	2491	0.000
78	-152649.693	30684.039	20.960	-131934.366	0	2219	0.000	-134223.247	0	2219	0.000
79	-181824.342	47652.623	24.514	-156320.916	2	2497	0.001	-159018.468	2	2497	0.001
80	-172421.137	44083.889	22.989	-148382.212	1	2497	0.000	-150919.001	1	2497	0.000
81	-164816.443	43185.721	22.227	-142255.853	3	2494	0.001	-144641.091	3	2494	0.001
82	-161874.744	42463.668	21.177	-139237.646	2	2499	0.001	-141521.412	1	2498	0.000
83	-171329.575	38336.089	23.395	-147894.410	1	2500	0.000	-150451.549	1	2500	0.000
84	-179108.594	38532.750	24.595	-154724.422	0	2498	0.000	-157466.729	0	2498	0.000
85	-161931.585	47926.429	21.832	-139601.143	3	2497	0.001	-141868.258	3	2497	0.001

86	-164910.876	39612.538	22.648	-142418.540	4	2495	0.002	-144940.007	3	2494	0.001
87	-161779.915	47912.879	21.864	-138748.272	2	2497	0.001	-140962.399	2	2497	0.001
88	-162074.523	37836.151	22.406	-139808.432	0	2500	0.000	-142261.873	0	2500	0.000
89	-191538.400	50966.269	26.728	-165173.904	3	2496	0.001	-168157.735	2	2496	0.001
90	-157228.947	36589.536	21.084	-135754.443	3	2494	0.001	-138167.619	3	2494	0.001
91	-185836.576	46532.154	25.400	-160340.334	2	2500	0.001	-163051.032	2	2500	0.001
92	-164304.978	41430.308	22.074	-141541.676	0	2500	0.000	-143884.724	0	2500	0.000
93	-171399.676	40173.962	23.399	-147835.289	2	2498	0.001	-150452.394	2	2498	0.001
94	-168502.348	39858.368	23.961	-145693.792	0	2497	0.000	-148250.406	0	2497	0.000
95	-169889.358	36368.151	23.719	-147184.331	0	2500	0.000	-149816.292	0	2500	0.000
96	-175385.620	49128.412	23.328	-151362.381	0	2496	0.000	-153923.833	0	2496	0.000
97	-176025.375	49766.360	23.751	-150630.632	4	2494	0.002	-153220.924	4	2494	0.002
98	-176271.186	43667.941	24.228	-152274.629	0	2496	0.000	-154873.445	0	2496	0.000
99	-179614.118	40877.422	25.537	-154953.586	0	2500	0.000	-157724.119	0	2500	0.000
100	-161736.391	43905.483	21.868	-138413.034	0	2500	0.000	-140791.589	0	2500	0.000
**Mean**	-169449.633	44440.000	22.939	-145872.765	1.01	2495.5		-148349.4	1.02	2495.5	
**Std. Err.**	1174.447	664.452	0.179	1008.386	0.109	2.799		1025.792	0.109	2.799	
**Passing Rate (%)**							0				0

* The column legends are the same as Table S2.
