# Supplementary material for: Cross-Scale Analyses of Animal and Human Gut Microbiome Assemblies from Metacommunity to Global Landscape
Source: mSystems. 2021 Jul 6;6(4):e00633-21. doi: 10.1128/mSystems.00633-21 (PMC8407200; doi:10.1128/mSystems.00633-21)
Supplement: TABLE S7 [file msystems.00633-21-st007.pdf]

Table S7. The *Spearman*'s correlation coefficients (*R*) between the MSN model parameters (θ, M**)** and the PT (phylogenetic timeline) of 179 host animal species or 10 animal classes

**Host Taxon Level**	***M vs.* PT**		***θ vs.* PT**		***M vs. θ***	
	R	***p*-value**	***R***	***p*-value**	***R***	***p*-value**
AGM (Animal gut microbiome)						
Species	-0.326	0.000	-0.122	0.105	0.163	0.030
Class	-0.251	0.515	-0.469	0.203	0.189	0.608
AGM + AGP (American Gut microbiome Project) + CGP (Chinese Gut microbiome Project)						
Species	-0.335	0.000	-0.115	0.124	0.151	0.043
