# Supplementary material for: Cross-Scale Analyses of Animal and Human Gut Microbiome Assemblies from Metacommunity to Global Landscape
Source: mSystems. 2021 Jul 6;6(4):e00633-21. doi: 10.1128/mSystems.00633-21 (PMC8407200; doi:10.1128/mSystems.00633-21)
Supplement: TABLE S8 [file msystems.00633-21-st008.pdf]

Table S8. The classification table from the logistic regression (LR) analysis of the MSN parameters at the host species level [*X*=PT, *Y*=Probability of Non-Neutral (*0*) vs. Neutral (*1*)]

	**Estimated Occurrences**		
**Actual Occurrences**	***0 (Non-neutral)***	***1 (Neutral)***	***Total***
0 (Non-neutral)	101	7	108
1 (Neutral)	55	16	71
Total	156	23	179
**Percentage Correctly Classified = 65.4%**			

Parameters from the LT of the PT-Neutrality Relationship (Reference Group Y=0 or Non-neutral)

**Variable (Factor)**	**LR Coefficient (B)**	**Standard Error of B**	***P*-value of Wald Test**	**Odds Ratio: Exp (B)**
**PD (B_1)**	0.008*	0.002	0.0016	1.007
**Intercept (B_0)**	-0.812	0.196	<0.0001	0.444

*The positive LR coefficient corresponding the PD suggests that more ancient species (with larger PD) are more likely to host non-neutral gut microbiome and more recent species such as humans are more likely to host neutral microbiomes.
