# Supplementary material for: Cross-Scale Analyses of Animal and Human Gut Microbiome Assemblies from Metacommunity to Global Landscape
Source: mSystems. 2021 Jul 6;6(4):e00633-21. doi: 10.1128/mSystems.00633-21 (PMC8407200; doi:10.1128/mSystems.00633-21)
Supplement: TABLE S3 [file msystems.00633-21-st003.pdf]

Table S3. Fitting the MSN (multisite neutral) model with the AGP (American gut microbiome project) datasets: 100 AGP samples were taken randomly from 1474 individuals, treated as a metacommunity and fitted to a MSN model. The process was repeated 100 times, and a total of 100 MSN models were obtained*.

Sampling No.	L_O	θ	*M-value*	*Metacommunity*				*Local community*			
				L_M	N_M	N	p_M	L_L	N_L	N	p_L
1	-297675.578	1193.879	173.600	-401021.282	2455	2455	1.000	-332224.745	2455	2455	1.000
2	-280393.038	1264.983	161.680	-380849.138	2455	2455	1.000	-311470.870	2455	2455	1.000
3	-297718.656	1299.127	172.929	-409927.176	2455	2455	1.000	-332169.957	2455	2455	1.000
4	-294802.917	1307.393	159.079	-390489.937	2455	2455	1.000	-328947.947	2455	2455	1.000
5	-276875.559	1200.573	156.067	-372925.587	2455	2455	1.000	-309010.209	2455	2455	1.000
6	-285529.358	1303.809	148.414	-376070.921	2455	2455	1.000	-319938.882	2455	2455	1.000
7	-289176.509	1254.823	163.108	-385054.097	2410	2410	1.000	-322215.536	2410	2410	1.000
8	-292446.338	1267.778	161.384	-390760.364	2455	2455	1.000	-325819.175	2455	2455	1.000
9	-280587.633	1323.567	152.546	-374160.262	2455	2455	1.000	-312323.506	2455	2455	1.000
10	-286561.372	1280.695	153.065	-380899.039	2455	2455	1.000	-320782.498	2455	2455	1.000
11	-287594.622	1352.370	153.493	-384628.740	2455	2455	1.000	-321649.286	2455	2455	1.000
12	-283039.168	1265.290	158.112	-382810.027	2455	2455	1.000	-316299.819	2455	2455	1.000
13	-286032.466	1263.136	158.678	-383047.811	2410	2410	1.000	-319033.263	2410	2410	1.000
14	-295988.615	1254.401	168.721	-396526.148	2500	2500	1.000	-329756.954	2500	2500	1.000
15	-289822.475	1333.524	151.427	-387091.408	2500	2500	1.000	-324792.351	2500	2500	1.000
16	-297181.501	1328.328	160.503	-393447.260	2500	2500	1.000	-331826.539	2500	2500	1.000
17	-303042.883	1332.587	173.301	-407855.173	2500	2500	1.000	-337089.777	2500	2500	1.000
18	-295116.290	1265.274	171.020	-401778.020	2500	2500	1.000	-329143.011	2500	2500	1.000
19	-286841.894	1243.043	158.466	-384394.591	2500	2500	1.000	-319836.968	2500	2500	1.000
20	-289816.088	1290.038	163.438	-390787.087	2500	2500	1.000	-323024.999	2500	2500	1.000
21	-303445.384	1290.381	170.548	-406930.342	2500	2500	1.000	-338511.013	2500	2500	1.000
22	-285369.649	1271.439	164.299	-387108.446	2454	2454	1.000	-317938.121	2454	2454	1.000
23	-286799.664	1303.935	158.349	-384503.352	2500	2500	1.000	-319748.100	2500	2500	1.000
24	-294160.218	1269.464	166.818	-396059.215	2500	2500	1.000	-328205.583	2500	2500	1.000
25	-278943.668	1278.627	145.355	-366994.506	2500	2500	1.000	-311621.159	2500	2500	1.000
26	-286163.013	1233.798	162.342	-383499.720	2500	2500	1.000	-318485.139	2500	2500	1.000
27	-297820.925	1231.660	173.250	-399074.917	2500	2500	1.000	-330962.995	2500	2500	1.000
28	-285910.354	1255.399	163.471	-385700.346	2500	2500	1.000	-318016.432	2500	2500	1.000
29	-287421.239	1215.652	163.605	-385826.296	2500	2500	1.000	-320515.453	2500	2500	1.000
30	-276529.233	1278.192	150.061	-369911.533	2500	2500	1.000	-308638.596	2500	2500	1.000
31	-294825.658	1267.187	170.341	-395798.869	2500	2500	1.000	-327327.967	2500	2500	1.000
32	-282292.137	1223.517	162.700	-378249.948	2500	2500	1.000	-313914.723	2500	2500	1.000
33	-288177.798	1227.769	162.710	-387109.629	2409	2409	1.000	-320785.916	2409	2409	1.000
34	-305374.578	1331.330	168.461	-410828.349	2500	2500	1.000	-341555.403	2500	2500	1.000
35	-284550.258	1273.106	155.212	-382643.750	2500	2500	1.000	-317632.060	2500	2500	1.000
36	-288158.521	1253.306	161.057	-388715.175	2500	2500	1.000	-321560.929	2500	2500	1.000
37	-290152.950	1214.914	160.368	-385498.004	2500	2500	1.000	-324137.339	2500	2500	1.000
38	-277447.932	1254.415	151.915	-373194.359	2500	2500	1.000	-309814.598	2500	2500	1.000
39	-278824.570	1197.952	160.146	-376340.487	2500	2500	1.000	-309677.603	2500	2500	1.000
40	-287392.180	1204.540	173.685	-393264.945	2500	2500	1.000	-319284.784	2500	2500	1.000
41	-308296.837	1287.560	173.120	-409681.716	2500	2500	1.000	-342585.654	2500	2500	1.000

42	-285211.843	1230.611	167.344	-389141.381	2500	2500	1.000	-317347.610	2500	2500	1.000
43	-280187.921	1278.284	146.289	-371925.691	2500	2500	1.000	-313317.099	2500	2500	1.000
44	-280765.746	1240.584	163.796	-384574.182	2410	2410	1.000	-312746.174	2410	2410	1.000
45	-296499.719	1333.757	156.550	-391291.129	2500	2500	1.000	-331580.470	2500	2500	1.000
46	-281772.047	1257.628	164.701	-381729.072	2409	2410	1.000	-313479.940	2410	2410	1.000
47	-272613.171	1326.904	142.536	-359858.717	2455	2455	1.000	-304191.057	2455	2455	1.000
48	-290950.488	1308.597	161.264	-388455.230	2455	2455	1.000	-323929.473	2455	2455	1.000
49	-288802.079	1274.174	164.702	-386323.510	2454	2455	1.000	-321542.354	2455	2455	1.000
50	-282090.477	1243.296	165.594	-385384.030	2409	2410	1.000	-313512.691	2410	2410	1.000
51	-287960.847	1281.293	168.231	-393035.229	2500	2500	1.000	-320043.242	2500	2500	1.000
52	-284632.832	1217.642	169.288	-391249.479	2500	2500	1.000	-317154.438	2500	2500	1.000
53	-275826.877	1299.467	151.970	-373890.749	2500	2500	1.000	-308114.731	2500	2500	1.000
54	-279761.489	1211.107	161.940	-378297.819	2500	2500	1.000	-311171.963	2500	2500	1.000
55	-282258.809	1199.387	163.631	-380315.612	2500	2500	1.000	-314838.498	2500	2500	1.000
56	-297213.611	1263.001	169.659	-400689.343	2500	2500	1.000	-331496.445	2500	2500	1.000
57	-294138.530	1311.002	166.359	-396594.237	2500	2500	1.000	-327388.319	2500	2500	1.000
58	-292420.832	1264.236	160.996	-388388.178	2500	2500	1.000	-326008.697	2500	2500	1.000
59	-285423.232	1213.952	168.546	-388506.112	2500	2500	1.000	-318644.420	2500	2500	1.000
60	-294687.159	1237.971	172.177	-398696.484	2500	2500	1.000	-327692.043	2500	2500	1.000
61	-291622.285	1222.444	167.948	-387537.371	2500	2500	1.000	-324504.573	2500	2500	1.000
62	-292251.760	1296.217	162.968	-392206.567	2500	2500	1.000	-325016.861	2500	2500	1.000
63	-299525.746	1294.723	165.558	-403117.305	2500	2500	1.000	-334744.278	2500	2500	1.000
64	-288481.465	1295.392	163.260	-392266.672	2500	2500	1.000	-321829.144	2500	2500	1.000
65	-290341.353	1277.697	163.873	-387167.580	2500	2500	1.000	-322548.426	2500	2500	1.000
66	-293601.287	1296.168	164.533	-392822.649	2500	2500	1.000	-326333.551	2500	2500	1.000
67	-278498.347	1297.447	148.650	-374537.192	2500	2500	1.000	-311579.213	2500	2500	1.000
68	-284887.194	1338.891	158.828	-386201.243	2500	2500	1.000	-317645.550	2500	2500	1.000
69	-275977.289	1250.438	153.573	-370664.681	2500	2500	1.000	-308077.051	2500	2500	1.000
70	-288637.574	1258.818	166.238	-389649.533	2500	2500	1.000	-320410.322	2500	2500	1.000
71	-291598.083	1202.297	170.813	-392895.406	2500	2500	1.000	-324762.726	2500	2500	1.000
72	-277122.443	1234.575	149.165	-367172.877	2500	2500	1.000	-309880.533	2500	2500	1.000
73	-296780.238	1251.159	172.000	-403170.295	2500	2500	1.000	-330809.601	2500	2500	1.000
74	-291973.682	1231.311	162.885	-383785.457	2500	2500	1.000	-324330.244	2500	2500	1.000
75	-277634.495	1202.702	161.615	-376714.358	2500	2500	1.000	-308761.984	2500	2500	1.000
76	-302698.048	1289.889	173.219	-404665.474	2500	2500	1.000	-336083.201	2500	2500	1.000
77	-296785.838	1266.112	168.856	-399337.201	2500	2500	1.000	-330887.643	2500	2500	1.000
78	-293310.202	1215.169	175.238	-402838.450	2500	2500	1.000	-326915.946	2500	2500	1.000
79	-283190.001	1293.461	158.036	-382055.765	2500	2500	1.000	-315471.825	2500	2500	1.000
80	-301484.607	1292.205	168.916	-398678.088	2500	2500	1.000	-335204.359	2500	2500	1.000
81	-288361.147	1300.492	160.756	-392456.675	2500	2500	1.000	-322651.481	2500	2500	1.000
82	-279612.325	1221.674	160.881	-381530.735	2500	2500	1.000	-311316.457	2500	2500	1.000
83	-278693.047	1233.513	152.123	-369092.561	2500	2500	1.000	-310946.225	2500	2500	1.000
84	-283173.936	1241.112	161.832	-378001.301	2500	2500	1.000	-314809.540	2500	2500	1.000
85	-293472.173	1280.999	161.752	-390546.927	2500	2500	1.000	-327072.426	2500	2500	1.000
86	-297047.712	1229.873	177.562	-404401.250	2500	2500	1.000	-330931.670	2500	2500	1.000
87	-305495.119	1297.804	166.410	-400931.067	2500	2500	1.000	-339975.666	2500	2500	1.000
88	-288408.672	1260.985	160.302	-393073.746	2500	2500	1.000	-322242.569	2500	2500	1.000

89	-299551.675	1226.320	171.061	-397411.932	2500	2500	1.000	-333109.499	2500	2500	1.000
90	-276654.351	1221.530	159.296	-378771.814	2500	2500	1.000	-308394.172	2500	2500	1.000
91	-280092.615	1231.796	154.198	-368852.686	2500	2500	1.000	-311377.473	2500	2500	1.000
92	-282613.828	1227.611	156.131	-374706.520	2500	2500	1.000	-315161.003	2500	2500	1.000
93	-286588.456	1274.796	157.039	-382219.887	2500	2500	1.000	-319266.528	2500	2500	1.000
94	-285298.655	1243.093	164.037	-385325.510	2500	2500	1.000	-317893.582	2500	2500	1.000
95	-283913.017	1263.060	157.984	-381829.105	2500	2500	1.000	-316469.268	2500	2500	1.000
96	-292796.673	1227.956	178.098	-405178.469	2500	2500	1.000	-324901.435	2500	2500	1.000
97	-283917.749	1245.996	155.788	-377458.540	2500	2500	1.000	-317032.862	2500	2500	1.000
98	-288280.579	1304.979	164.518	-396353.276	2500	2500	1.000	-322131.318	2500	2500	1.000
99	-285427.689	1322.123	155.699	-379409.429	2500	2500	1.000	-318230.043	2500	2500	1.000
100	-292821.000	1314.738	168.405	-400114.952	2500	2500	1.000	-325888.978	2500	2500	1.000
**Mean**	-288461.398	1264.592	162.425	-387749.547	2487.8	2487.8		-321480.747	2487.83	2487.8	
**Std. Err.**	769.882	3.829	0.747	1089.447	2.559	2.551		849.150	2.551	2.551	
**Passing Rate (%)**							100%				100%

* The column legends are the same as Table S2.
