# Supplementary material for: Cross-Scale Analyses of Animal and Human Gut Microbiome Assemblies from Metacommunity to Global Landscape
Source: mSystems. 2021 Jul 6;6(4):e00633-21. doi: 10.1128/mSystems.00633-21 (PMC8407200; doi:10.1128/mSystems.00633-21)
Supplement: TABLE S4 [file msystems.00633-21-st004.pdf]

Table S4. Fitting the MSN (multisite neutral) model with the CGP (Chinese gut microbiome project) datasets: 100 CGP samples were taken randomly from 314 individuals, treated as a metacommunity and fitted to a MSN model. The process was repeated 100 times, and a total of 100 MSN models were obtained*.

Sampling No.	L_O	θ	*M-value*	*Metacommunity*				*Local community*			
				L_M	N_M	N	p_M	L_L	N_L	N	p_L
1	-165096.086	283.565	227.354	-230787.596	2500	2500	1.000	-176658.714	2500	2500	1.000
2	-164243.387	289.871	210.167	-230207.464	2500	2500	1.000	-176082.952	2500	2500	1.000
3	-170016.870	293.661	233.308	-242958.089	2500	2500	1.000	-181825.232	2500	2500	1.000
4	-163260.368	285.908	219.858	-231425.530	2500	2500	1.000	-174769.155	2500	2500	1.000
5	-169157.406	299.394	225.332	-240236.643	2500	2500	1.000	-181054.293	2500	2500	1.000
6	-166945.197	286.735	212.204	-231396.059	2500	2500	1.000	-179168.696	2500	2500	1.000
7	-167356.642	297.056	224.599	-239600.097	2500	2500	1.000	-179359.172	2500	2500	1.000
8	-161407.443	281.434	210.058	-226162.124	2500	2500	1.000	-172865.701	2500	2500	1.000
9	-164860.318	287.580	224.655	-231819.272	2500	2500	1.000	-176224.747	2500	2500	1.000
10	-171281.977	297.676	231.789	-242171.125	2500	2500	1.000	-183675.271	2500	2500	1.000
11	-173995.182	306.342	250.628	-250499.747	2500	2500	1.000	-186116.612	2500	2500	1.000
12	-163115.762	280.678	207.580	-227928.599	2500	2500	1.000	-174898.786	2500	2500	1.000
13	-163947.130	285.745	214.172	-230497.860	2500	2500	1.000	-175513.621	2500	2500	1.000
14	-165443.562	281.142	220.054	-230971.428	2500	2500	1.000	-177218.706	2500	2500	1.000
15	-163585.123	284.121	210.601	-230870.822	2500	2500	1.000	-175317.171	2500	2500	1.000
16	-168610.504	284.350	245.883	-238046.679	2500	2500	1.000	-180199.430	2500	2500	1.000
17	-165741.290	284.433	227.452	-236502.856	2500	2500	1.000	-177529.648	2500	2500	1.000
18	-166181.947	282.697	227.707	-234855.476	2500	2500	1.000	-177946.064	2500	2500	1.000
19	-169887.577	298.416	253.900	-245271.087	2500	2500	1.000	-181297.069	2500	2500	1.000
20	-163705.277	297.386	200.875	-231958.489	2500	2500	1.000	-176000.035	2500	2500	1.000
21	-165236.174	297.385	224.411	-235693.792	2500	2500	1.000	-176777.763	2500	2500	1.000
22	-164624.039	290.218	199.296	-229534.014	2500	2500	1.000	-176878.428	2500	2500	1.000
23	-167028.721	292.340	215.597	-235470.580	2500	2500	1.000	-179022.761	2500	2500	1.000
24	-163127.165	287.320	199.916	-231843.294	2500	2500	1.000	-175336.275	2500	2500	1.000
25	-163810.678	287.056	228.119	-232130.077	2500	2500	1.000	-175374.528	2500	2500	1.000
26	-171236.272	296.805	243.700	-244063.589	2500	2500	1.000	-183154.705	2500	2500	1.000
27	-171030.407	295.476	236.958	-242682.401	2500	2500	1.000	-183566.816	2500	2500	1.000
28	-168604.176	298.614	216.973	-238915.397	2500	2500	1.000	-180879.082	2500	2500	1.000
29	-167602.806	299.329	229.290	-239286.370	2500	2500	1.000	-179468.168	2500	2500	1.000
30	-167829.613	289.143	225.207	-237217.817	2500	2500	1.000	-180261.809	2500	2500	1.000
31	-166998.570	286.069	232.225	-237903.031	2500	2500	1.000	-178780.287	2500	2500	1.000
32	-168174.729	291.946	227.602	-237803.120	2500	2500	1.000	-180327.285	2500	2500	1.000
33	-163197.242	297.270	242.107	-237490.402	2500	2500	1.000	-174514.918	2500	2500	1.000
34	-160466.175	288.305	193.312	-224865.945	2500	2500	1.000	-172079.287	2500	2500	1.000
35	-164433.653	305.477	227.617	-237240.540	2500	2500	1.000	-175875.038	2500	2500	1.000
36	-164636.492	289.716	211.942	-232667.213	2500	2500	1.000	-176751.834	2500	2500	1.000
37	-165955.601	279.890	206.097	-228902.686	2500	2500	1.000	-178027.191	2500	2500	1.000
38	-166410.080	302.739	230.585	-239190.628	2500	2500	1.000	-178306.202	2500	2500	1.000
39	-167352.819	296.615	214.704	-237629.462	2500	2500	1.000	-179568.759	2500	2500	1.000
40	-165625.752	290.246	204.196	-231829.949	2500	2500	1.000	-177803.649	2500	2500	1.000
41	-163596.107	281.059	230.209	-231184.501	2500	2500	1.000	-174858.221	2500	2500	1.000

42	-169959.949	287.263	251.406	-240494.464	2500	2500	1.000	-181739.815	2500	2500	1.000
43	-167603.889	281.589	227.386	-236148.392	2500	2500	1.000	-179355.291	2500	2500	1.000
44	-164478.988	284.127	218.423	-232127.996	2500	2500	1.000	-175852.119	2500	2500	1.000
45	-165855.418	284.490	239.386	-236169.941	2500	2500	1.000	-177440.903	2500	2500	1.000
46	-161419.233	290.512	214.945	-227636.130	2500	2500	1.000	-172602.914	2500	2500	1.000
47	-166469.035	308.225	213.852	-240131.106	2500	2500	1.000	-178570.150	2500	2500	1.000
48	-165097.352	288.857	215.489	-231419.620	2500	2500	1.000	-177049.785	2500	2500	1.000
49	-165499.523	289.318	232.262	-236296.610	2500	2500	1.000	-177285.832	2500	2500	1.000
50	-162456.834	297.450	191.954	-229288.103	2500	2500	1.000	-174889.621	2500	2500	1.000
51	-163557.561	286.294	199.267	-228228.201	2500	2500	1.000	-175853.470	2500	2500	1.000
52	-163634.987	274.645	212.146	-226185.184	2500	2500	1.000	-175433.038	2500	2500	1.000
53	-166917.117	282.627	235.828	-234790.034	2500	2500	1.000	-178678.343	2500	2500	1.000
54	-162550.681	288.643	210.627	-231279.884	2500	2500	1.000	-174405.890	2500	2500	1.000
55	-167782.333	291.598	249.010	-240071.455	2500	2500	1.000	-179263.831	2500	2500	1.000
56	-167159.557	286.006	239.676	-236110.328	2500	2500	1.000	-178182.256	2500	2500	1.000
57	-165483.966	305.733	214.211	-237373.463	2500	2500	1.000	-177582.376	2500	2500	1.000
58	-165449.500	289.718	219.224	-232326.530	2500	2500	1.000	-177185.537	2500	2500	1.000
59	-166563.058	282.980	227.429	-235921.623	2500	2500	1.000	-178306.071	2500	2500	1.000
60	-166417.471	293.646	223.967	-236379.278	2500	2500	1.000	-178664.547	2500	2500	1.000
61	-168131.914	291.632	244.651	-239378.067	2500	2500	1.000	-179582.143	2500	2500	1.000
62	-164423.435	269.002	230.777	-227982.781	2500	2500	1.000	-175965.564	2500	2500	1.000
63	-167164.028	290.284	226.624	-238403.830	2500	2500	1.000	-179237.844	2500	2500	1.000
64	-165236.018	294.731	220.523	-235080.510	2500	2500	1.000	-177175.641	2500	2500	1.000
65	-169807.748	298.191	232.063	-242174.463	2500	2500	1.000	-182036.390	2500	2500	1.000
66	-165075.913	296.181	235.391	-234971.527	2500	2500	1.000	-176706.381	2500	2500	1.000
67	-166041.989	290.586	208.485	-234827.616	2500	2500	1.000	-178716.871	2500	2500	1.000
68	-166092.561	286.091	230.577	-232458.186	2500	2500	1.000	-177461.200	2500	2500	1.000
69	-169303.384	290.207	233.062	-239972.342	2500	2500	1.000	-181175.152	2500	2500	1.000
70	-166163.280	296.012	216.618	-235018.455	2500	2500	1.000	-178203.677	2500	2500	1.000
71	-169774.084	289.030	227.697	-239074.261	2500	2500	1.000	-181917.481	2500	2500	1.000
72	-162729.530	284.732	211.988	-228713.676	2500	2500	1.000	-174238.262	2500	2500	1.000
73	-161104.267	284.468	211.316	-228766.288	2500	2500	1.000	-172534.137	2500	2500	1.000
74	-168714.422	289.321	230.144	-238352.435	2500	2500	1.000	-180724.848	2500	2500	1.000
75	-165257.553	282.179	231.725	-231905.448	2500	2500	1.000	-176968.464	2500	2500	1.000
76	-165085.245	293.100	217.528	-237544.478	2500	2500	1.000	-177258.758	2500	2500	1.000
77	-168325.708	281.388	221.238	-233385.390	2500	2500	1.000	-180352.070	2500	2500	1.000
78	-168963.707	295.385	242.716	-243356.493	2500	2500	1.000	-180761.873	2500	2500	1.000
79	-167433.246	290.139	259.455	-241514.413	2500	2500	1.000	-178961.014	2500	2500	1.000
80	-158809.981	293.066	188.639	-222908.005	2500	2500	1.000	-170929.689	2500	2500	1.000
81	-171217.256	285.674	221.420	-239548.876	2500	2500	1.000	-183663.665	2500	2500	1.000
82	-165825.633	296.596	217.763	-236170.398	2500	2500	1.000	-177571.215	2500	2500	1.000
83	-166116.946	287.664	239.436	-235918.031	2500	2500	1.000	-177450.034	2500	2500	1.000
84	-167813.530	293.446	229.420	-240276.573	2500	2500	1.000	-179856.645	2500	2500	1.000
85	-159731.292	286.425	197.723	-223595.497	2500	2500	1.000	-171514.274	2500	2500	1.000
86	-167801.173	281.971	228.775	-232284.473	2500	2500	1.000	-179700.192	2500	2500	1.000
87	-163966.572	277.804	225.875	-230836.785	2500	2500	1.000	-175740.711	2500	2500	1.000
88	-166543.555	297.603	217.762	-236921.019	2500	2500	1.000	-178634.026	2500	2500	1.000

89	-166414.047	284.769	213.158	-233954.561	2500	2500	1.000	-178681.590	2500	2500	1.000
90	-165425.111	284.894	203.530	-230304.257	2500	2500	1.000	-177600.800	2500	2500	1.000
91	-167366.829	297.641	213.377	-235737.681	2500	2500	1.000	-179783.085	2500	2500	1.000
92	-165550.413	293.872	217.179	-235627.536	2500	2500	1.000	-177659.340	2500	2500	1.000
93	-167599.376	296.703	230.006	-240266.814	2500	2500	1.000	-179407.138	2500	2500	1.000
94	-165122.004	290.277	241.505	-235041.645	2500	2500	1.000	-176255.725	2500	2500	1.000
95	-164168.728	287.076	223.311	-231880.164	2500	2500	1.000	-175964.864	2500	2500	1.000
96	-160529.765	288.146	221.655	-229648.208	2500	2500	1.000	-171926.977	2500	2500	1.000
97	-163971.711	299.284	218.644	-235230.773	2500	2500	1.000	-175902.375	2500	2500	1.000
98	-163886.590	284.433	214.493	-229793.110	2500	2500	1.000	-175793.881	2500	2500	1.000
99	-167442.734	279.105	240.589	-235373.192	2500	2500	1.000	-178790.825	2500	2500	1.000
100	-167432.959	296.365	215.084	-238367.876	2500	2500	1.000	-179529.136	2500	2500	1.000
**Mean**	-165967.370	290.004	223.066	-234886.563	2500	2500		-177820.098	2500	2500	
**Std. Err.**	268.619	0.708	1.407	499.512	0.000	0.000		280.949	0.000	0.000	
**Passing Rate (%)**							100%				100%

* The column legends are the same as Table S2.
